# Fine-Tuning the SMM Properties of a Bis(ZnDy)-Based Compound by Rationally Reducing/Increasing the Electron Density in the Equatorial Plane Perpendicular to the O_phenoxido_–Dy‑O_phenoxido‑bridges_ Direction

**DOI:** 10.1021/acs.inorgchem.6c01258

**Published:** 2026-04-23

**Authors:** Andoni Zabala-Lekuona, Javier Cepeda, José A. García, Nina P. Gritsan, Alexey A. Dmitriev, Pablo Salcedo-Abraira, Antonio Rodríguez-Diéguez, José M. Seco, Enrique Colacio

**Affiliations:** † Departamento de Química Aplicada, Facultad de Química, 83067 Universidad del País Vasco (UPV/EHU), 20018 Donostia-San Sebastián, Spain; ‡ Departamento de Física, Facultad de Ciencia y Tecnología, Universidad del País Vasco (UPV/EHU), 48940 Leioa, Spain; § Institute of Chemical Kinetics and Combustion, Siberian Branch, Russian Academy of Sciences, 630090 Novosibirsk, Russia; ∥ Departamento de Química Inorgánica, 117396 Facultad de Ciencias, Universidad de Granada, 18071 Granada, Spain

## Abstract

Five new bis­(ZnDy) complexes with the general formula [Zn_2_(μ-H_2_L)_2_(μ-dicarb)­Dy_2_(L’)_2_(L’’)_2_]­X_2_·solv were synthesized using Mannich bis­(compartmental) ligand H_4_L (highly phenoxido-containing macrocycle). Tetrafluorosuccinate (**1**, **3**) and succinate (**2**, **4**, **5**) were used as dicarboxylates; ancillary ligands L’ = NO_3_^–^ (**1**), hfac (**2** and **3**), CF_3_CO_2_^–^/tfac (**4**), dbm (**5**); L’’ = MeOH (**4**); and counterions X = NO_3_^–^ (**1**) or OTf^–^ (**2**–**5**). All complexes have a similar axial environment defined by three phenoxido donors, while the equatorial ligand set was systematically tuned to control the electron density around Dy^III^. Magnetic measurements and *ab initio* calculations reveal a clear structure–property correlation: ligands that reduce the equatorial electron density stabilize the ground doublet *M*_J_ = ± 15/2 and provide enhanced SMM behavior and wider hysteresis loops (tetrafluorosuccinate and nitrate in **1** with *U*_Orbach_ = 370 ± 30 K). More electron-donating ligands compress the Dy–O distances and reduce magnetic anisotropy, leading to weaker or absent SMM behavior. Dy^III^-centered emission was detected in all compounds, and analysis of the 482 nm band enabled the estimation of the splitting of the ^6^H_15/2_ ground term, which matches *ab initio* results. This confirms the internal consistency between spectroscopic and theoretical descriptions and reinforces the structure–magnetism correlation for these compounds.

## Graphical abstract

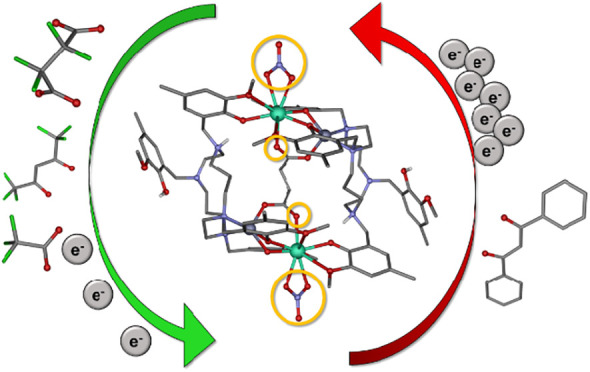

## Introduction

Since 2003, when Ishikawa reported on the single-molecule magnet (SMM) behavior of the (Bu_4_N)­[Tb­(Pc)_2_] complex,^1^ the number of studies on Ln^III^-based SMMs have steadily increased.^2–7^ The strong spin–orbit coupling and the inherent anisotropy of Ln^III^ ions surrounded by a suitable ligand field make them potential candidates for demonstrating large effective energy barriers for magnetization reversal (*U*_eff_) and open magnetic hysteresis loops at high temperatures (*T*_H_), which are two crucial characteristics in the development of high-density data storage and spintronics devices based on SMMs. Among the lanthanides, Dy^III^ and Tb^III^ ions have emerged as the most promising candidates in this field, although the Kramers character of Dy^III^ provides an advantage over Tb^III^ because of the van Vleck cancellation principle and because the doubly degeneracy of the *M*_J_ ground state (bistability) is ensured.^8^ In fact, Dy^III^-based complexes in which the axial ligand fields strictly fulfill the design criteria proposed by Rinehart and Long for oblate ions,^9^ rank highest in *U*_eff_ and blocking-temperatures (*T*_B_) among all described SMMs. This is because in this situation, the *M*_J_ = ± 15/2 sublevel in the ground ^6^H_15/2_ state is well separated from the excited states, which prevents reorientation of the magnetization.

Within this privileged family, the low-coordinated mononuclear amide-alkene [Dy­{N­(Si^*i*^Pr_3_)­[Si­(^*i*^Pr)_2_C­(CH_3_)=CHCH_3_]}­{N­(Si^*i*^Pr_3_)­(Si^*i*^Pr_2_Et)}]­[Al­{OC­(CF_3_)_3_}_4_] compound and metallocene [DyC_5_Me_5_(Cp^iPr5^)]­[B­(C_6_F_5_)_4_] and the mixed-valence Dy^III^Dy^II^ dinuclear [Dy_2_I_3_(Cp^iPr5^)_2_] metallocene compounds occupy the top position, with *U*_eff_ = 2652, 2217 and 2347 K, respectively, and *T*_H_ = 100 K for dysprosium amide-alkene compound and 80 K for metallocene-based compounds, which are already above the temperature of liquid nitrogen.^10–12^ However, such organometallic compounds are always air sensitive, and inert conditions are required for their isolation and study. Another important family of Dy^III^-based SMMs includes complexes with pentagonal-bipyramidal geometry. In this case, a very large variety of *U*_eff_ values ranging from 16 to 1262 cm^–1^ and *T*_B_ up to 36 K is observed.^13–19^ As a rule, complexes containing alkoxide/siloxide ligands in the axial position(s) and neutral ligands in the equatorial plane demonstrate the best performance. Interestingly, their higher coordination numbers compared with metallocenes and simpler synthetic routes make them a potential target for studying related compounds with subtle modifications in the coordination sphere. For example, Zheng and coauthors recently studied a remarkable family of pentagonal-bipyramidal SMMs and identified key parameters that determine the relaxation dynamics.^13^

Nonetheless, many of these complexes are also air sensitive, which can be a drawback for future processing/application in a device. Alternatively, atmospheric conditions and solvents containing donor atoms, such as alcohols/phenols, are often used, resulting in air-stable compounds. However, the large ionic radii of lanthanides usually lead to high coordination numbers (typically larger than 7) due to solvent/water coordination, making the rational design of the target system difficult. Considering that crystal field (CF) parameters are closely related to the arrangement, charge, and number of ligands, their large number, in turn, means a diversity of magnetic properties. This diversity opens up a huge playground for synthetic chemists and provides a great scope for designing or subtly modifying coordination environments. In this regard, even for [Dy­(Cp*)_2_]^+^ metallocene SMMs, variations have been studied, either by changing the substituents in the ring or by changing the nature of some of the atoms forming the ring.^20,21^ These studies are essential to understanding how specific parameters affect the magnetic relaxation behavior in similar systems.

When dealing with complexes with a large coordination number, more positions come into play that can potentially be modified. In this context, Murugesu and co-workers were among the pioneers in modifying the coordination environment of a well-studied complex^22^ while maintaining or even exploiting axial anisotropy.^23^ Based on the dinuclear Dy_2_ system with the general formula [Dy_2_(valdien)_2_(NO_3_)_2_] (H_2_valdien = N1,N3-bis­(3-methoxysalicylidene) diethylenetriamine), where the phenoxido groups are responsible for providing the appropriate ligand field, they managed to modify the nitrate in the equatorial plane (perpendicular to the phenoxido groups) with other chelates providing the system with higher or lower electron density without altering the geometry around the lanthanide. As expected, the lower electron density in this plane, the higher the effective energy barrier that prevents the reorientation of the magnetization.^18^

Inspired by their work,^22,23^ we considered as a prototype our recently published zero-field SMM with the formula [Zn_2_(μ-H_2_L)_2_(μ-succinate)­Dy_2_(NO_3_)_2_]­(NO_3_)_2_·2H_2_O·6MeOH (H_4_L = 1,4,8,11-tetraaza-1,4,8,11-tetrakis­(2-hydroxy-3-methoxy-5-methylbenzyl) cyclotetradecane), hereafter referred **6**.^24^ Its appropriate crystal field, defined by three phenoxido groups in nearly opposite positions of the DyO_9_ coordination sphere, two on one side and one on the other, provides the axial ground state. In the equatorial ligand field (perpendicular to the direction defined by the phenoxido groups at both sides of the Dy^III^ ion), in addition to the methoxy groups of the main ligand, one nitrate and one carboxylate oxygen atom from the bridging succinate are coordinated to the Dy^III^ ion. It has been observed that when strongly electronegative atoms are located in the apical positions of the structure, ligands occupying the equatorial plane contribute to the appearance of transverse components that activate sub-barrier relaxation processes.^25–27^

In this work, we succeeded in modifying the equatorial ligand field in complex **6** by (i) replacing the nitrate with other chelating ligands, (ii) replacing the bridging succinate with tetrafluorosuccinate, or (iii) simultaneously modifying both positions. Thus, herein we report the synthesis, characterization, magnetic and photoluminescence studies, and *ab initio* calculations of five novel complexes based on the H_4_L ligand.

## Results and Discussion

As previously shown,^24^ the ground state of compound **6** is a Kramers doublet with the highest *M*_J_ value (±15/2, eas*y*-axis anisotropy), which is well separated from the excited states. As a result, compound **6** is a zero-field SMM with a relatively high energy barrier (261 K). As mentioned above, we have retained the core structure of **6**, preserving the ligand field provided by the phenoxido groups but slightly changing the electron density in the equatorial plane. This led to the formation of five novel compounds with the general formula: [Zn_2_(μ-H_2_L)_2_(μ-dicarb)­Dy_2_(L’)_2_(L’’)_2_]­X_2_·xH_2_O·yMeOH·zEtOH, where dicarb = tetrafluorosuccinate (**1** and **3**), succinate (**2**, **4** and **5**); L’ = NO_3_^–^ (**1**), hfac (**2** and **3**), CF_3_CO_2_^–^/tfac (**4**), dbm (**5**); L’’ = MeOH (**4**); X = NO_3_^–^ (**1**), OTf (**2**–**5**) and *x*,*y*,*z* = 0,8.5,0 (**1**), 8,2,0 (**2**), 0,0,5.5 (**3**) 0,6.25,0 (**4**) and 2,7,0 (**5**). Powder X-ray diffractograms (Figures S2–S6) confirmed the phase purity of the samples.

### Crystal Structures

Specific information on space groups, crystallographic data, and a summary of the most important bond lengths and angles are given in the ESI (Tables S1–S9). All compounds have the same core structure, as shown in Figure 1. Each double cationic fragment [Zn_2_(μ-H_2_L)_2_(μ-dicarb)­Dy_2_(L’)_2_(L’’)_2_]^2+^ consists of two octadentate (N_2_O_6_) H_2_L^2–^ ligands, two Zn^II^ and two Dy^III^ ions, a bridging succinate (or tetrafluorosuccinate), and two terminal chelates, each coordinated to a Dy^III^ ion. Compound **4**, on the other hand, contains CF_3_CO_2_^–^ molecules that are monocoordinated, so that the methanol molecules complete the DyO_9_ coordination sphere. As for counterions, compounds **1** and **6** contain nitrates for charge balancing, while compounds **2**–**5** contain triflates. In all cases, although the ligand has three deprotonated phenol groups, the overall charge is H_2_L^2–^, since N4 is in the zwitterionic form (Figure S1 shows compound **1**, and the other compounds follow the same label pattern). It is worth noting that in compounds **1**, **3**, **4**, and **5**, the entire molecule is contained in an asymmetric unit cell, in **2** there are three molecules, and in **6** there is only half a molecule.

**Figure 1:**
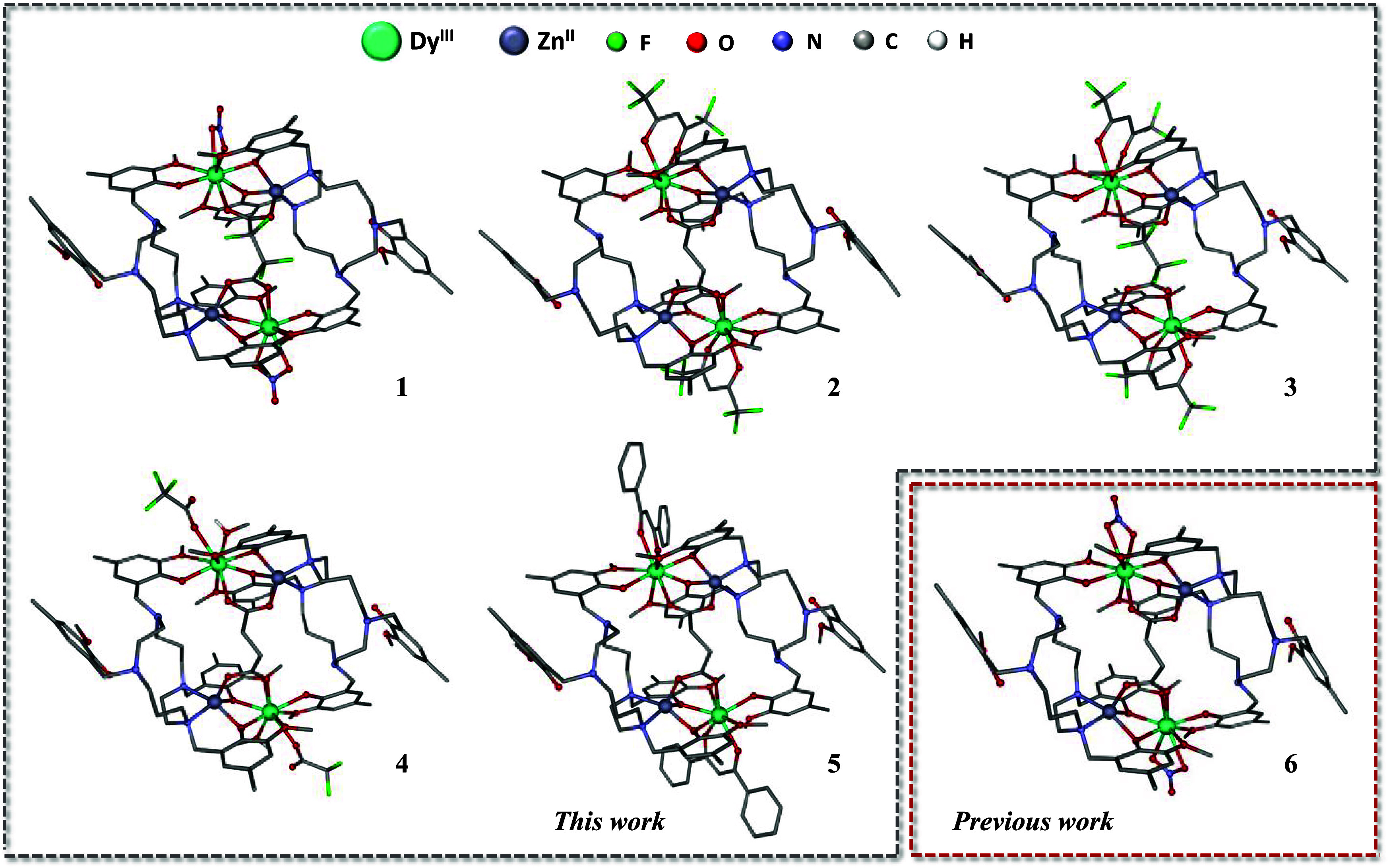
Perspective view of the cationic structures of **1–6**. For clarity, all hydrogen atoms, except for the H atoms of the OH group of methanol in **4**, and counterions have been omitted.

One of the main objectives in the synthesis of these bis­(ZnDy) derivatives was to minimize changes in the coordination sphere in terms of polyhedra but significantly change the electron density distribution around the Dy^III^ ions. The geometries of the coordination environment around the metal ions were evaluated using the SHAPE software.^28^ In the case of Zn^II^ ions, no noticeable differences between the complexes are observed. In all compounds, the ZnN_2_O_3_ sphere fits best to a square pyramid, albeit slightly distorted (Table S10). The case with Dy^III^ is not so simple, since, as expected for the DyO_9_ sphere, there are many more variants. Indeed, among the 13 possible geometric configurations of such a chromophore, none of them is suitable for correctly describing polyhedra formed by donor oxygen atoms. This confirms the difficulty of designing a specific ligand field due to the high coordination numbers of lanthanide ions.^29^ All CShM values exceed 1.615 (the value for Dy1A in **2** for a spherical capped square antiprism, Table S11) for any of the possible ideal polyhedra, confirming a high degree of distortion. However, in summary, it can be stated that all Dy^III^ centers are distorted polyhedra lying between a spherical capped square antiprism, a tricapped trigonal prism, a spherical tricapped trigonal prism, and a muffin. Note that in **5**, Dy1B can be considered intermediate between DyO_9_ and DyO_8_ due to the large Dy–O_methoxy_ distance of 2.921(4) Å (fragment B of the compound), so both geometries were considered (Tables S11–S12).

However, we believe that the ground state axiality created by the phenoxido groups is preserved in almost all complexes, and the main differences are in the chelating and bridging succinate derivatives. Figure 2 demonstrates more clearly the differences in the DyO_9_ coordination sphere. As can be seen, the most electron-donating phenoxido groups are nearly in opposite positions. The equatorial plane, in contrast, consists of methoxy groups, a chelate (nitrate in Figure 2), and a bridging succinate ligand connecting two Zn^II^Dy^III^ units. As mentioned above, the ligand field generated by phenoxido groups is preserved since for all complexes the Dy–O7_phenoxido_ bonds are the shortest (2.227(4)–2.278(3) Å). These bonds are followed by second-third-shorter Dy–O1_phenoxido_ and Dy–O3_phenoxido_ bonds with lengths ranging from 2.263(7) to 2.355(3) Å. The only exception is **5** with the dbm chelate, for which shorter lengths (2.276(3)–2.314(3) Å) are observed, which should negatively affect its behavior as an SMM. For the remaining compounds, chelates (or CF_3_CO_2_^–^ and MeOH in **4**) in the equatorial plane display longer Dy–O bond distances (2.358(3)–2.519(4) Å). Note that compounds containing tetrafluorosuccinate instead of succinate have longer Dy–O_carboxylate_ bonds (Tables S3–S9), which was expected when electron-withdrawing groups were introduced into the molecule.

**Figure 2:**
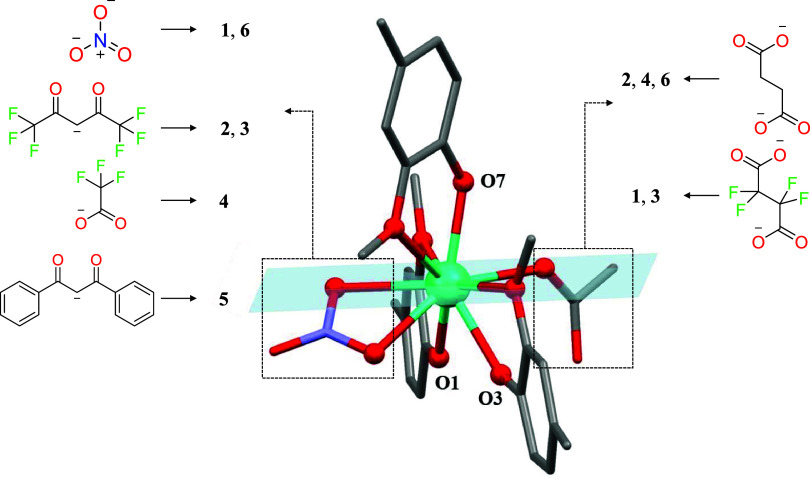
Perspective view of the reduced fragment of **6**. Atoms O7, O1, and O3 provide axiality to Dy^III^; the blue plane indicates the equatorial ligand field; and dashed squares indicate chelating and bridging fragments that have been modified.

As for the most significant bond angles, the core structure is conserved in all the complexes, so that the angles of O1–Dy–O7, O3–Dy–O7, O_carboxylate_–Dy-O7, O_chel1_–Dy-O7 and O_chel2_–Dy-O7 fall within relatively narrow angle ranges: 136.02(12)–141.1(2)°, 133.7(3)–144.16(14)°, 72.43(11)–77.94(13)°, 77.9(2)–100.99(14)° and 123.75(13)–137.49(9)°, respectively.

### Static Magnetic Properties

The temperature dependence of the magnetic susceptibility was measured for polycrystalline samples of complexes 1–5 in the 2–300 K temperature range under an applied field of 0.1 T (Figures S7–S12). At room temperature, the χ_M_ *T* values of 28.52 (**1**), 28.10 (**2**), 28.02 (**3**), 28.75 (**4**), and 27.44 (**5**) cm^3^·mol^–1^·K are within the expected range for the two noninteracting Dy^III^ ions (14.17 cm^3^·mol^–1^·K in the free ion approximation, ^6^H_15/2_, and *g* = 4/3). All complexes display similar behavior with a gradual decrease of the χ_M_ *T* product with decreasing temperature due to depopulation of the *M*_J_ sublevels. In the low-temperature region, all compounds exhibit a more marked final drop, especially pronounced in the case of **1**, which may indicate magnetic blocking. The magnetic anisotropy is confirmed by the low magnetization value (∼10 μ_B_) at the maximum applied magnetic field of 7 T and by the fact that the *M­(H/T)* isotherms virtually superimpose suggesting that the energy separation between the ground and first excited Kramers doublets may be very large for these compounds (Figures S13–S24).

### Dynamic Magnetic Properties

Since the ligand field generated by the phenoxido groups was preserved, zero-field SMM behavior would be expected for most of the new compounds (**1**–**4**). In the case of **5**, however, a dbm chelate was introduced to break the axiality of the ground state and provoke faster relaxation to also validate our concept. Indeed, *ac* measurements carried out at zero *dc* field with an oscillating field of 3.5 Oe revealed out-of-phase maxima for all systems (Figure 3a–d) except **5** (Figure S37). Compounds **1**–**4** exhibit maxima in the χ_M_^’’^(ν) plots below 33, 18, 26, and 22 K, respectively, indicating that the different electronic distribution around the Dy^III^ ion modulates the difference in the properties of the *M*_J_ states (Figures S25–S36). Furthermore, compounds **1**–**4** exhibited long tails below the maxima, indicating an unquenched quantum tunneling of magnetization (QTM).

**Figure 3:**
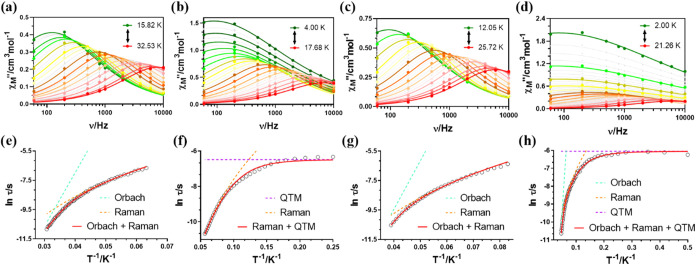
Variable-temperature frequency dependence of the χ_M_^’’^ signal at zero applied field for **1**–**4** (a–d). Arrhenius plots for relaxation times and their fitting took into account the simultaneous presence of several relaxation pathways (e–h).

Figure 3e–h demonstrates the nonlinearity of the *lnτ vs* 1/*T* plots across the entire temperature range. Thus, these temperature dependences were mainly fitted taking into account three relaxation mechanisms operating at zero *dc* field, namely, QTM, Raman (or spin–phonon), and Orbach. In this case, the most commonly used equation for fitting is as follows

1 $$\tau^{-1}=\tau_{\mathrm{QTM}}^{-1}+CT^{n}+\tau_{0,\mathrm{Orbach}}^{-1}\exp\left(-U_{\mathrm{Orbach}}/k_{B}T\right)$$

However, over the past decade, significant progress has been made in the theory of spin-phonon relaxation and a general expression was obtained to describe this relaxation in magnetic molecules.^30,31^ Since usually only the phonons in a small energy window contribute to relaxation, they can be treated as an effective mode with energy ω, so a simplified expression can be used,^31^ which can be further simplified if $\exp\left(\frac{\omega }{k_{B}T}\right)\gg 1$

$$\tau_{s-\mathrm{ph}}^{-1}=V\frac{\exp\left(\frac{\omega }{k_{B}T}\right)}{{\left(\exp\left(\frac{\omega }{k_{B}T}\right)-1\right)}^{2}}\approx V\cdot \exp\left(-\frac{\omega }{k_{B}T}\right)$$

Therefore, to fit the experimental dependences presented in Figure 3e–h, we also used the following formula in eq 2

2 $$\tau^{-1}=\tau_{\mathrm{QTM}}^{-1}+\tau_{0,\mathrm{Raman}}^{-1}\exp\left(-\omega_{\mathrm{Raman}}/k_{B}T\right)+\tau_{0,\mathrm{Orbach}}^{-1}\exp\left(-U_{\mathrm{Orbach}}/k_{B}T\right)$$

For compound **1**, the relaxation times were determined from fitting the χ_M_^’’^(ν) curves in the temperature range 15.82–32.53 K (Figure 3a). The plot of *lnτ vs* 1/*T* dependence shows a clear curvature over almost the entire temperature range, with a small linear segment at the highest temperatures, indicating a combination of different relaxation mechanisms operating simultaneously (Figure 3e). A linear fitting of relaxation times in the Arrhenius plot at the highest temperature region yielded *U*_Orbach_ = 273 K and τ_0_ = 5·10^–9^ s. The fitting using eq 1, disregarding QTM, resulted in the following parameters: *U*_Orbach_ = 370 K, τ_0,Orbach_ = 4·10^–10^ s, *C* = 3·10^–3^ s^–1^ K^–4.5^, and *n* = 4.5 (Table 1). As expected, the linear fitting at high temperatures significantly underestimates the barrier and overestimates the value of τ_0,Orbach_.

**Table 1:** Best-Fit Parameters *(*τ_QTM_^fit^, *C, n*, τ_0,Raman_, ω_Raman_, τ_0,Orbach_, and *U*_Orbach_) Obtained from the Approximation of the Temperature Dependences of Relaxation Times for Compounds **1** and **2** Using eqs 1 and 2 ^a^

	compound **1**			compound **2**			
	Orbach	eq 1	eq 2	Orbach	eq 1	eq 2	eq 2’
τ_QTM_^fit^, s					(1.5 ± 0.2)**·**10^–3^	(0.86 ± 0.08)**·**10^–3^	(4.2 ± 5.5)**·**10^–3^
τ_QTM_^calc^, s					1.5**·**10^–6^ ÷ 1.1**·**10^–3^		
*C*, s^–1^ K^–n^		(3 ± 5) **·**10^–3^			(7.3 ± 1.3)·10^–3^		
*n*		4.5 ± 0.5			5.43 ± 0.06		
τ_0,Raman_, s			(6 ± 3)**·**10^–6^			(1.84 ± 0.12)**·**10^–7^	(1.22 ± 0.12)·10^–7^
							(1.3 ± 0.6)·10^–4^
ω_Raman_, K (in cm^–1^)			89 ± 11 (62 ± 8)			86 ± 1 (59.8 ± 0.7)	94 ± 2 (65 ± 1)
							15 ± 6 (10 ± 4)
τ_0,Orbach_, s	5**·**10^–9^	(4 ± 3)**·**10^–10^	(7 ± 4)**·**10^–10^	2**·**10^–7^			
*U*_Orbach_, K	273	370 ± 30	342 ± 18	82			

a Parameters τ_QTM_^calc^ were estimated theoretically as described in the SI (Section 6b).

Figure 3e also displays the contributions of Raman and Orbach mechanisms to the overall relaxation process; it can be seen that the Raman process operates over a wide range, while the Orbach contribution is limited to the highest temperatures. This is consistent with the anomalously high α values obtained from the Cole–Cole plots (Figure S27), which are abnormally high even at the highest temperatures (0.32­(15.82 K)–0.19­(32.53 K)), where lower values are expected with the dominant contribution of Orbach relaxation. The high α values obtained in the low temperatures (below 20 K) are probably due to the influence of weak QTM. However, no tendency to reach a plateau is observed (Figure 3e), so the QTM relaxation process was not included in the eq 1.

We also performed the fitting of the temperature dependence of the relaxation times for **1** using eq 2 (Figure S45a). Table 1 shows that the best-fit value for the effective phonon mode frequency is 62 ± 8 cm^–1^ (89 ± 11 K), which is consistent with the conclusion that Raman relaxation is significantly affected by the low-energy phonons.^32^ Note that the best-fit parameters for the Orbach mechanism are close to those obtained using eq 1, which validates the approach of spin-phonon relaxation provided by eq 2.

Compound **2** displays maxima at much lower temperatures (Figure 3b) compared to those of **1**, and the temperature dependence of the relaxation time for **2** (Figure 3f) also shows a different pattern. At high and intermediate temperatures, a temperature dependent regime is observed, while at the lowest temperatures, a tendency toward a temperature-independent regime is evident, indicating a significant contribution from QTM. The presence of the latter at low temperatures is also evidenced by a minor shift of the maxima on the χ_M_^’’^ (ν) curves (Figure 3b) and high α values at these low temperatures (0.50­(4.00 K)–0.17­(17.68 K)). The linear Arrhenius fit at the highest temperatures gives *U*_Orbach_ = 82 K and τ_0_ = 2·10^–7^ s, but this energy barrier is much smaller than the calculated energy difference (247 K) between the ground and first excited Kramers doublets (*vide infra*), and in fact it is close to the effective phonon frequency obtained for compound **1**. Thus, in the case of compound **2**, even at high temperatures, it seems that it is not the Orbach but the Raman mechanism that is active. The data were fitted to eq 1 neglecting the Orbach mechanism, which gave τ_QTM_ = 1.5·10^–3^ s, *C* = 7.3·10^–3^ s^–1^ K^–5.43^, *n* = 5.43. When fitting the data to eq 2, τ_QTM_ = 0.86·10^–3^ s, τ_0,Raman_ = 1.84·10^–7^, and ω_Raman_ = 86 K were obtained. Unfortunately, this fitting shows a noticeable discrepancy with the experiment at low temperatures (Figure S45b). However, taking into account the second effective spin-phonon relaxation mode corrected the situation (Table 1, eq 2′, Figure S45c). Overall, it can be concluded that the contribution of the Orbach mechanism to **2** is negligible.

The two modifications implemented in **1** and **2** have been combined in **3**, where the bridging succinate was replaced with tetrafluorosuccinate, and the chelating groups are hfac instead of nitrate ligands. Interestingly, this system exhibits a well-defined set of maxima on the χ_M_^’’^ (ν) plots below 26 K, which is between the values observed for **1** and **2** and is very similar to that for **6** (Figure S40). The Arrhenius temperature dependence of the relaxation time for **3** exhibits a trend very similar to that for **1**, with noticeable curvature over the entire temperature range and a linear section at the highest temperatures. Linear fitting to the Arrhenius law gives values of *U*_Orbach_ and τ_0_ of 190 K and 2·10^–8^ s, respectively (Table 2). Considering the simultaneous presence of the Raman and Orbach processes, fitting according to eq 1 gave: *U*_Orbach_ = 329 K, τ_0,Orbach_ = 1.5·10^–10^ s, *C* = 3.3·10^–3^ s^–1^ K^–4.8^ and *n* = 4.8, while the fitting according to eq 2 yielded: *U*_Orbach_ = 284 K, τ_0,Orbach_ = 6.2·10^–10^ s, ω_Raman_ = 76 K, τ_0,Raman_ = 4.5·10^–6^ s. As for **1**, the Raman process for **3** dominates in the widest range, as can be seen in Figure 3g. It should be noted that the effective mode active in the spin-phonon relaxation of **3** has a frequency that is only slightly lower than in the case of compounds **1** and **2 (** Table 1).

**Table 2:** Best-Fit Parameters *(*τ_QTM_^fit^ *C, n*, τ_0,Raman_, ω_Raman_, τ_0,Orbach_, *U*_Orbach_) Obtained from the Approximation of the Temperature Dependences of Relaxation Times for Compounds **3**, **4**, and **6** Using eqs 1 and 2 ^a^

	compound **3**			compound **4**			compound **6**		
	Orbach	eq 1	eq 2	Orbach	eq 1	eq 2	Orbach	eq 1	eq 2
τ_QTM_^fit^, s					(2.4 ± 0.3)·10^–3^	(1.92 ± 0.17)·10^–3^			
τ_QTM_^calc^, s					4.1·10^–3^				
*C*, s^–1^ K^–n^		(3.3 ± 1.1)·10^–3^			0.38 ± 0.14			(2.8 ± 2.8)·10^–3^	
*n*		4.8 ± 0.1			3.51 ± 0.14			4.7 ± 0.3	
τ_0,Raman_, s			(4.5 ± 0.8)·10^–6^			(1.13 ± 0.16)·10^–5^			(4.9 ± 1.8)·10^–6^
ω_Raman_, K (in cm^–1^)			76 ± 3 (53 ± 2)			44 ± 2 (30 ± 1)			80 ± 7 (56 ± 5)
τ_0,Orbach_, s	2**·**10^–8^	(1.5 ± 0.6)·10^–10^	(6.2 ± 1.7)·10^–10^	6**·**10^–8^	(8 ± 2)·10^–10^	(2.0 ± 0.4)·10^–9^	6·10^–9^	(2.4 ± 1.5)·10^–10^	(5.4 ± 2.2)·10^–10^
*U*_Orbach_, K	190	329 ± 11	284 ± 8	130	232 ± 7	208 ± 5	226	330 ± 19	302 ± 12

a Parameter τ_QTM_^calc^ was estimated theoretically as described in SI (Section 6b).

Compound **4** exhibits maxima in the χ_M_^’’^(ν) dependences below 22 K (Figure 3d). In this case, we were able to fit the curves from the lowest temperature (2.0 K) to 21.2 K (Figure 3d), which allowed us to obtain more information about the tunneling regime. The Arrhenius plot of the relaxation times in Figure 3h shows a linear part at the highest temperatures due to the Orbach process, a curvature associated with the Raman mechanism in the middle range, and a region of temperature-independent τ values at the lowest temperatures due to quantum tunneling of magnetization. Linear fitting in the highest temperatures gave *U*_Orbach_ = 130 K and τ_0_ = 6·10^–8^ s. The simultaneous presence of different mechanisms is reflected in the high α values obtained from the Cole–Cole plots (0.64­(2.00 K)–0.20 (21.26 K)). Fitting the curve over the entire temperature range, taking into account the QTM, Raman, and Orbach mechanisms using eq 1, yielded the following parameters: τ_QTM_ = 2.4·10^–3^ s, *C* = 3.8·10^–1^ s^–1^ K^–3.51^, *n* = 3.51, and *U*_Orbach_ = 232 K, τ_0,Orbach_ = 8·10^–10^ s, and when using eq 2: τ_QTM_ = 1.9·10^–3^ s, ω_Raman_ = 44 K, τ_0,Raman_ = 1.1·10^–5^ s, *U*_Orbach_ = 208 K, τ_0,Orbach_ = 2.0·10^–9^ s (Table 2).

As mentioned above, compound **5** does not exhibit any maxima in the out-of-phase frequency dependence in the absence of an external magnetic field. This was expected, given the short bonds between the metal center and the oxygen atoms of the dbm chelate as well as the electron-donating ability of the latter. In order to at least partially suppress QTM, measurements were performed in an external magnetic field of 1 kOe (Figure S37). In the temperature dependence of the in-phase component of the ac susceptibility, a maximum was recorded near 2 K, while for the out-of-phase component, no maximum was detected; most likely, it manifests itself below the temperature limit of the device.

The data for compound **6** can be found in our previous paper,^24^ where only linear fitting was performed at the highest temperatures (well-defined maxima in the χ_M_^’’^(ν) plots are below 28 K) yielding *U*_Orbach_ = 261.0 K, τ_0_ = 2.1·10^–9^ s. However, the data was fitted again for this work extending the studied temperature range (13.22–27.63 K). Importantly, the new results allowed us to consider the Raman contribution besides the Orbach. Thus, the fitting to eq 1 provided: *C* = 2.8·10^–3^ s^–1^ K^–4.7^, *n* = 4.7 and *U*_Orbach_ = 330 K, τ_0,Orbach_ = 2.4·10^–10^ s, and when using eq 2: ω_Raman_ = 80 K, τ_0,Raman_ = 4.9·10^–6^ s, *U*_Orbach_ = 302 K, τ_0,Orbach_ = 5.4·10^–10^ s (Figures S40–S42). It is worth mentioning that the best-fit parameters of **6** are close to those of **3**.

In addition, alternative fits on a logarithmic scale were performed for compounds **1**–**4** and **6** (SI, Figures S47–S57), and the best-fit parameters are given in Tables S13–S15. Overall, the parameters obtained do not differ significantly from those given in Tables 1 and 2, although the *U*_Orbach_ values are systematically lower. However, it is important that the trend of parameter changes with modification of the coordination sphere does not alter, and the compounds under study can be arranged in a series according to SMM performance: **1** > **3** ≈ **6** > **4** ≫ **2**.

### Hysteretic Behavior

In view of the zero-field SMM behavior of compounds **1**–**4**, magnetization hysteresis loop measurements were performed at 2 K on powder samples. Figure 4 displays the hysteresis loop of compound **1**, which shows the largest opening at 2 K. The Supporting Information (Figures S58–S61) provides comparative plots for **1** and other complexes **2**–**6**. All compounds exhibit butterfly shaped hysteresis loops without any remnant magnetization at zero field. This is consistent with a significant contribution of the QTM regime, which also manifests itself in the long tail χ_M_^’’^(*T*) plots at the lowest temperatures. The loop opening is consistent with relaxation times (Figure S44) and *U*_Orbach_ values, which were obtained considering only the Orbach process, implying that the compounds can be sorted by performance as **1** > **3** ∼ **6** > **4** > **2**.

**Figure 4:**
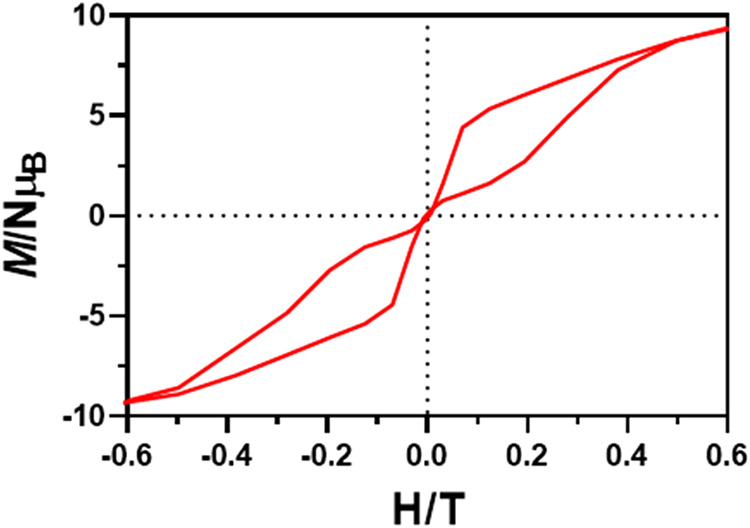
Magnetic hysteresis loop for **1** at 2 K; the employed sweep-rates were slower at low fields, with an average value of 61 Oe/s.

### Theoretical Calculations

To clarify the observed magnetic behavior, *ab initio* calculations were performed for all compounds at the SA-CASSCF­(9,7)/SO_RASSI level^33–36^ using the OpenMolcas software.^37^ Static magnetic properties were calculated using the SINGLE_ANISO procedure.^38^ Due to the large number of atoms in each tetranuclear complex, representative dinuclear fragments preserving the core structure (named **1′**–**6′**) were chosen. For this purpose, as shown in Figures S62–S67, we proceeded as follows: (i) a cationic fragment (with +1 charge) was selected for each compound; (ii) a random Zn^II^Dy^III^ fragment was considered; and (iii) half of the bridging ligand (one-half bound to both inner and outer pockets and the other half in which one phenol derivative acts as monodentate and the other one is uncoordinated) was selected for each fragment. As can be seen in the figures mentioned, the main axes of magnetic anisotropy are mostly influenced by the short Dy–O7_phenoxido_ bonds.

Table S16 summarizes the results of calculations for eight Kramers’ doublets (KDs) of **1’**. As can be seen, the ground state (KD1) is strongly anisotropic with nearly perfect axiality (*g*_*z*_ = 19.9, *g*_*x*_ = 0.0004; *g*_*y*_ = 0.001) and is almost a pure state with *M*_J_ = ± 15/2 (99%). An axial character is also observed for the first excited state (KD2), while the second excited state (KD3) displays minor transverse *g*-tensor components and is a mixture of two states with different *M*_J_. Moreover, matrix elements of the transient dipole moment governing the QTM in the ground state, as well as the TA-QTM through the first excited state, exhibit low values (2·10^–4^ and 2·10^–2^ μ_B_, respectively; Figure 5, dashed black lines) in agreement with the observed zero-field SMM behavior.

**Figure 5:**
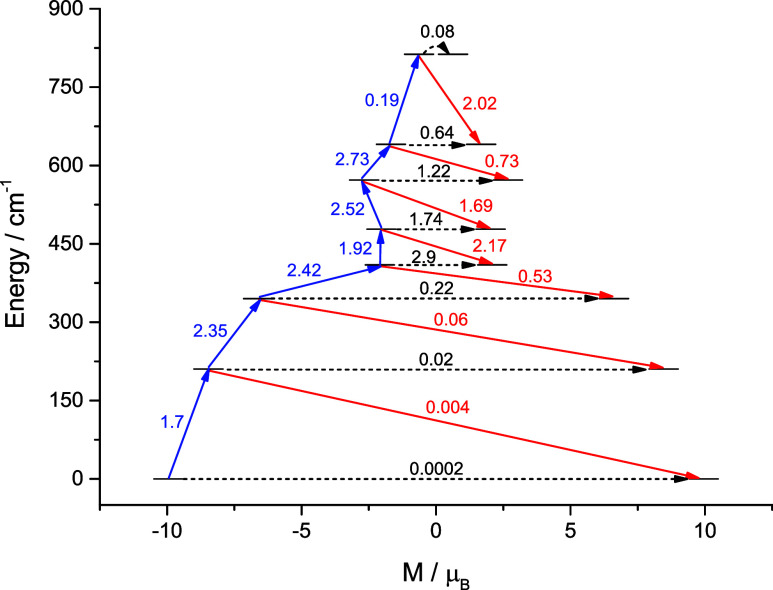
Magnetization blocking barrier for **1’**. The exchange states are arranged according to the values of their magnetic moments. The arrows show the connected exchange states, and the numbers at each of them stand for the corresponding matrix elements of the transient magnetic moment.

If we consider magnetization relaxation solely as thermally assisted tunneling due to spin–spin dipolar interaction and neglect other relaxation mechanisms, then the values of *U*_eff_ and τ_QTM_ can be estimated using the approach proposed by D. Aravena^39,40^ (hereinafter referred to as the DA model, SI, Section 6b). In this case, the relaxation in complex **1** should efficiently proceed through KD3–KD5 with ***U***_**eff**_^**calc**^ ≈ 640 K. In fact, other relaxation mechanisms are also important, such as spin-phonon coupling,^41^ which leads to much lower experimental values: 370 and 342 K, depending on the Raman relaxation treatment. Both values lie between the energies of KD2 and KD3 (302 and 496 K, respectively), indicating relaxation via these excited KDs (although mainly via KD2). Very similar results were recently obtained in our previous work for compound **6**, as well as for a bent bis­(amide) dysprosium^42^ and dysprosium SMM with two axial spiropyran ligands.^43^

The results of calculations for **2’** are summarized in Table S17. As in the case of **1’**, the ground and first excited states are almost pure *M*_J_ = ± 15/2 and ± 13/2, respectively. However, both have larger transverse components of the *g*-tensor compared to **1**, although the calculations predict (Figure S68) a very low matrix element of the transient dipole moment (3·10^–3^ μ_B_) for the ground state, which agrees well with the zero-field SMM behavior. Note that the DA model predicts relaxation predominantly through KD3 with ***U***_**eff**_^**calc**^ ≈ 420 K. However, experimentally estimated *U*_Orbach_ = 82 K turned out to be significantly lower than even the energy of the first excited KD (247 K). For this reason, in the previous section, magnetization relaxation was attributed to a combination of Raman and QTM mechanisms, ruling out the possibility of relaxation via the Orbach process. Moreover, the effective energy of phonons leading to relaxation of magnetization is close for all studied compounds (85 ± 9 K), except for **4** (44 K).

As mentioned above, unlike compounds **1** and **3**–**6**, the unit cell of **2** contains three molecules. The DA model allowed us to calculate the τ_QTM_ values for each independent Dy^III^ ion (Table S24), which lie in a very wide range (1.5·10^–6^–1.1·10^–3^ s) for **2**, and, therefore, each type of ion will contribute differently to magnetization relaxation. The largest calculated value of τ_QTM_ = 1.1·10^–3^ s is in good agreement with the experimentally obtained values (0.9–1.5)·10^–3^ s. Most likely, only a subset of dysprosium complexes with a sufficiently slow QTM exhibit SMM properties.

According to calculations (Table S18), the ground and first excited states of **3′** are axial with small transverse *g*-tensor components, corresponding to almost pure wave functions with *M*_J_ = ± 15/2 and ± 13/2, respectively. The calculations also predict (Figure S69) low matrix elements of the transient magnetic moment in both the ground and first excited states and a much larger value in KD3 (0.7 μ_B_). The calculations using the DA model predict a contribution of KD3–KD5 to the demagnetization of **3** with ***U***_**eff**_^**calc**^ ≈ 517 K. In contrast, the experimental value of *U*_Orbach_ = 329 and 284 K, depending on the Raman relaxation treatment, is close to the KD2 energy (285 K).

In compound **4′** (Table S19), as in all the previous cases, the ground state is almost perfectly axial with negligibly small transverse components of the *g*-tensor. In the first excited state (KD2), the contribution of the component with *M*_J_ = ± 13/2 is insufficient (∼85%) although *g*_*x*_ and *g*_*y*_ components are quite small (Figure S70). The energy of KD2 (198 K) is also close to the experimental value of *U*_Orbach_ = 220 ± 12 K. In contrast, calculations using the DA model predict the participation of KD2–KD4 in the relaxation with ***U***_**eff**_^**calc**^ ≈ 348 K. It should be noted that for compound **4** we were able to experimentally estimate the value of τ_QTM_ = (1.92–2.4)·10^–3^ s, which turned out to be very close to the value calculated using the DA model (4.1·10^–3^ s). This, in turn, confirms that the dipole–dipole interaction between the selected Dy^III^ cation and the surrounding Dy^III^ centers is the predominant source of quantum tunneling.

As follows from the crystallographic details and from the *ac* magnetic measurements, the properties of **5** differ significantly from those of the other studied compounds. The theoretical results for **5′** are also significantly different and help to rationalize the lack of zero-field SMM behavior for **5**. Although its KD1 is quite axial (contribution of *M*_J_ = ± 15/2 is 91.5%), the *g*_*x*_ and *g*_*y*_ values for **5′** in this state are noticeably larger than those for **1′**–**4′** and **6′** (Tables S16–S21). Moreover, the excited KD2 and KD3 have much lower energies, and their wave functions are described by a combination of several components with different *M*_J_. At the same time, already in the ground state, the matrix element of the transient magnetic moment is rather large (0.14 μ_B_, Figure S71), and the predicted τ_QTM_ is very small (∼3·10^–7^ s), which explains the absence of the zero-field SMM behavior.

### Magnetostructural Correlations

The analysis of both experimental and theoretical results allows us to establish an important magnetostructural correlation. Comparison of the results for **1** and **6**, which differ only in the bridging ligand (tetrafluorosuccinate or succinate, respectively), shows that the original goal of removing electron density from the equatorial plane has been achieved. Indeed, the Dy-OXS bond length (OXS is the oxygen atom of succinate/tetrafluorosuccinate) in **6** is 2.320(4), whereas in **1** it is longer (2.377(4)–2.432(4) Å), leading to the better performance of **1**. Moreover, the oxygen atoms in the coordination sphere of the Dy^III^ cation of tetrafluorosuccinate-containing compounds have lower Mulliken charges (Table 3), which was our goal when choosing the fluorine-substituted ligands that withdraw electron density.

**Table 3:** Results of Calculations at the SA-CASSCF­(9,7) Level of the Mulliken Atomic Charges of Atoms Coordinated to Dy^III^ in Complexes **1**–**6**

comp.	Mulliken atomic charges								
	axial ligands			equatorial ligands					
	O7	O1	O3	O1S	O1_chel_	O2_chel_	O2	O4	O8
**1**	–1.145	–1.131	–1.131	–0.975	–0.692	–0.680	–0.812	–0.837	–0.827
**2**	–1.159	–1.138	–1.110	–1.000	–0.917	–0.928	–0.800	–0.840	–0.823
**3**	–1.160	–1.141	–1.109	–0.959	–0.926	–0.915	–0.800	–0.833	–0.813
**4**	–1.125	–1.147	–1.138	–0.990	–0.898	–1.001	–0.817	–0.827	–0.805
**5**	–1.176	–1.115	–1.086	–1.013	–1.001	–0.998	–0.785	–0.810	–0.769
**6**	–1.142	–1.126	–1.129	–1.002	–0.686	–0.690	–0.809	–0.840	–0.823

It is noteworthy that the behavior of **2** turned out to be unexpected. Based on previous work by Murugesu and co-workers,^23^ it was expected that replacing nitrate chelates with hfac groups would have a positive effect on SMM behavior. The authors claimed that the fluorinated chelate provides lower electron density in the equatorial plane of the metal cation, thereby improving the SMM behavior. However, **2** exhibits noticeably shorter Dy–O_chelate_ bond lengths compared to **6**, which are in the ranges 2.364(8)–2.432(7) Å and 2.456(5)–2.519(4) Å, respectively. In addition, it was found that the calculated Mulliken charges for hfac are significantly more negative than those for nitrate. Thus, although not initially predicted, the results obtained help to understand why **2** has worse SMM properties. In addition, compound **2** demonstrated several unexpected differences from compounds **1**, **3**, **4**, and **6**. First, **2** exhibits very efficient Raman relaxation with a value of 1/τ_0,Raman_ = 5.4 × 10^6^ s^–1^, while for other compounds this value is more than an order of magnitude lower (0.9 ÷ 2.2) × 10^5^ s^–1^. Second, **2** differs from others in that it contains not one molecule in the crystal cell, as in most of the compounds studied, but three. Moreover, the calculated times of QTM in the ground state of the six types of Dy^III^ centers differ by 3 orders of magnitude. It is practically impossible to predict such an effect of replacing one ligand on the crystal structure as a whole.

The properties observed for **3** can be explained by the sum of the opposite effects considered for **1** and **2**. Indeed, the crystal structure of **3** shows long Dy-OXS bonds (2.437(2)–2.440(2) Å) and shorter Dy–O_chelate_ bonds (2.358(3)–2.416(2) Å). Thus, the positions of the maxima on the χ_M_^’’^(ν) plots appear reasonable, since they lie between those for **1** and **2**, and are also similar to the positions in the case of **6**.

Compound **4** does not quite correspond to what was intended. Trifluoroacetate was supposed to be used as a chelate, but it acted as a monocoordinating ligand, while the methanol molecule occupied the other position. Thus, in this case, dissimilar effects manifested themselves. On the one hand, the Dy–O_CF3CO2_ bond lengths are close to those observed for hfac, and the negative charge is more concentrated in one position due to the monocoordinating character of the trifluoroacetate. The predicted Mulliken charge for it is −1.001, which is significantly greater in absolute value than that for the oxygen atoms of hfac. This could negatively affect SMM behavior, but the neutral nature of the coordinated methanol molecule has a positive effect on the SMM properties. Overall, considering both effects, the observed intermediate SMM behavior becomes clear.

Finally, the results obtained for complex **5** are also easy to rationalize. On the one hand, as we have already mentioned, dbm chelating groups have much shorter coordination bonds, and since they are located in the equatorial plane, this negatively affects the ground state axiality. On the other hand, these diketones do not have such strong electron-withdrawing groups as the fluorinated ligands hfac or tfac, and thus they have the largest negative Mulliken charge on the coordinating O atoms of all the chelating groups used. Therefore, the lack of SMM behavior in zero-field conditions for **5** is obvious.

### Photoluminescence Properties

For compound **6**, it was previously shown that the H_4_L ligand is able to sensitize the emission of the Dy^III^ ion via the well-known antenna effect. Indeed, a broad band originating from the n → π* and π → π* transitions of H_4_L, peaking at 300 nm, was observed in the excitation spectrum of **6**. This spectrum was recorded by monitoring the emission line at 578 nm, which is often the most intense Dy^III^ emission band associated with the ^4^F_9/2_ → ^6^H_13/2_ transition. This inspired us to study the photoluminescent properties of compounds **1**–**5** as well. Excitation spectra were recorded for all studied compounds at room and cryogenic temperatures (Figures S79–S83). It was found that for compounds **1** and **4**, ligand-centered transitions with broad bands with maxima at about 300 nm dominate the excitation spectra. These bands are followed by less intense and narrow f-f transitions, which indicate a more efficient antenna effect compared with **6**, where f-f transitions dominate the excitation spectrum. In contrast, compounds **2** and **3**, containing hfac chelates, exhibit less intense ligand-centered transitions, and their excitation spectra are dominated by f-f transitions (Figures S80–S81), indicating a weak antenna effect. Compound **5** differs from the others in its remarkable temperature dependence, which is discussed in the SI (Figure S83 and accompanying text).

The behavior observed in the excitation spectra is reproduced in the emission spectra. For complexes **1** and **4**, the Dy^III^ emission bands dominate the spectrum at room temperature (monochromatic laser excitation with λ_ex_ = 325 nm, Figures S84 and 6 for **1** and **4**, respectively). More specifically, the ^4^F_9/2_ → ^6^H_15/2_ and ^4^F_9/2_ → ^6^H_13/2_ transitions (blue and yellow emission lines) are the most intense, with a larger contribution from the yellow line, consistent with the low symmetry of the coordination spheres around the metal ions.^44^ In addition, weak ligand fluorescence occurs in the form of a broad band in the 350–550 nm range. As anticipated from the excitation spectra, the room temperature emissions of **2** and **3** are almost completely dominated by ligand fluorescence, although the narrow ^4^F_9/2_ → ^6^H_13/2_ transition arises against a broad background band. Finally, in the case of compound **5** at room temperature, the emissions originating from H_4_L (350–550 nm range), dbm (peaking at 445 nm), and Dy^III^ (482 and 578 nm) coexist without any dominant contribution.

**Figure 6:**
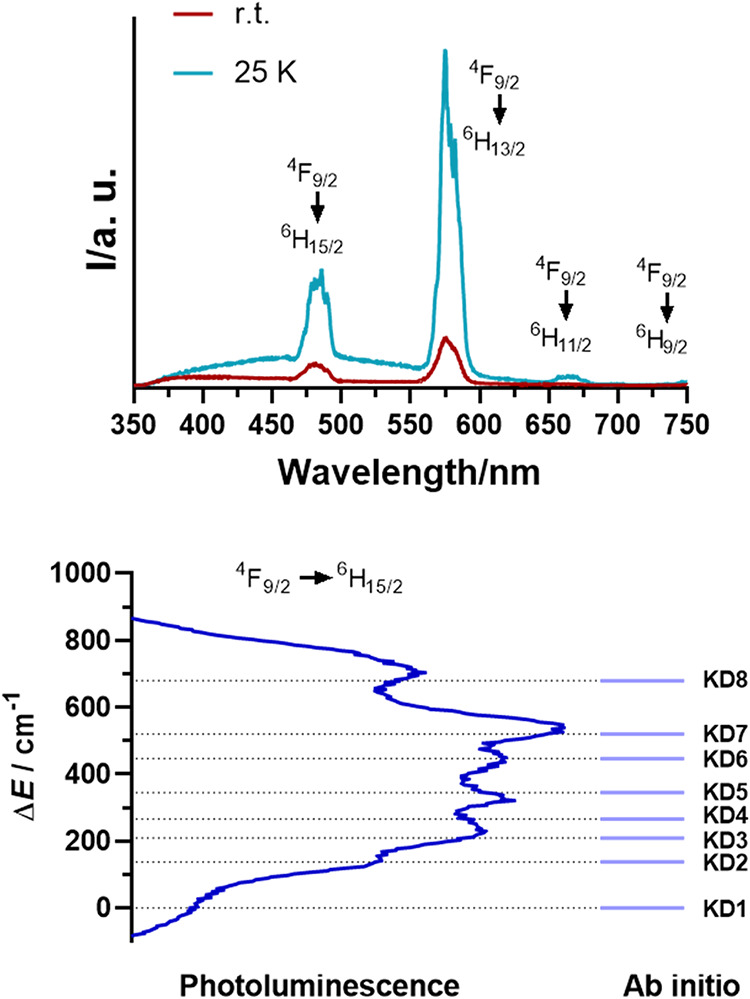
Solid-state photoluminescence emission spectrum of compound **4** recorded at 25 K and room temperature (top). The ^4^F_9/2_ → ^6^H_15/2_ emission band of compound **4** was recorded at 25 K on a relative energy scale (bottom left) and the calculated *ab initio* relative energies of the Kramers doublets (bottom right).

The weakness and short duration of the emission of these compounds in most cases did not allow us to estimate their lifetimes, although for compounds **1**, **4**, and **5**, it was possible to analyze the Dy^III^-centered emission, characterized by two main components (Table 4). As can be seen, under laser irradiation, the ligand is capable of efficiently transferring energy to Dy^III^, resulting in relatively intense emission of 16–19 μs. On the other hand, it can also be argued that the emission of the dbm ligand in **5** is short-lived fluorescence, as expected.

The analysis of the lifetimes at low temperature allows us to obtain some important conclusions about the photoluminescence of these compounds. First, the Dy^III^-centered photoluminescence is weakly affected by the temperature, since the lifetimes are practically temperature-independent (only available for compounds **1**, **4**, and **5**). Second, the ligand-centered emission has a significantly shorter lifetime than the Dy^III^-centered one (by about 3 orders of magnitude) and does not vary much with coligands, remaining in the 0.5–3 ns range. Finally, it is worth highlighting that the ligand-centered emission becomes shorter with a decrease in the temperature, which is particularly visible for compound **5**. This is most likely due to an increase in the antenna effect, manifested by an increase in the Dy^III^-centered emission, as shown for compound **4** in Figure 6. Note that a deeper analysis of the photoluminescent properties can be found in the SI, including the low-temperature spectra and thermometric features of some samples.

**Table 4:** Photophysical Properties of Compounds **1**–**5**

	^a^	^b^		^b^	τ_obs_ ^c^	
comp.	λ_ex_ ^a^	ex. comp.^b^	λ_em_	em. comp.^b^	RT	LT
**1**	305	ligand	576	Dy^III^	19.1 μs	15.8 μs
**2**	340	ligand	545	ligand	–	1.2 ns
	399	Dy^II^	576	Dy^III^	–	10.6 μs
**3**	340	ligand	544	ligand	–	2.7 ns
	328	ligand	576	Dy^III^	–	48.3 μs
	386	Dy^III^	576	Dy^III^	–	17.5 μs
**4**	340	ligand	400	ligand	–	0.50 ns
	305	ligand	576	Dy^III^	17.5 μs	14.5 μs
**5**	340	ligand	450	ligand	2.0 ns	1.6 ns
	305	ligand	576	Dy^III^	16.2 μs	16.5 μs

a Note that an excitation wavelength of 340 nm corresponds to the wavelength of the pulsed LED source. Other wavelengths were tuned using a pulsed lamp and modulable laser to adjust to the excitation band maxima.

b The excitation/emission composition, i.e., the type of excitation/emission that differs between Dy-centered and ligand-centered.

c Note that some data could only be recorded at low temperature because the RT signal was too weak.

Using the same approach as that for **6** in our previous work, we were able to estimate the relative energies of the KDs arising from the ground ^6^H_15/2_ term. For this purpose, we focused on the cryogenic temperature emission centered at 482 nm since it covers a series of transitions from the lower KD of the excited term ^4^F_9/2_ to eight KDs of the ground term. It is important to note that (i) low-temperature emission spectra were used since at higher temperatures transitions from excited KDs contaminate the emission spectrum; (ii) only compounds **1**, **4** and **5** were studied in detail since in **2** and **3** individual transitions were not identified due to weak emission. As an example, Figure 6 shows an analysis of the spectrum of compound **4** (for **1** and **5**, see Figures S104–S105). The experimentally observed energy difference between the low-energy shoulder at 473.4 nm and the last maximum of the emission spectrum at 489.5 nm is 695 cm^–1^, which corresponds to the energy difference of KD1 and KD8. This value agrees well with the energy difference of 680 cm^–1^ calculated *ab initio* (Figure 6, bottom). Moreover, individual transitions in KD2, KD3..., and KD7 also agree well with theoretical calculations, confirming their high accuracy.

## Conclusions

In this work, five new bis­(ZnDy) complexes were synthesized, and their structures, magnetic properties, and photoluminescence were studied and analyzed using *ab initio* calculations. All compounds display similar axial environments defined by three phenoxido donors, while systematic modifications in the equatorial ligand field modulate the electron density around Dy^III^ and result in markedly different SMM behavior.

The electron-withdrawing equatorial ligands tetrafluorosuccinate and nitrate form longer Dy–O bonds and reduce the electron density on the coordinating oxygen atoms, stabilizing the ground Kramers doublet *M*_J_ = ± 15/2 and affording improved SMM performance (e.g., *U*_Orbach_ = 370 ± 30 K for compound **1**, compared to 330 ± 19 K for prototype **6**). More electron-donating ligands compress the coordination sphere and reduce anisotropy, resulting in weakening or disappearance of SMM behavior in zero-field, as observed for complex **5** containing a dbm ligand. Combinations of ligands with opposite effects (e.g., tfac and MeOH for **4**) produce magnetic responses corresponding to the balance of their electronic effects.

However, SMM properties can be influenced not only by the molecular structure but also by the crystal packing as a whole. This is clearly seen for compound **2**, which is characterized by six types of Dy^III^-centers in the crystal cell, rather than two, as in most of the compounds studied. The calculated magnetization tunneling times in the ground Kramers doublets of these centers differ by almost 3 orders of magnitude due to significant differences in dipolar interactions with surrounding Dy ions.

Dy^III^-centered emission was observed for all compounds studied; structured bands at 482 nm allowed us to estimate the splitting of the ground terms ^6^H_15/2_ for them. The relative energies of the Kramers doublets obtained from spectroscopy are in excellent agreement with those predicted in *ab initio* calculations, which confirm the high accuracy of the calculations and reinforce the established correlation among structure, magnetic anisotropy, and theoretical calculations.

## Experimental Section

### Synthetic Procedures

#### General Procedures

All reactions were conducted in oven-dried glassware under aerobic conditions. All analytical grade reagents were purchased from commercial sources and used without further purification. H_4_L and compound **6** were synthesized following the procedure previously reported by us.^24^

#### Synthesis of [Zn_2_(μ-H_2_L)_2_(μ-F_4_suc)­Dy_2_(NO_3_)_2_]­(NO_3_)_2_·8.5MeOH (**1**)

To a solution of H_4_L (0.05 mmol, 40.0 mg), Zn­(NO_3_)_2_·6H_2_O (0.05 mmol, 14.9 mg) and Dy­(NO_3_)·5H_2_O (0.05 mmol, 21.9 mg) in 4 mL of methanol was added dropwise another solution containing tetrafluorosuccinic acid (F_4_suc; 0.025 mmol, 4.8 mg) and NaOH (0.15 mmol from a methanolic 0.5 M solution) in 0.6 mL of methanol. A fine powder was formed after adding each drop, which easily dissolved. The solution was stirred for a few seconds, filtered in order to remove possible undissolved particles, and allowed to stand at room temperature. In a few days suitable crystals for X-ray diffraction were obtained. Yield: 57%.

#### Synthesis of [Zn_2_(μ-H_2_L)_2_(μ-suc)­Dy_2_(hfac)_2_]­(OTf)_2_·8H_2_O·2MeOH (**2**)

To a solution of H_4_L (0.05 mmol, 40.0 mg), Zn­(CF_3_COCHCOCF_3_)_2_·2H_2_O (hfac; 0.05 mmol, 25.8 mg) and Dy­(CF_3_SO_3_)_3_ (OTf; 0.05 mmol, 30.5 mg) in 10 mL of methanol was added dropwise another solution containing succinic acid (suc; 0.025 mmol, 3.0 mg) and NaOH (0.15 mmol from a methanolic 0.5 M solution) in 0.6 mL of methanol. A fine powder was formed in each drop, which easily got dissolved. The pale yellow solution was stirred for a few seconds, filtered in order to remove any possible insoluble material, and allowed to stand at room temperature. In a few days suitable yellow crystals for X-ray diffraction were obtained. Yield: 44%.

#### Synthesis of [Zn_2_(μ-H_2_L)_2_(μ-F_4_suc)­Dy_2_(hfac)_2_]­(OTf)_2_·5.5EtOH (**3**)

To a solution of H_4_L (0.05 mmol, 40.0 mg), Zn­(CF_3_COCHCOCF_3_)_2_·2H_2_O (0.05 mmol, 25.8 mg) and Dy­(CF_3_SO_3_)_3_ (0.05 mmol, 30.5 mg) in 14 mL of ethanol was added dropwise another solution containing tetrafluorosuccinic acid (0.025 mmol, 4.8 mg) and Et_3_N (0.15 mmol, 0.020 mL) in 1 mL of ethanol. A fine powder was formed in each drop, which it easily got dissolved. The pale yellow solution was stirred for a few seconds, filtered in order to remove possible undissolved particles, and allowed to stand at room temperature. In a few days suitable yellow crystals for X-ray diffraction were obtained. Yield: 46%.

#### Synthesis of [Zn_2_(μ-H_2_L)_2_(μ-suc)­Dy_2_(CF_3_CO_2_)_2_(MeOH)_2_]­(OTf)_2_·6.25MeOH (**4**)

To a solution of H_4_L (0.075 mmol, 60.0 mg), Zn­(CF_3_CO_2_)_2_·xH_2_O (0.075 mmol, 21.9 mg) and Dy­(CF_3_SO_3_)_3_ (0.075 mmol, 45.7 mg) in 20 mL of methanol was added dropwise another solution containing succinic acid (0.0375 mmol, 4.4 mg) and Et_3_N (0.225 mmol, 0.030 mL) in 1 mL of methanol. In view of the high solubility of the product, the solution was further concentrated by heating it at 75 °C until a considerable amount of solid was obtained, and then the solid was filtered while hot. Suitable crystals for X-ray diffraction were achieved from a diluted reaction. It is noteworthy that a second phase similar to this compound, **4b**, was also obtained in the same reaction, which contains a water molecule coordinated to the Dy^III^ ion instead of a methanol molecule (Figure S1, bottom). However, as shown in PXRD experiments, the phase that was studied corresponds to pure **4**. Yield: 38%.

#### Synthesis of [Zn_2_(μ-H_2_L)_2_(μ-suc)­Dy_2_(dbm)_2_]­(OTf)_2_·2H_2_O·7MeOH (**5**)

To a solution of H_4_L (0.02 mmol, 16.0 mg), Zn­(OTf)_2_·(0.02 mmol, 7.3 mg), Dy­(CF_3_SO_3_)_3_ (0.02 mmol, 12.2 mg) and 1,3-Diphenyl-1,3-propanedione (dbm; 0.02 mmol, 4.5 mg) in 20 mL of methanol was added dropwise another solution containing succinic acid (0.01 mmol, 1.2 mg) and Et_3_N (0.08 mmol, 0.011 mL) in 1 mL of methanol. The colorless solution was stirred for a few seconds, filtered in order to remove any possible insoluble material, and allowed to stand at room temperature. In a few days suitable crystals for X-ray diffraction were obtained. Yield: 30%.

### Physical Measurements

Magnetic susceptibility measurements were carried out on polycrystalline samples of the complexes with a Quantum Design SQUID MPMS-7T magnetometer at an applied magnetic field of 1000 G. The susceptibility data were corrected for the diamagnetism estimated from Pascal’s Tables,^45^ the temperature-independent paramagnetism, and the magnetization of the sample holder. Alternating current measurements were performed on a Quantum Design PPMS-6000 magnetometer under a 3.5 G ac field and frequencies ranging from 60 to 10000 Hz.

### X-ray Diffraction Data Collection and Structure Determination

Suitable single crystals of compounds **1**, **2**, and **5** were mounted on a Bruker D8 VENTURE diffractometer equipped with an area detector and graphite monochromated Mo Kα radiation (λ = 0.71073 Å). Compounds **3**, **4**, and **4b** were mounted on an Agilent Technologies Super Nova diffractometer with monochromatic Mo Kα radiation (λ = 0.71073 Å) and an Atlas CCD detector. Data collection was performed by applying the ω-scan method used for the structure determination at 100 K. Data reduction was performed with the APEX2 ^46^ software, and absorption was corrected using SADABS^47^ for compounds **1**, **2**, and **5**; and CrysAlisPro software and ABSPACK^48^ were used for compounds **3**, **4**, and **4b**. Crystal structures were solved by intrinsic phasing using the SHELXT program^49^ and refined by full-matrix least-squares on *F* ^2^ including all reflections employing the WINGX crystallographic package^50,51^ for compounds **2** and **4b** and Olex2 software^52^ for compounds **1**, **3**, **4**, and **5**. The main crystallographic details and refinement data can be found in Table S1. During the refinement process of compounds **2**, **3**, **4**, and **4b** the solvent molecules were clearly visible but were not assigned due to the large disorder. Therefore, the structures were resolved with a squeeze routine, assigning the residual electron density to the solvent molecules. In the cases of **1** and **5**, however, a reasonable model was obtained with the mentioned crystallization molecules. Crystallographic data have been deposited with the Cambridge Crystallographic Data Centre as supplementary publication with no. CCDC 2409934–2409939.

The X-ray powder diffraction (XRPD) patterns were measured on preground single crystals (Figures S2–S6). For data acquisition, a Philips X’PERT powder diffractometer was used with Cu–Kα radiation (λ = 1.5418 Å) over the range 5 < 2θ < 50° with a step size of 0.026° and a data acquisition time of 2.5 s per step at 25 °C.

### Computational Methodology

In the case of complexes **1**–**5**, the tetranuclear bis­(ZnDy) clusters have rather large intracluster Dy–Dy distances (8.7–9.0 Å). Therefore, for calculations, we divided such clusters into two ZnDy binuclear units by breaking five C–C bonds, adding five hydrogen atoms to one unit, and discarding the other. Binuclear complexes are called **1′**–**5′**. The geometries of this complex obtained from crystallographic determination were used in calculations without further relaxation.

For all *ab initio* electronic structure calculations discussed in this paper, the OpenMolcas program^37^ (version 2019-11) was used. The electronic energies and wave functions of spin multiplets (21 sextets, 128 quartets, and 130 doublets) were calculated at the state-averaged^33^ (SA) CASSCF­(9,7) level^34,53^ (active space: nine electrons distributed over the seven f-orbitals of Dy). Scalar relativistic effects were taken into account using the DKH2 Hamiltonian.^35,54,55^ The relativistic ANO-RCC-VTZP basis set for Dy and O atoms of coordination sphere and the smaller ANO-RCC-VDZ basis set for other atoms were used in the calculations.^56^ The spin–orbit coupling was treated nonperturbatively within the mean-field theory in the restricted active space state interaction (SO-RASSI) method,^36,57^ in which the CASSCF wave functions are used as the basis states. Diagonalization of the spin–orbit matrix leads to spin–orbit multiplets, e.g., Kramers doublets in the case of Dy^III^ complexes.

To calculate parameters of the effective spin (pseudospin) Hamiltonians (*g*-tensors, their principal values, angular momenta along the principal magnetic axes, matrix elements of the magnetic moment, etc.) and the static magnetic properties of complexes **1′**–**5′**, the SINGLE_ANISO program was used.^38,58^

In addition, using the approach proposed by Aravena^39^ and the “UandTau” program developed in his group,^40^ we have estimated for compounds **1**–**6** the tunneling relaxation times (τ_QT_) in the eight lower Kramers doublets as well as the effective demagnetization barriers (*U*_eff_).

In the applied model, the source of quantum tunneling is spin–spin dipolar interaction: the selected Dy^III^ cation with pseudospin 1/2 interacts with a number of Dy centers belonging to the environment. The influence of distant magnetic centers is incorporated by repeating the unit cell in all directions until energy broadening was converged. For **1**–6, the total number of Dy centers required to achieve convergence varied between 1372 (for **6**) and 85750 (for **2**). As input information, the program used the positions of unique Dy centers in the unit cells of compounds **1**–**6**, the Kramers doublet energies corresponding to the ^6^H_15/2_ term, the *g*-tensor components, and *g*-vectors obtained in SO-RASSI/SINGLE-ANISO calculations. To obtain reliable results, the *g*-vectors were reoriented according to the position of each unique metal center.

The spin-flip matrix elements of the Hamiltonian describing the dipolar interaction are further converted to the energies used in the Fermi golden rule to calculate the relaxation rates. The relaxation contributions of each Kramers doublet as a function of temperature were calculated using the relaxation times, τ_QT_ = 1/2*k*_QT_, the temperature dependence of relaxation rate according to the Boltzmann law, $k_{i}\left(T\right)\propto \frac{\exp\left(-E_{i}/k_{B}T\right)}{Z}k_{\mathrm{QT},i}$ , and the weighted contribution of relaxation rates to the effective demagnetization barrier, $U_{\mathrm{eff}}\left(T\right)=\sum_{i=1}^{M}\frac{k_{i}\left(T\right)}{N_{k}}E_{i}$ , where *N*_*k*_ is the normalization factor of *k*_*i*_(*T*).

### Photoluminescence Measurements

Fluorescence excitation and emission spectra and lifetime measurements on solid state were recorded on an Edinburgh Instruments FL980 spectrometer at variable temperature using a closed-cycle helium cryostat enclosed in the spectrometer. For steady-state measurements, a Müller-Elektronik-Optik SVX1450 Xe arc lamp or a Kimmon IK3552R-G He–Cd continuous laser (325 nm) was used as the excitation source. Instead, the decay curves were measured with a microsecond pulsed μF900 lamp, a PicoQuant 340 nm LED or an Ekspla OPO NT342B laser system according to the signal liveness and intensity. For the variable temperature measurements, the samples were first placed under a high vacuum (of ca. 10^–8^ mbar) to avoid the presence of oxygen or water in the sample holder. Photographs of irradiated single-crystals or polycrystalline samples were taken at room temperature in a micro-PL system included in an Olympus optical microscope illuminated with a Hg lamp.

## Supplementary Material



## Notes

Crystallographic tables, continuous shape measurements, additional figures, experimental XRPD, magnetic measurements, *ab initio* calculations, photoluminescence properties, references (PDF)
