# Supplementary material for: Fine-Tuning the SMM Properties of a Bis(ZnDy)-Based Compound by Rationally Reducing/Increasing the Electron Density in the Equatorial Plane Perpendicular to the Ophenoxido–Dy‑Ophenoxido‑bridges Direction
Source: Inorg Chem. 2026 Apr 23;65(18):10348–61. doi: 10.1021/acs.inorgchem.6c01258 (PMC13169370; doi:10.1021/acs.inorgchem.6c01258)
Supplement: Supplementary file 1 [file ic6c01258_si_001.pdf]

Supporting Information

Fine-tuning the SMM properties of a bis(ZnDy) based compound by rationally reducing/increasing the electron density in the equatorial plane perpendicular to the $O_{phenoxido}$-Dy-$O_{phenoxido\text{-}bridges}$ direction

Andoni Zabala-Lekuona,*[a] Javier Cepeda,[a] José A. García,[b] Nina P. Gritsan,*[c] Alexey A. Dmitriev,[c] Pablo Salcedo-Abraira,[d] Antonio Rodríguez-Diéguez,[d] José M. Seco*[a] and Enrique Colacio*[d]

[a]*Departamento de Química Aplicada, Facultad de Química, Universidad del País Vasco (UPV/EHU), 20018 Donostia-San Sebastián, Spain. Email:* andoni.zabala@ehu.eus; josemanuel.seco@ehu.eus

[b]*Departamento de Física, Facultad de Ciencia y Tecnología, Universidad del País Vasco (UPV/EHU), 48940 Leioa, Spain.*

[c]*Institute of Chemical Kinetics and Combustion, Siberian Branch, Russian Academy of Sciences, 630090 Novosibirsk, Russia. Email:* gritsan@kinetics.nsc.ru

[d]*Departamento de Química Inorgánica, Facultad de Ciencias, Universidad de Granada, 18071 Granada, Spain. Email:* ecolacio@ugr.es

Index:

S1. **Crystallographic Tables.**

S2. **Continuous Shape Measurements.**

S3. **Additional Figures.**

S4. **Experimental XRPD.**

S5. **Magnetic Measurements.**

S6. ***Ab initio* calculations.**

S7. **Photoluminescence properties.**

S8. **References**

S1. Crystallographic Tables.

Table S1.- Crystallographic data for compounds **1-3**.

**Compound**	**1**	**2**	**3**
Formula	$C_{104.5}H_{157}N_{12}O_{40.5}F_4Zn_2Dy_2$	$C_{110}H_{154}N_8O_{40}F_{18}S_2Zn_2Dy_2$	$C_{119}H_{159}N_8O_{35.5}F_{22}S_2Zn_2Dy_2$
CCDC number	2409938	2409939	2409936
M_r	2761.15	3090.26	3207.39
Crystal system	*monoclinic*	*triclinic*	*triclinic*
Space group (no.)	*P* 21/c (14)	*P* -1 (2)	*P* -1 (2)
a (Å)	13.0698(8)	19.2020(3)	17.0854(3)
b (Å)	48.732(3)	27.6848(5)	18.9558(3)
c (Å)	20.0222(12)	39.2013(8)	23.3790(5)
α (°)	90	89.466(2)	97.183(2)
β (°)	105.084(2)	78.420(2)	93.196(2)
γ (°)	90	75.048(2)	114.960(2)
V (Å^3)	12313.2(13)	19704.3(7)	6760.9(2)
Z	4	6	2
D_c (g cm^{-3})	1.489	1.563	1.576
μ(MoK$_\alpha$) (mm^{-1})	1.676	1.624	1.582
T (K)	100.00(10)	100(2)	100.01(10)
Observed reflections	21729 (18168)	73436 (44480)	26734 (21516)
R_{int}	0.0643	0.0515	0.0220
Parameters	1535	4207	1467
GOF	1.153	1.023	1.010
$R_1^{a,b}$	0.0730 (0.0571)	0.1336 (0.0845)	0.0494 (0.0365)
wR_2^{c}	0.1276 (0.1218)	0.2329 (0.1960)	0.0899 (0.0834)
Largest difference in peak and hole (e Å^{-3})	2.028 and -2.032	5.858 and -3.043	1.353 and -0.856

$^{a}R_1 = \Sigma||F_o| - |F_c||/\Sigma|F_o|$. ^{b}Values in parentheses for reflections with $I > 2\sigma(I)$. $^{c}wR_2 = \{\Sigma[w(F_o^2 - F_c^2)^2]/\Sigma[w(F_o^2)^2]\}^{½}$.

Table S2.- Crystallographic data for compounds **4**, **4b**, **5** and **6**.

**Compound**	**4**	**4b**	**5**	**6**[1]
Formula	$C_{110.25}H_{161}N_8O_{38.25}F_{12}S_2Zn_2Dy_2$	$C_{108}H_{156}N_8O_{38}F_{12}S_2Zn_2Dy_2$	$C_{135}H_{182}N_8O_{39}F_6S_2Zn_2Dy_2$	$C_{102}H_{156}N_{12}O_{40}Zn_2Dy_2$
CCDC number	2409937	2409935	2409934	2226683
M_r	2958.32	2922.26	3174.74	2646.12
Crystal system	*triclinic*	*monoclinic*	*triclinic*	*orthorhombic*
Space group (no.)	*P* -1 (2)	*C* 2/*c* (15)	*P* -1 (2)	*Pba*2 (32)
a (Å)	12.252(2)	35.7147(9)	16.9088(9)	20.89300(10)
b (Å)	18.4676(10)	12.1364(2)	19.4639(11)	22.74300(10)
c (Å)	30.4352(19)	32.9862(7)	23.3378(13)	12.2250(8)
α (°)	73.731(5)	90	100.267(2)	90
β (°)	86.145(10)	109.406(3)	95.998(2)	90
γ (°)	88.167(10)	90	106.595(2)	90
V (Å^3)	6595.3(13)	13485.5(5)	7143.9(7)	5808.9(4)
Z	2	4	2	2
D_c (g cm^{-3})	1.490	1.439	1.476	1.513
μ(MoK$_\alpha$) (mm^{-1})	1.606	1.570	1.483	7.970
T (K)	100.00(10)	100.00(10)	100(2)	150.00(10)
Observed reflections	23064 (12319)	15251 (11019)	25130 (20848)	11050 (10714)
R_{int}	0.1014	0.0640	0.0336	0.0609
Parameters	1661	802	1803	659
GOF	0.967	1.051	1.219	1.106
$R_1^{a,b}$	0.1477 (0.0866)	0.1037 (0.0731)	0.0651 (0.0472)	0.0403 (0.0389)
wR_2^c	0.2395 (0.2135)	0.2146 (0.1903)	0.1093 (0.0947)	0.0904 (0.0898)
Largest difference in peak and hole (e Å^{-3})	4.108 and -1.837	2.384 and -1.833	2.264 and -1.694	0.798 and -1.557

$^aR_1 = \Sigma||F_o| - |F_c||/\Sigma|F_o|$. ^{b}Values in parentheses for reflections with $I > 2\sigma(I)$. $^cwR_2 = \{\Sigma[w(F_o^2 - F_c^2)^2]/\Sigma[w(F_o^2)^2]\}^{½}$. ^{d} μ(CuK$_\alpha$) (mm^{-1}) in **6**.

Table S3.- Bond lengths (Å) and angles (°) for compound **1**. Note that when referring to fragment A O7 and O8 belong to ligand B and vice versa. OXS and OYS are O1S and O2S for fragment A and O3S and O4S for fragment B.

**Compound**	**1 (A)**	**1 (B)**
Dy1···Zn1	3.3832(7)	3.4021(7)
Dy1-O1	2.280(4)	2.280(4)
Dy1-O2	2.667(4)	2.584(4)
Dy1-O3	2.316(4)	2.298(4)
Dy1-O4	2.681(4)	2.625(4)
Dy1-O7	2.234(4)	2.237(4)
Dy1-O8	2.630(4)	2.689(4)
Dy1-O1N	2.447(4)	2.427(4)
Dy1-O2N	2.456(4)	2.454(4)
Dy1-OXS	2.377(4)	2.432(4)
Zn1-N1	2.097(5)	2.099(4)
Zn1-N2	2.167(5)	2.169(4)
Zn1-O1	2.075(4)	2.066(4)
Zn1-O3	1.990(4)	1.966(4)
Zn1-OYS	2.021(4)	2.007(4)
Dy1-O1-Zn1	101.83(15)	102.91(15)
Dy1-O3-Zn1	103.32(17)	105.59(17)
O1-Dy1-O3	66.22(14)	66.52(13)
O1-Dy1-O7	138.54(14)	139.06(13)
O1-Dy1-O1N	86.14(14)	80.24(14)
O1-Dy1-O2N	79.39(15)	78.01(13)
O1-Dy1-OXS	81.73(13)	79.22(13)
O3-Dy1-O7	144.16(14)	137.93(15)
O3-Dy1-O1N	127.47(14)	73.66(16)
O3-Dy1-O2N	78.33(14)	119.39(15)
O3-Dy1-OXS	83.00(14)	78.34(14)
O7-Dy1-O1N	85.56(14)	131.76(14)
O7-Dy1-O2N	124.48(14)	100.99(14)
O7-Dy1-OXS	77.94(13)	77.04(13)
O1-Zn1-O3	76.27(16)	77.00(15)
O1-Zn1-OYS	94.63(15)	96.21(15)
O3-Zn1-OYS	104.75(17)	100.92(17)
N1-Zn1-N2	84.7(2)	85.08(17)

Table S4.- Bond lengths (Å) and angles (°) for compound **2**. Note that when referring to fragment A O7 and O8 belong to ligand B and vice versa. OXS and OYS are O1S and O2S for fragment A and O3S and O4S for fragment B.

**Compound**	**2 (A)**	**2 (B)**
Dy1···Zn1	3.3695(12)	3.3699(10)
Dy1-O1	2.263(7)	2.276(5)
Dy1-O2	2.625(10)	2.650(6)
Dy1-O3	2.329(8)	2.321(6)
Dy1-O4	2.679(9)	2.671(6)
Dy1-O7	2.237(6)	2.243(5)
Dy1-O8	2.693(8)	2.646(6)
Dy1-O1H	2.364(8)	2.401(6)
Dy1-O2H	2.398(10)	2.419(6)
Dy1-OXS	2.387(6)	2.374(6)
Zn1-N1	2.112(8)	2.107(7)
Zn1-N2	2.184(9)	2.208(6)
Zn1-O1	2.074(8)	2.113(5)
Zn1-O3	1.975(8)	1.978(6)
Zn1-OYS	1.960(6)	1.963(5)
Dy1-O1-Zn1	101.9(4)	100.3(2)
Dy1-O3-Zn1	102.8(4)	102.9(3)
O1-Dy1-O3	68.2(3)	68.1(2)
O1-Dy1-O7	139.8(3)	139.2(2)
O1-Dy1-O1H	81.8(3)	82.6(2)
O1-Dy1-O2H	82.3(3)	78.2(2)
O1-Dy1-OXS	80.4(2)	80.82(19)
O3-Dy1-O7	133.7(3)	135.5(2)
O3-Dy1-O1H	132.0(3)	133.6(2)
O3-Dy1-O2H	67.7(3)	68.5(2)
O3-Dy1-OXS	76.7(3)	78.3(2)
O7-Dy1-O1H	93.3(3)	90.1(2)
O7-Dy1-O2H	134.2(3)	136.5(2)
O7-Dy1-OXS	75.4(2)	75.24(19)
O1-Zn1-O3	78.9(4)	78.0(2)
O1-Zn1-OYS	97.4(3)	97.5(2)
O3-Zn1-OYS	101.4(3)	105.1(2)
N1-Zn1-N2	83.8(4)	84.8(2)

Table S5.- Bond lengths (Å) and angles (°) for compound **2**. Note that when referring to fragment C O7 and O8 belong to ligand D and vice versa. OXS and OYS are O1S and O2S for fragment C and O3S and O4S for fragment D.

**Compound**	**2 (C)**	**2 (D)**
Dy1···Zn1	3.3749(11)	3.3605(12)
Dy1-O1	2.276(6)	2.283(7)
Dy1-O2	2.644(7)	2.702(8)
Dy1-O3	2.325(6)	2.325(6)
Dy1-O4	2.664(6)	2.669(7)
Dy1-O7	2.235(5)	2.241(5)
Dy1-O8	2.670(6)	2.627(7)
Dy1-O1H	2.381(7)	2.398(7)
Dy1-O2H	2.394(6)	2.425(7)
Dy1-OXS	2.403(6)	2.370(6)
Zn1-N1	2.101(7)	2.098(8)
Zn1-N2	2.171(7)	2.199(7)
Zn1-O1	2.095(6)	2.089(6)
Zn1-O3	1.974(6)	1.975(7)
Zn1-OYS	1.971(6)	1.965(6)
Dy1-O1-Zn1	101.0(2)	100.4(2)
Dy1-O3-Zn1	103.2(3)	102.5(3)
O1-Dy1-O3	68.2(2)	68.0(2)
O1-Dy1-O7	138.9(2)	138.5(3)
O1-Dy1-O1H	81.8(2)	82.6(3)
O1-Dy1-O2H	80.9(2)	78.2(3)
O1-Dy1-OXS	79.7(2)	80.1(2)
O3-Dy1-O7	135.5(2)	137.5(2)
O3-Dy1-O1H	69.8(2)	133.2(2)
O3-Dy1-O2H	132.5(2)	68.8(2)
O3-Dy1-OXS	77.8(2)	79.8(2)
O7-Dy1-O1H	133.6(2)	88.4(2)
O7-Dy1-O2H	91.4(2)	135.8(2)
O7-Dy1-OXS	76.0(2)	75.7(2)
O1-Zn1-O3	78.6(2)	78.6(3)
O1-Zn1-OYS	96.9(2)	95.7(3)
O3-Zn1-OYS	103.8(2)	105.1(3)
N1-Zn1-N2	84.7(3)	84.8(3)

Table S6.- Bond lengths (Å) and angles (°) for compound **2**. Note that when referring to fragment E O7 and O8 belong to ligand F and vice versa. OXS and OYS are O1S and O2S for fragment E and O3S and O4S for fragment F.

**Compound**	**2 (E)**	**2 (F)**
Dy1···Zn1	3.3680(10)	3.3838(13)
Dy1-O1	2.286(6)	2.310(7)
Dy1-O2	2.670(7)	2.671(7
Dy1-O3	2.315(6)	2.292(7)
Dy1-O4	2.709(6)	2.660(7)
Dy1-O7	2.238(5)	2.262(6)
Dy1-O8	2.660(7)	2.684(7)
Dy1-O1H	2.388(6)	2.383(6)
Dy1-O2H	2.432(7)	2.395(7)
Dy1-OXS	2.366(6)	2.365(6)
Zn1-N1	2.100(7)	2.121(8)
Zn1-N2	2.197(7)	2.160(8)
Zn1-O1	2.101(6)	2.095(6)
Zn1-O3	1.983(6)	1.988(7)
Zn1-OYS	1.961(6)	1.960(6)
Dy1-O1-Zn1	100.2(2)	100.2(3)
Dy1-O3-Zn1	102.9(3)	104.3(3)
O1-Dy1-O3	68.3(2)	67.0(2)
O1-Dy1-O7	139.2(2)	141.1(2)
O1-Dy1-O1H	84.8(2)	79.4(2)
O1-Dy1-O2H	77.1(2)	83.8(2)
O1-Dy1-OXS	80.9(2)	82.0(2)
O3-Dy1-O7	135.1(2)	137.0(2)
O3-Dy1-O1H	135.5(2)	71.3(2)
O3-Dy1-O2H	69.4(2)	135.6(2)
O3-Dy1-OXS	78.4(2)	78.0(2)
O7-Dy1-O1H	88.2(2)	131.9(3)
O7-Dy1-O2H	137.1(2)	86.5(2)
O7-Dy1-OXS	75.0(2)	76.9(2)
O1-Zn1-O3	78.4(2)	76.8(3)
O1-Zn1-OYS	97.6(2)	96.7(2)
O3-Zn1-OYS	103.9(2)	104.7(3)
N1-Zn1-N2	84.5(3)	84.5(3)

Table S7.- Bond lengths (Å) and angles (°) for compound **3**. Note that when referring to fragment A O7 and O8 belong to ligand B and vice versa. OXS and OYS are O1S and O2S for fragment A and O3S and O4S for fragment B.

**Compound**	**3 (A)**	**3 (B)**
Dy1···Zn1	3.3990(4)	3.4098(4)
Dy1-O1	2.270(2)	2.291(2)
Dy1-O2	2.667(3)	2.644(3)
Dy1-O3	2.316(2)	2.331(3)
Dy1-O4	2.670(2)	2.658(3)
Dy1-O7	2.238(2)	2.238(2)
Dy1-O8	2.597(3)	2.674(3)
Dy1-O1H	2.387(2)	2.358(3)
Dy1-O2H	2.416(2)	2.377(3)
Dy1-OXS	2.437(2)	2.440(2)
Zn1-N1	2.093(3)	2.103(3)
Zn1-N2	2.163(3)	2.157(3)
Zn1-O1	2.074(2)	2.068(3)
Zn1-O3	1.970(2)	1.961(2)
Zn1-OYS	2.000(2)	1.996(2)
Dy1-O1-Zn1	102.89(9)	102.81(10)
Dy1-O3-Zn1	104.65(9)	104.91(11)
O1-Dy1-O3	67.08(7)	67.13(9)
O1-Dy1-O7	138.24(9)	140.41(9)
O1-Dy1-O1H	81.78(8)	80.09(9)
O1-Dy1-O2H	78.19(9)	81.85(9)
O1-Dy1-OXS	79.39(7)	79.69(8)
O3-Dy1-O7	135.76(8)	134.27(8)
O3-Dy1-O1H	133.15(8)	70.43(9)
O3-Dy1-O2H	69.29(8)	133.89(9)
O3-Dy1-OXS	77.61(7)	75.50(8)
O7-Dy1-O1H	90.72(8)	134.34(9)
O7-Dy1-O2H	137.49(9)	91.29(9)
O7-Dy1-OXS	75.16(8)	76.80(8)
O1-Zn1-O3	77.54(9)	78.70(10)
O1-Zn1-OYS	94.58(9)	95.74(10)
O3-Zn1-OYS	102.70(9)	99.66(10)
N1-Zn1-N2	85.53(10)	84.92(12)

Table S8.- Bond lengths (Å) and angles (°) for compound **4**. Note that when referring to fragment A O7 and O8 belong to ligand B and vice versa. OXS and OYS are O1S and O2S for fragment A and O3S and O4S for fragment B.

Compound	4 (A)	4 (B)
Dy1···Zn1	3.3888(13)	3.3836(13)
Dy1-O1	2.316(6)	2.309(7)
Dy1-O2	2.623(7)	2.674(7)
Dy1-O3	2.314(7)	2.332(7)
Dy1-O4	2.657(7)	2.688(7)
Dy1-O7	2.247(7)	2.269(7)
Dy1-O8	2.772(7)	2.723(8)
Dy1-O1T	2.371(7)	2.373(7)
Dy1-O1M	2.412(7)	2.407(6)
Dy1-OXS	2.354(7)	2.364(6)
Zn1-N1	2.115(8)	2.112(9)
Zn1-N2	2.188(9)	2.183(8)
Zn1-O1	2.102(7)	2.097(7)
Zn1-O3	1.972(7)	1.991(7)
Zn1-OYS	1.973(7)	1.969(7)
Dy1-O1-Zn1	100.1(3)	100.2(3)
Dy1-O3-Zn1	104.2(3)	102.7(3)
O1-Dy1-O3	65.7(2)	66.5(2)
O1-Dy1-O7	139.3(2)	139.2(2)
O1-Dy1-O1T	94.5(2)	95.5(3)
O1-Dy1-O1M	85.5(2)	84.1(2)
O1-Dy1-OXS	81.2(2)	81.4(2)
O3-Dy1-O7	137.8(2)	137.3(2)
O3-Dy1-O1T	141.6(3)	142.6(2)
O3-Dy1-O1M	72.7(2)	73.7(2)
O3-Dy1-OXS	80.5(3)	81.5(2)
O7-Dy1-O1T	78.7(3)	77.9(2)
O7-Dy1-O1M	128.7(2)	129.4(2)
O7-Dy1-OXS	74.2(2)	73.3(2)
O1-Zn1-O3	76.1(3)	76.9(3)
O1-Zn1-OYS	97.5(3)	96.3(3)
O3-Zn1-OYS	107.0(3)	107.0(3)
N1-Zn1-N2	85.3(3)	85.1(3)

Table S9.- Bond lengths (Å) and angles (°) for compounds **5** and **6**. Note that when referring to fragment A O7 and O8 belong to ligand B and vice versa. OXS and OYS are O1S and O2S for fragment A and O3S and O4S for fragment B.

Compound	5 (A)	5 (B)	6
Dy1···Zn1	3.4057(6)	3.4130(6)	3.3611(8)
Dy1-O1	2.323(3)	2.332(3)	2.286(4)
Dy1-O2	2.678(3)	2.667(3)	2.536(5)
Dy1-O3	2.355(3)	2.331(3)	2.313(4)
Dy1-O4	2.648(3)	2.627(3)	2.653(5)
Dy1-O7	2.278(3)	2.263(3)	2.227(4)
Dy1-O8	2.785(3)	2.921(4)	2.708(5)
Dy1-O1C/D	2.306(3)	2.276(3)	2.456(5)
Dy1-O2C/D	2.298(3)	2.314(3)	2.519(4)
Dy1-OXS	2.424(3)	2.432(3)	2.320(4)
Zn1-N1	2.085(4)	2.099(4)	2.101(5)
Zn1-N2	2.231(4)	2.200(4)	2.190(5)
Zn1-O1	2.111(3)	2.109(3)	2.083(4)
Zn1-O3	1.951(3)	1.956(3)	1.995(5)
Zn1-OYS	1.961(3)	1.965(3)	1.979(4)
Dy1-O1-Zn1	100.24(13)	100.33(13)	100.46(17)
Dy1-O3-Zn1	104.15(14)	105.17(14)	102.3(2)
O1-Dy1-O3	65.90(11)	66.08(11)	65.96(15)
O1-Dy1-O7	136.16(12)	136.02(12)	137.32(16)
O1-Dy1-O1C/D	92.45(12)	84.96(12)	83.69(16)
O1-Dy1-O2C/D	85.39(12)	94.60(12)	92.24(15)
O1-Dy1-OXS	79.87(11)	79.32(11)	81.44(15)
O3-Dy1-O7	136.50(12)	135.66(12)	143.73(15)
O3-Dy1-O1C/D	71.45(11)	132.23(12)	78.55(16)
O3-Dy1-O2C/D	132.17(12)	72.87(12)	127.66(15)
O3-Dy1-OXS	78.56(11)	77.44(11)	84.52(16)
O7-Dy1-O1C/D	127.86(12)	91.65(12)	123.75(15)
O7-Dy1-O2C/D	90.79(12)	126.11(12)	84.53(14)
O7-Dy1-OXS	72.43(11)	72.75(11)	75.29(14)
O1-Zn1-O3	77.45(13)	77.34(13)	75.72(17)
O1-Zn1-OYS	97.78(13)	98.55(13)	96.48(18)
O3-Zn1-OYS	107.11(14)	105.59(14)	107.65(18)
N1-Zn1-N2	83.94(15)	84.37(15)	84.3(2)

S2. Continuous Shape Measurements.

The nearer the value to zero, the better fits to an ideal polyhedron.

Table S10.- Continuous Shape Measurements for the ZnN_2O_3 coordination environment in compounds **1-6**.

PP-5	1 D5h	Pentagon
vOC-5	2 C4v	Vacant octahedron
TBPY-5	3 D3h	Trigonal bipyramid
SPY-5	4 C4v	Spherical square pyramid
JTBPY-5	5 D3h	Johnson trigonal bipyramid J12

Structure [ML5]	PP-5	vOC-5	TBPY-5	SPY-5	JTBPY-5
**Zn1A (1)**	30.883	2.069	4.102	**0.826**	6.170
**Zn1B (1)**	30.574	1.943	5.705	**0.624**	7.745
**Zn1A (2)**	30.273	1.936	5.796	**0.564**	8.156
**Zn1B (2)**	30.207	2.008	5.339	**0.544**	7.160
**Zn1C (2)**	29.802	1.916	5.709	**0.512**	7.677
**Zn1D (2)**	30.576	2.110	4.658	**0.623**	6.550
**Zn1E (2)**	30.695	1.988	5.233	**0.513**	7.127
**Zn1F (2)**	30.371	2.073	5.070	**0.581**	7.187
**Zn1A (3)**	30.658	2.035	4.934	**0.645**	6.981
**Zn1B (3)**	30.639	1.877	5.929	**0.596**	8.104
**Zn1A (4)**	29.484	2.070	5.163	**0.628**	7.084
**Zn1B (4)**	29.876	1.989	4.631	**0.644**	6.592
**Zn1A (5)**	29.807	2.032	4.994	**0.668**	6.549
**Zn1B (5)**	29.977	2.000	5.499	**0.560**	7.269
**Zn1 (6)**	29.270	2.116	4.850	**0.724**	6.843

Table S11.- Continuous Shape Measurements for the DyO_9 coordination environment in compounds **1-6**. Note that we have considered a DyO_9 coordination environment also for fragment B in **5**.

EP-9	1 D9h	Enneagon
OPY-9	2 C8v	Octagonal pyramid
HBPY-9	3 D7h	Heptagonal bipyramid
JTC-9	4 C3v	Johnson triangular cupola J3
JCCU-9	5 C4v	Capped cube J8
CCU-9	6 C4v	Spherical-relaxed capped cube
JCSAPR-9	7 C4v	Capped square antiprism J10
CSAPR-9	8 C4v	Spherical capped square antiprism
JTCTPR-9	9 D3h	Tricapped trigonal prism J51
TCTPR-9	10 D3h	Spherical tricapped trigonal prism
JTDIC-9	11 C3v	Tridiminished icosahedron J63
HH-9	12 C2v	Hula-hoop
MFF-9	13 Cs	Muffin

Structure [ML9]	EP-9	OPY-9	HBPY-9	JTC-9	JCCU-9	CCU-9	JCSAPR-9	CSAPR-9	JTCTPR-9	TCTPR-9	JTDIC-9	HH-9	MFF-9
**Dy1A (1)**	35.418	22.342	15.827	15.669	7.227	6.578	2.809	2.466	4.069	3.142	12.606	9.313	**2.258**
**Dy1B (1)**	35.721	21.181	18.504	15.026	9.228	8.363	3.291	**2.002**	3.393	2.396	10.858	9.996	2.239
**Dy1A (2)**	32.801	22.892	19.206	14.458	9.649	8.285	2.799	**1.615**	2.281	2.348	10.820	9.451	2.197
**Dy1B (2)**	34.343	23.519	18.381	15.168	9.519	8.837	2.360	1.800	2.221	**1.745**	11.618	9.374	2.001
**Dy1C (2)**	32.933	23.186	18.463	14.037	9.555	8.666	2.954	**1.728**	2.219	2.179	11.021	9.461	2.226
**Dy1D (2)**	34.463	23.473	17.669	15.047	8.662	8.383	2.043	1.883	2.314	**1.850**	11.822	9.533	1.919
**Dy1E (2)**	34.223	23.371	17.665	15.309	9.447	8.354	2.448	2.199	2.422	**2.022**	11.523	8.651	2.053
**Dy1F (2)**	33.965	23.078	16.642	14.745	8.907	8.109	2.368	2.034	2.267	**1.969**	11.790	9.181	2.015
**Dy1A (3)**	34.725	23.212	18.064	15.228	9.020	8.633	2.187	1.930	2.364	**1.721**	11.830	9.612	1.965
**Dy1B (3)**	33.220	23.181	18.136	14.043	9.074	8.249	2.995	**1.871**	2.160	2.084	11.350	9.306	2.430
**Dy1A (4)**	31.657	22.483	15.048	13.022	8.487	7.865	2.663	2.367	2.481	2.749	11.129	8.003	**2.137**
**Dy1B (4)**	32.391	22.093	14.336	13.533	8.476	7.680	2.657	2.421	2.906	3.024	11.121	7.508	**2.012**
**Dy1A (5)**	32.197	22.951	17.569	13.627	7.767	7.340	3.314	2.220	**2.123**	2.517	10.710	8.987	2.474
**Dy1B (5)**	31.434	22.618	18.554	13.714	8.950	8.544	3.301	**2.186**	2.235	3.041	10.409	9.117	2.653
**Dy1 (6)**	33.637	22.257	16.216	16.204	8.183	7.223	3.641	3.019	4.137	3.457	11.099	8.242	**2.654**

Table S12.- Continuous Shape Measurements for the DyO_8 coordination environment in compound **5** (fragment B).

OP-8	1 D8h	Octagon
HPY-8	2 C7v	Heptagonal pyramid
HBPY-8	3 D6h	Hexagonal bipyramid
CU-8	4 Oh	Cube
SAPR-8	5 D4d	Square antiprism
TDD-8	6 D2d	Triangular dodecahedron
JGBF-8	7 D2d	Johnson – Gyrobifastigium (J26)
JETBPY-8	8 D3h	Johnson – Elongated triangular bipyramid (J14)
JBTP-8	9 C2v	Johnson – Biaugmented trigonal prism (J50)
BTPR-8	10 C2v	Biaugmented trigonal prism
JSD-8	11 D2d	Snub disphenoid (J84)
TT-8	12 Td	Triakis tetrahedron
ETBPY-8	13 D3h	Elongated trigonal bipyramid (see 8)

Structure [ML8]	OP-8	HPY-8	HBPY-8	CU-8	SAPR-8	TDD-8	JGBF-8	JETBPY-8	JBTP-8	BTPR-8	JSD-8	TT-8	ETBPY-8
**Dy1B (5)**	30.494	22.082	15.118	11.415	3.849	3.793	13.004	22.529	2.370	**1.603**	4.237	12.185	22.115

S3. Additional Figures.

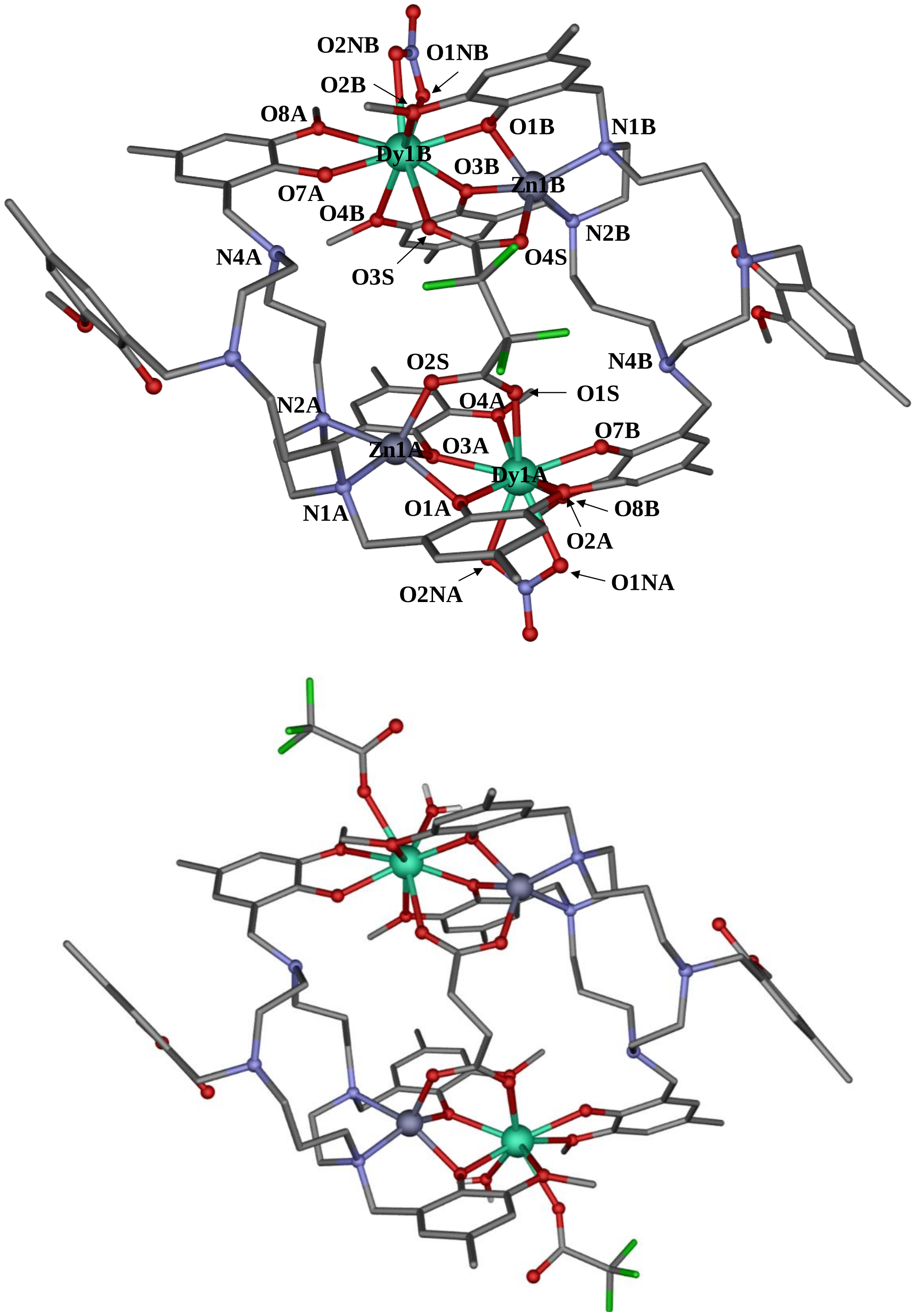

Figure S1.- Labelled coordination environment for Zn^{II} and Dy^{III} ions in **1** (top) and perspective view for **4b** (bottom). The rest of the structures follow the same labelling. For hfac, $CF_3CO_2^-$ and dbm "H", "T" and "C/D" letters are used respectively. In case of compound **2**, the two other independent molecules are labelled with C, D, E and F letters.

S4. Experimental XRPD.

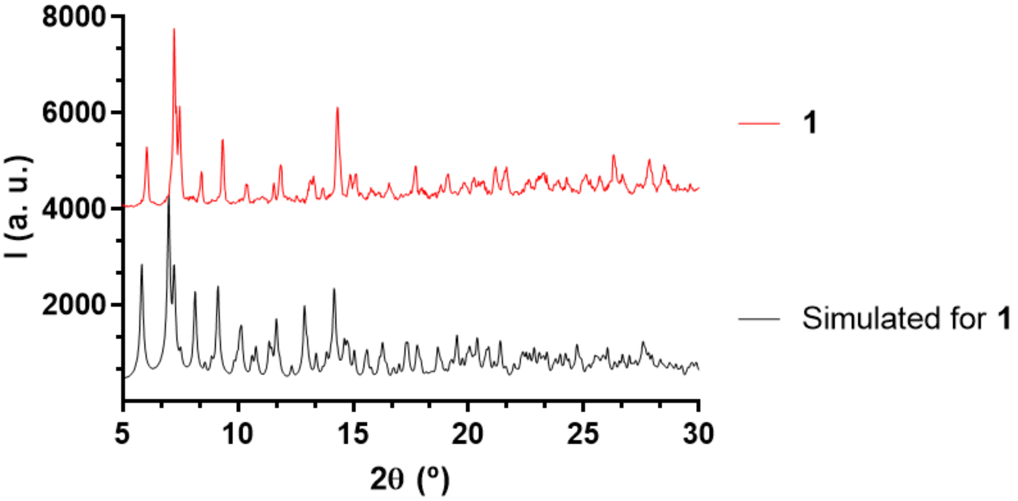

Figure S2.- For **1**, simulated pattern from single-crystal X-ray diffraction (black line) and experimental XRPD (red).

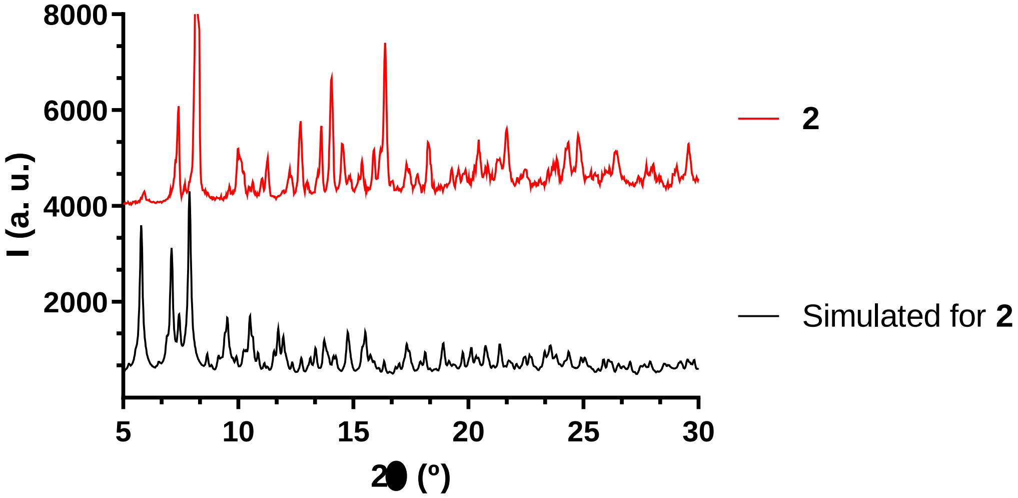

Figure S3.- For **2**, simulated pattern from single-crystal X-ray diffraction (black line) and experimental XRPD (red).

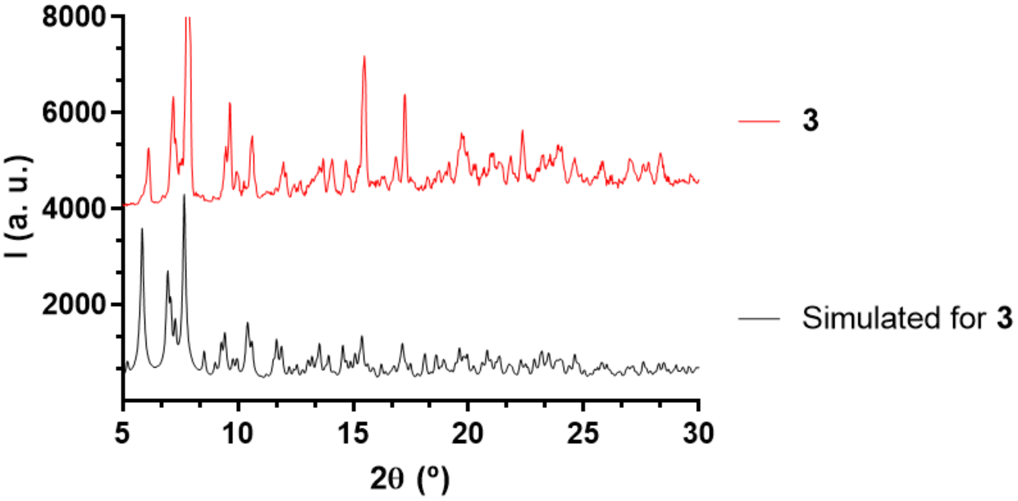

Figure S4.- For **3**, simulated pattern from single-crystal X-ray diffraction (black line) and experimental XRPD (red).

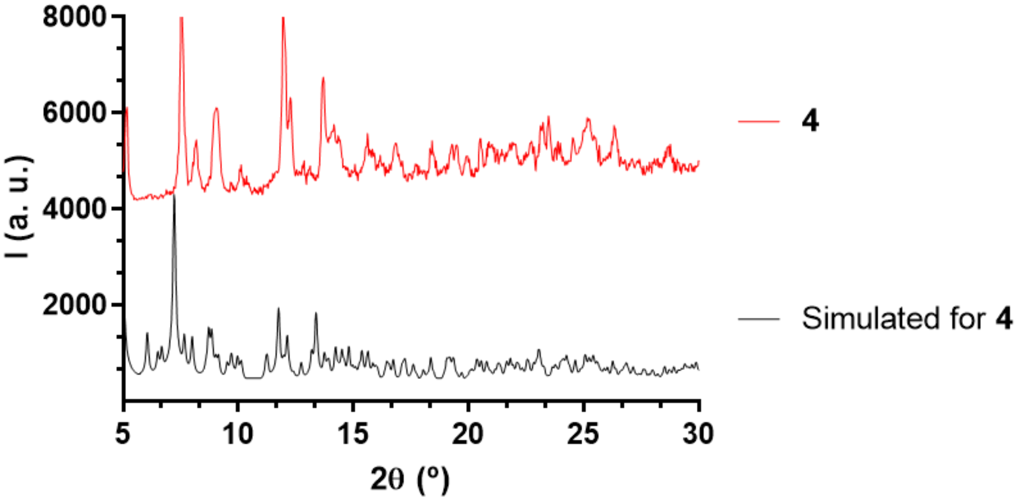

Figure S5.- For **4**, simulated pattern from single-crystal X-ray diffraction (black line) and experimental XRPD (red).

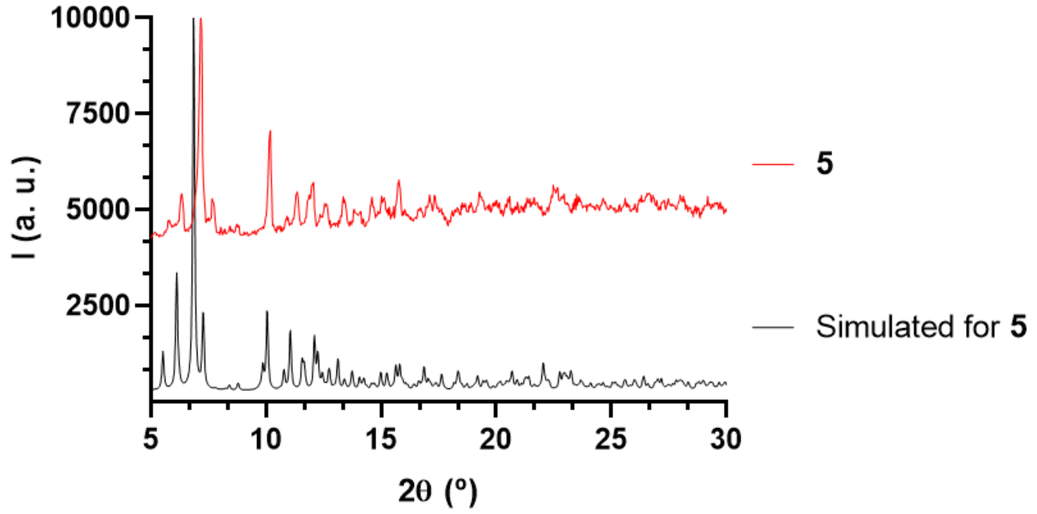

Figure S6.- For **5**, simulated pattern from single-crystal X-ray diffraction (black line) and experimental XRPD (red).

S5. Magnetic measurements.

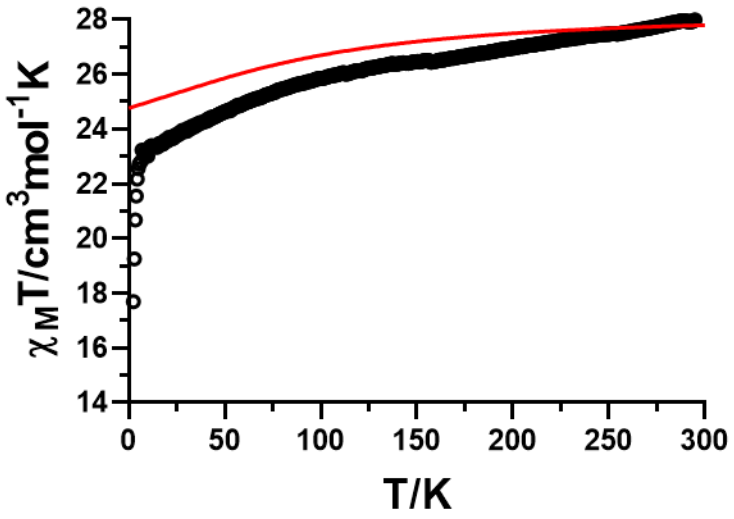

Figure S7.- For complex **1**, the experimental temperature dependence of the $\chi_M T$ product under a magnetic field of 0.1 T and the SINGLE_ANISO simulated data in zero magnetic field obtained using the results of ab initio calculations (solid red line).

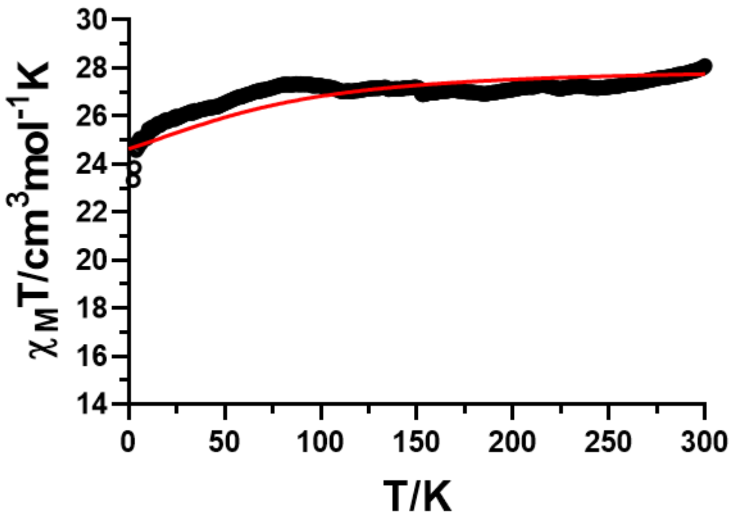

Figure S8.- For complex **2**, the experimental temperature dependence of the $\chi_M T$ product under a magnetic field of 0.1 T and the SINGLE_ANISO simulated data in zero magnetic field obtained using the results of ab initio calculations (solid red line).

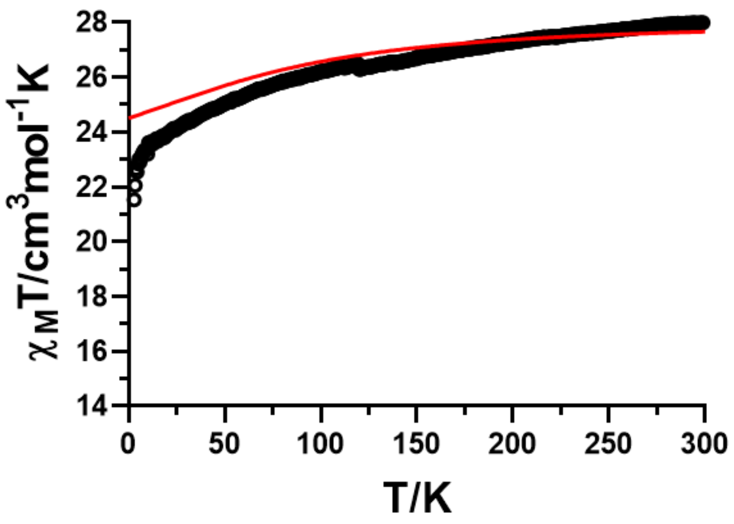

Figure S9.- For complex **3**, the experimental temperature dependence of the $\chi_M T$ product under a magnetic field of 0.1 T and the SINGLE_ANISO simulated data in zero magnetic field obtained using the results of ab initio calculations (solid red line).

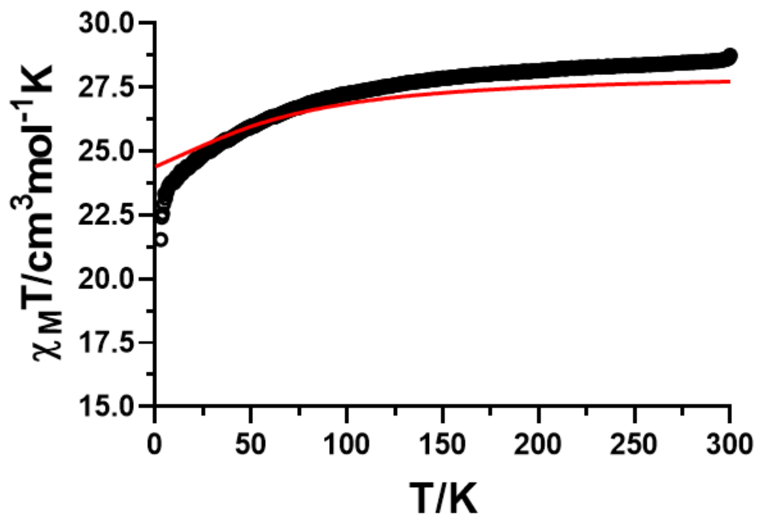

Figure S10.- For complex **4**, the experimental temperature dependence of the $\chi_M T$ product under a magnetic field of 0.1 T and the SINGLE_ANISO simulated data in zero magnetic field obtained using the results of ab initio calculations (solid red line).

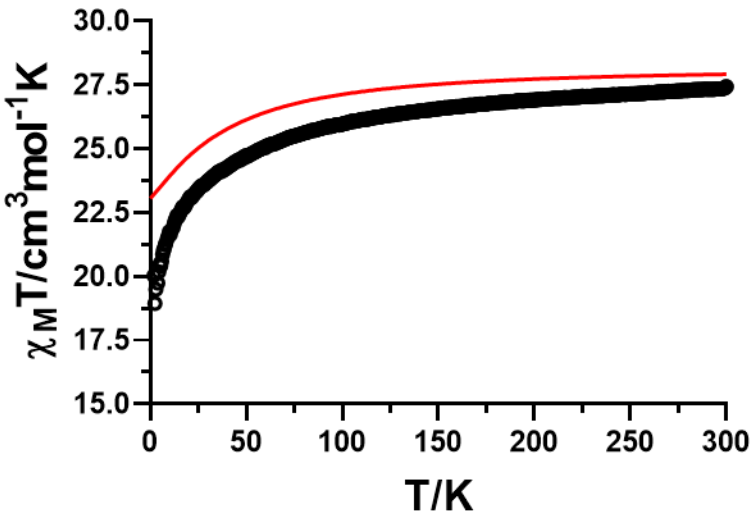

Figure S11.- For complex **5**, the experimental temperature dependence of the $\chi_M T$ product under a magnetic field of 0.1 T and the SINGLE_ANISO simulated data in zero magnetic field obtained using the results of ab initio calculations (solid red line).

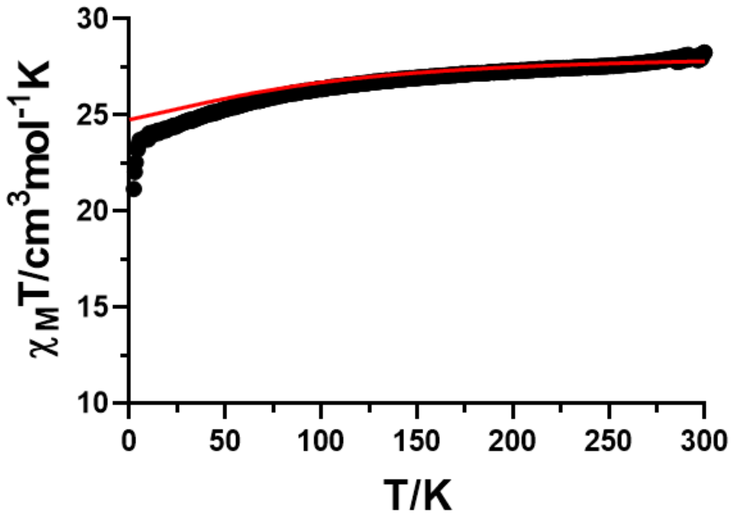

Figure S12.- For complex **6**, the experimental temperature dependence of the $\chi_M T$ product under a magnetic field of 0.1 T and the SINGLE_ANISO simulated data in zero magnetic field obtained using the results of ab initio calculations (solid red line).

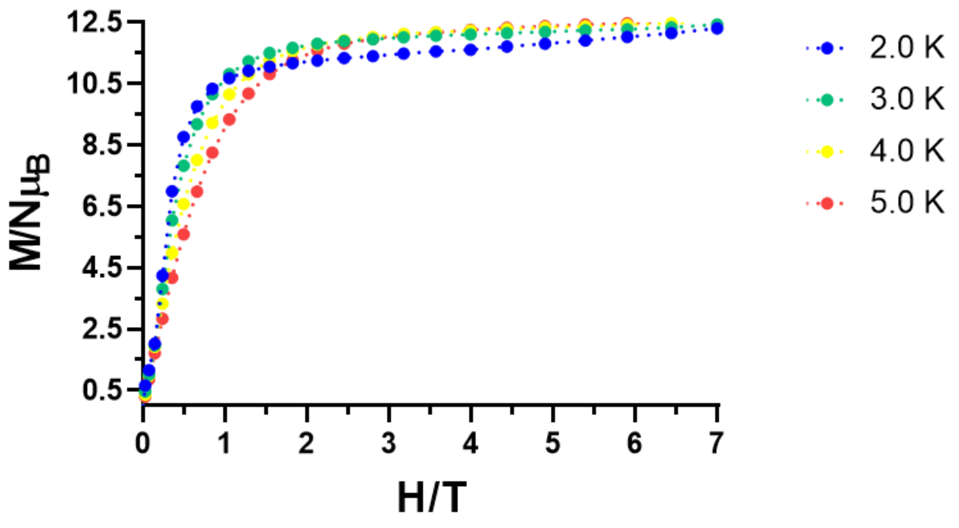

Figure S13.- Magnetization curves in the 2-5 K temperature range for compound **1**. Dashed lines are a guide to the eye.

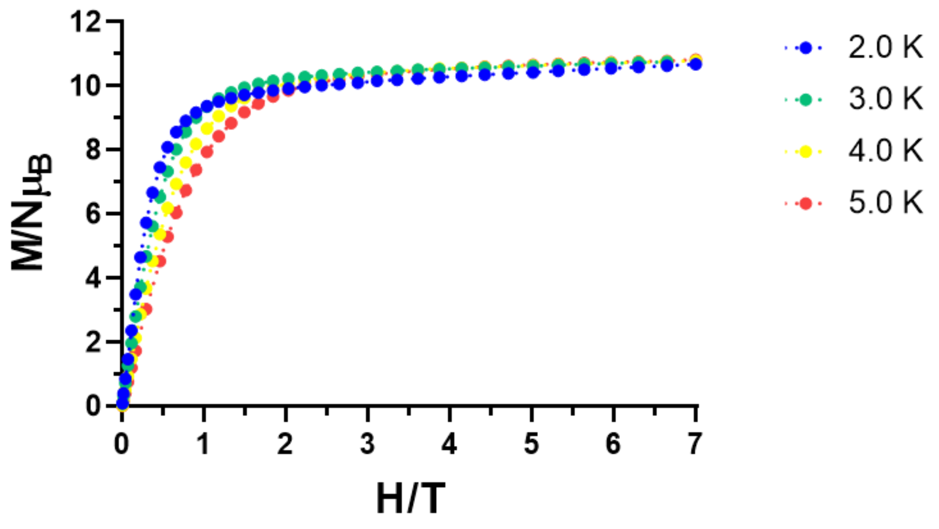

Figure S14.- Magnetization curves in the 2-5 K temperature range for compound **2**. Dashed lines are a guide to the eye.

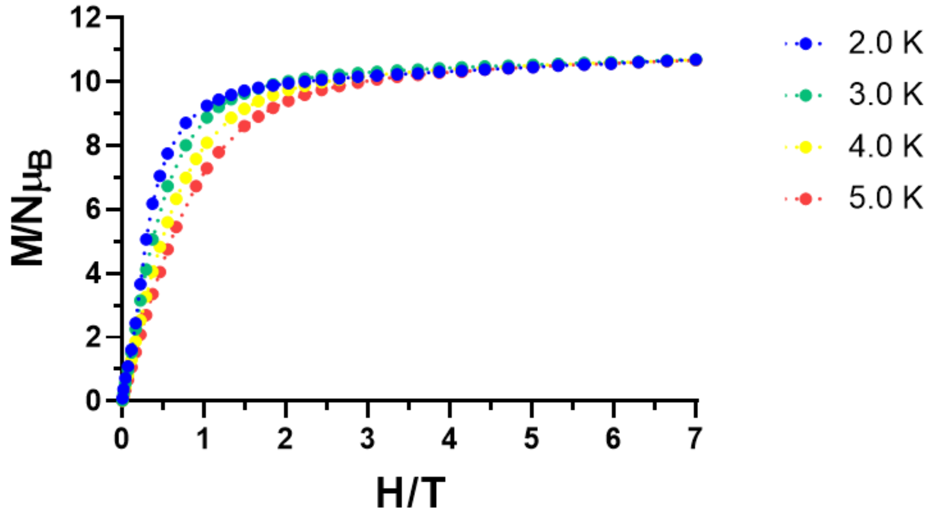

Figure S15.- Magnetization curves in the 2-5 K temperature range for compound **3**. Dashed lines are a guide to the eye.

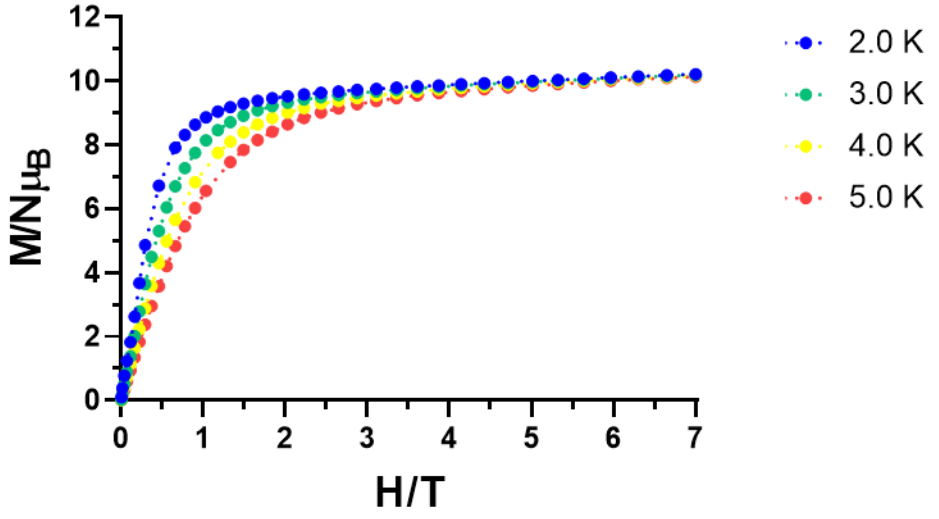

Figure S16.- Magnetization curves in the 2-5 K temperature range for compound **4**. Dashed lines are a guide to the eye.

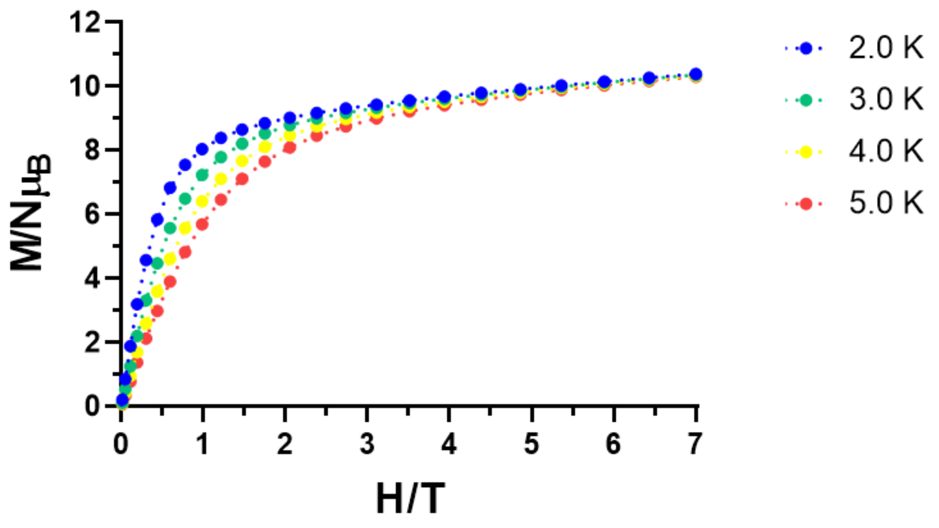

Figure S17.- Magnetization curves in the 2-5 K temperature range for compound **5**. Dashed lines are a guide to the eye.

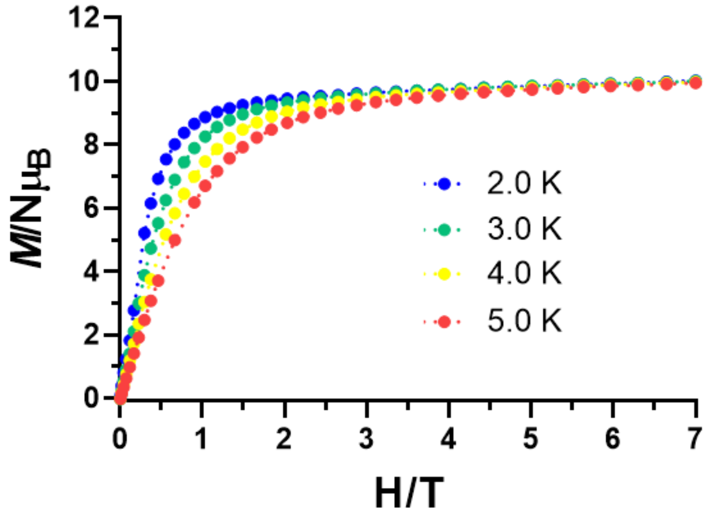

Figure S18.- Magnetization curves in the 2-5 K temperature range for compound **6**. Dashed lines are a guide to the eye.

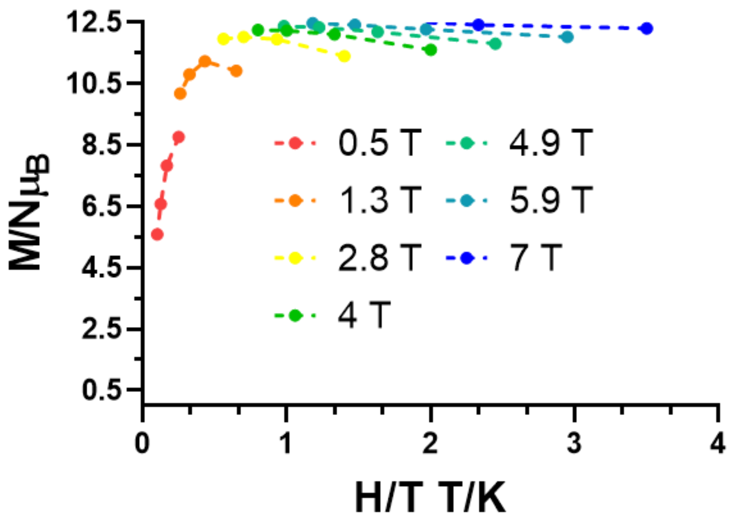

Figure S19.- Isothermal reduced magnetization curves in the 2-5 K temperature range for compound **1**. Dashed lines are a guide to the eye.

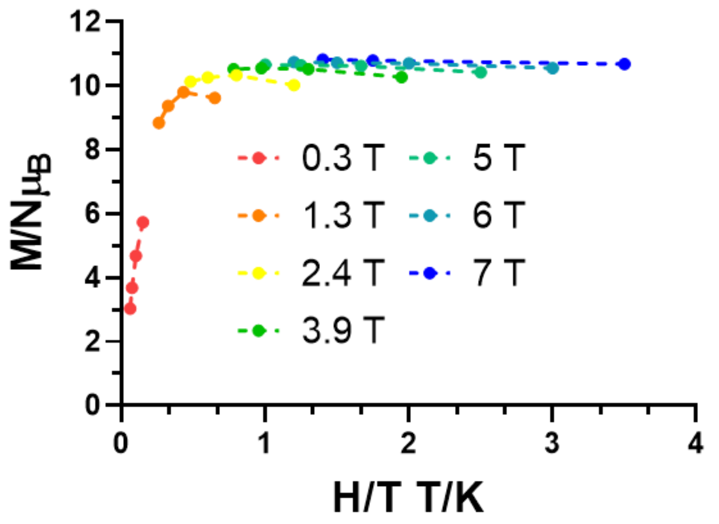

Figure S20.- Isothermal reduced magnetization curves in the 2-5 K temperature range for compound **2**. Dashed lines are a guide to the eye.

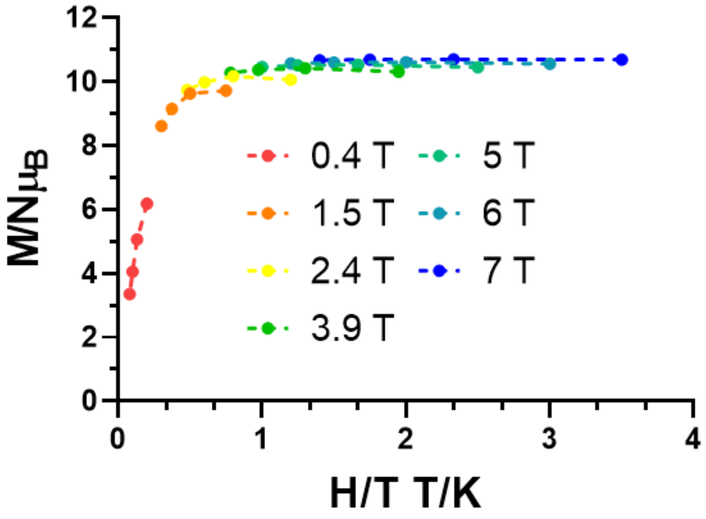

Figure S21.- Isothermal reduced magnetization curves in the 2-5 K temperature range for compound **3**. Dashed lines are a guide to the eye.

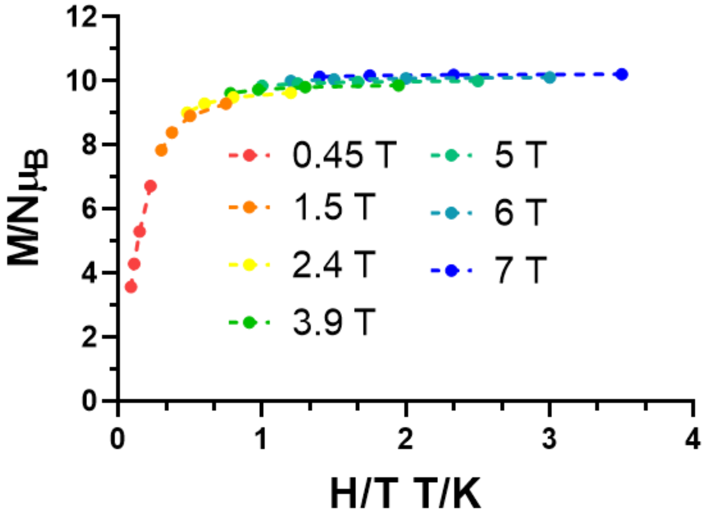

Figure S22.- Isothermal reduced magnetization curves in the 2-5 K temperature range for compound **4**. Dashed lines are a guide to the eye.

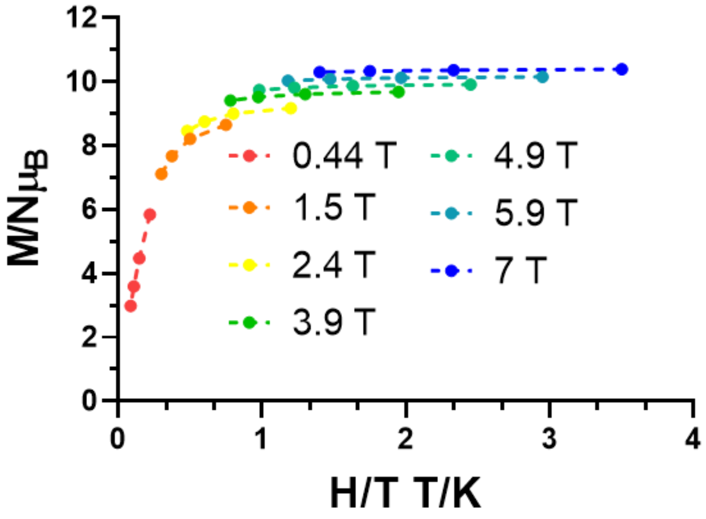

Figure S23.- Isothermal reduced magnetization curves in the 2-5 K temperature range for compound **5**. Dashed lines are a guide to the eye.

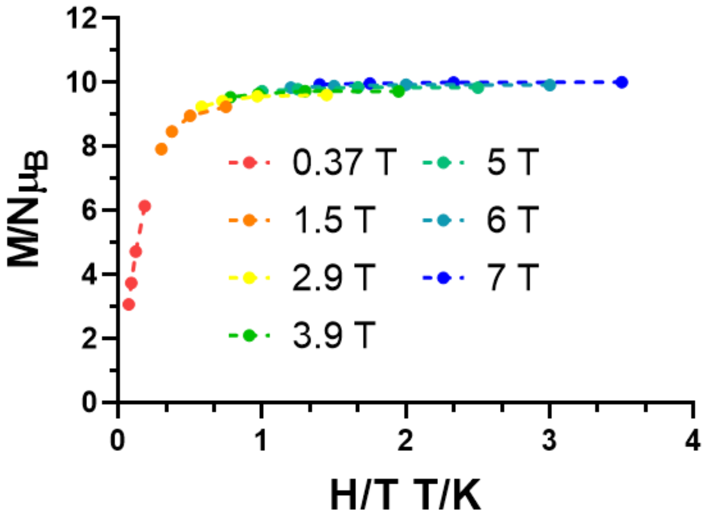

Figure S24.- Isothermal reduced magnetization curves in the 2-5 K temperature range for compound **6**. Dashed lines are a guide to the eye.

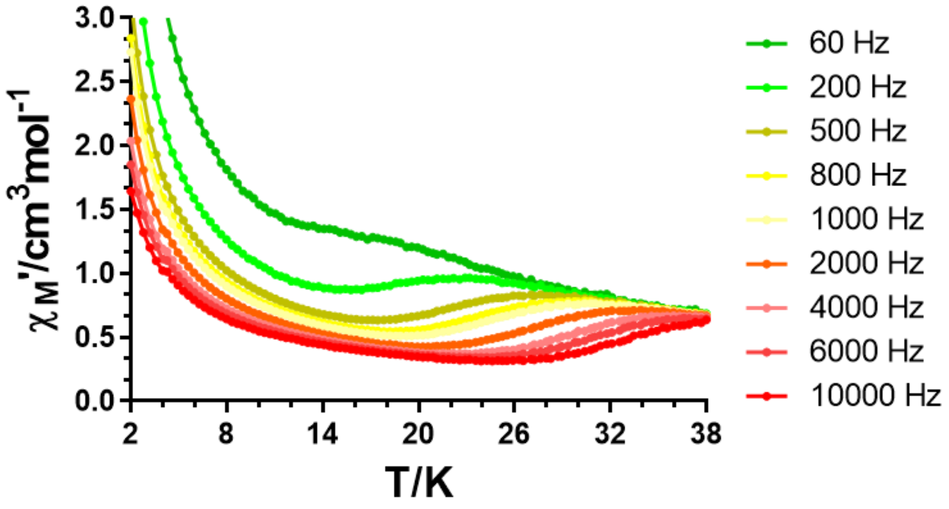

Figure S25.- Temperature dependence of the in phase components of the *ac* susceptibility in a zero applied field for **1**.

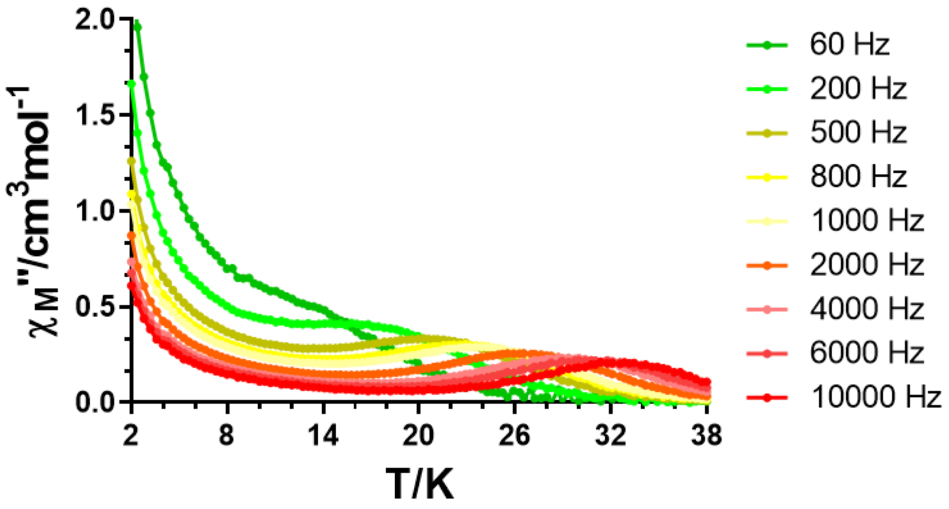

Figure S26.- Temperature dependence of the out-of-phase components of the *ac* susceptibility in a zero applied field for **1**.

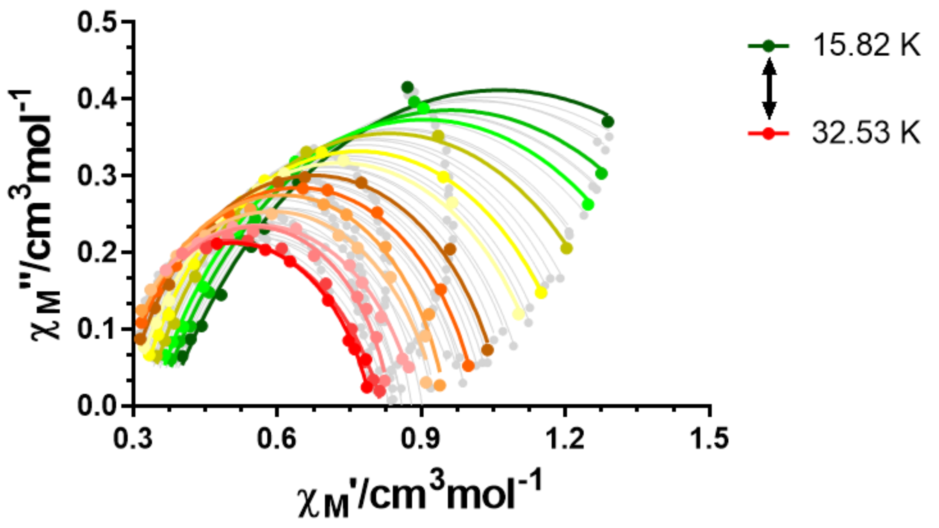

Figure S27.- Cole-Cole plots under zero field for **1**. Solid lines represent the best fit to the generalized Debye model.

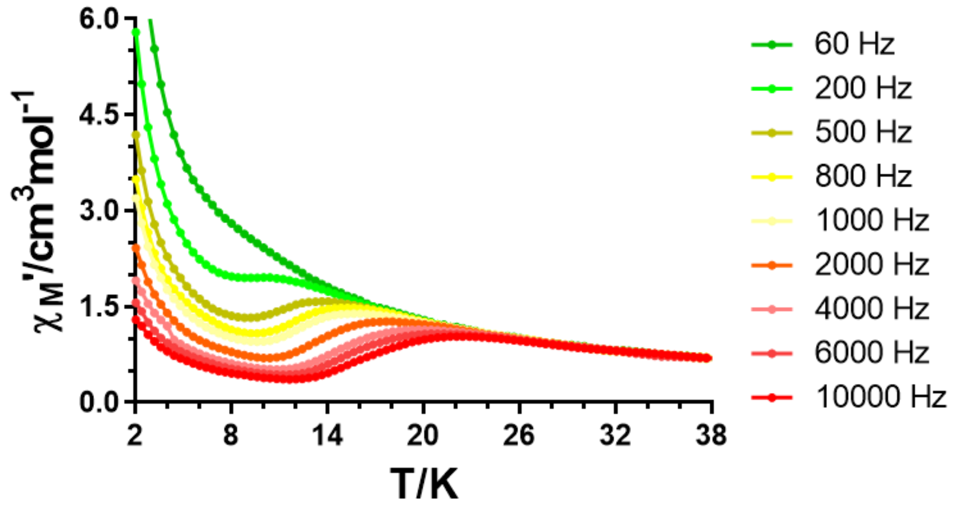

Figure S28.- Temperature dependence of the in phase components of the *ac* susceptibility in a zero applied field for **2**.

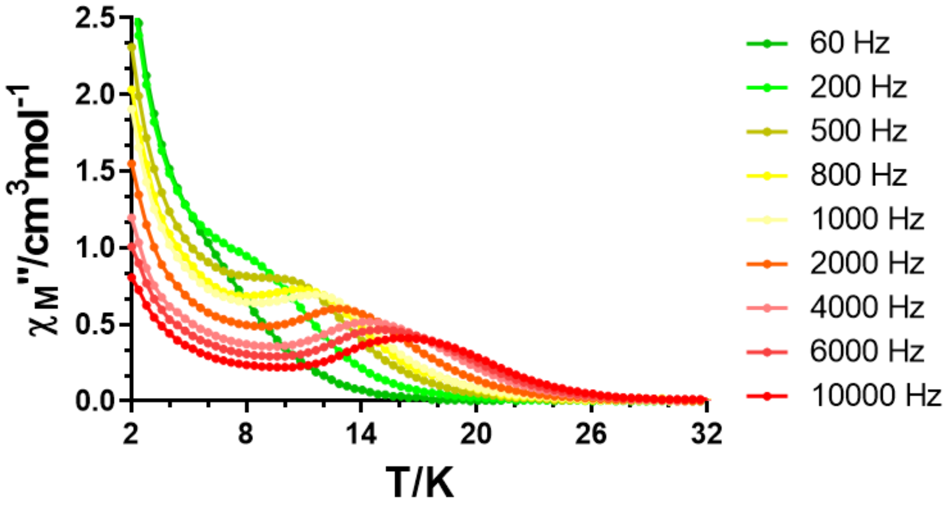

Figure S29.- Temperature dependence of the out-of-phase components of the *ac* susceptibility in a zero applied field for **2**.

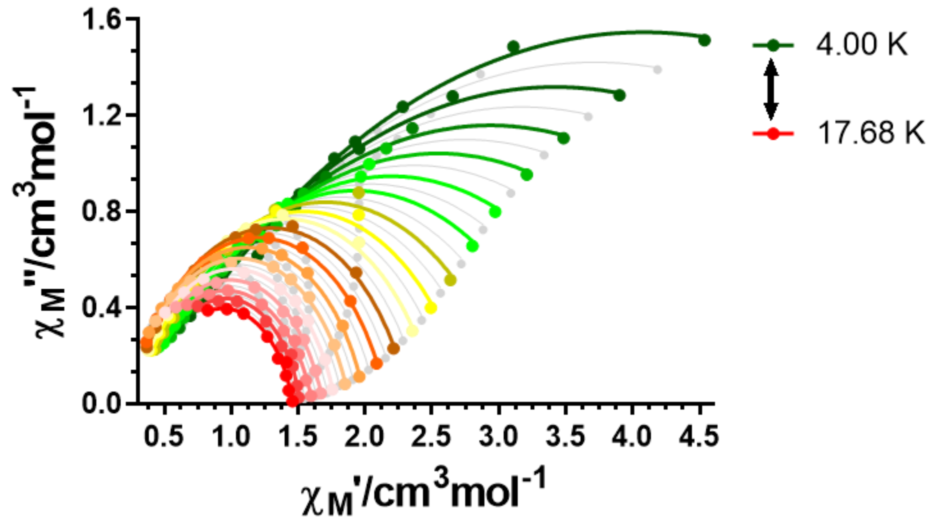

Figure S30.- Cole-Cole plots under zero field for **2**. Solid lines represent the best fit to the generalized Debye model.

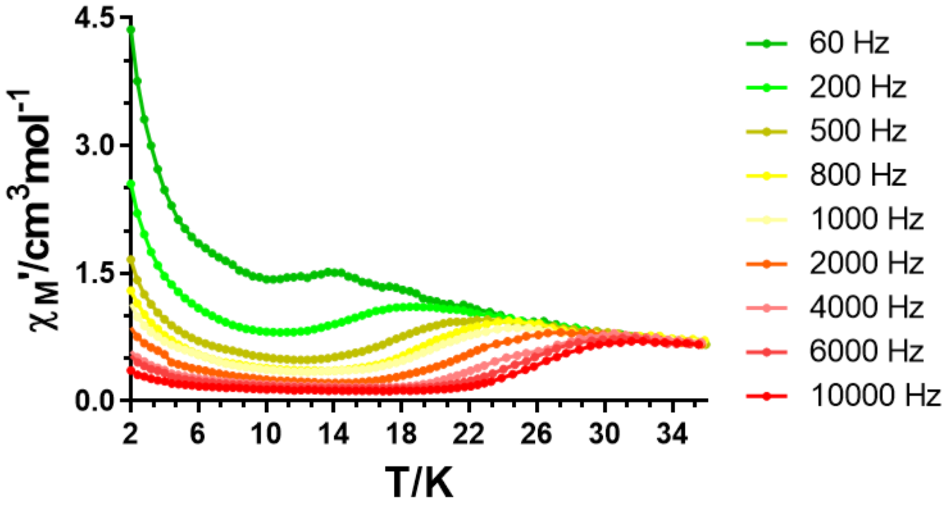

Figure S31.- Temperature dependence of the in phase components of the *ac* susceptibility in a zero applied field for **3**.

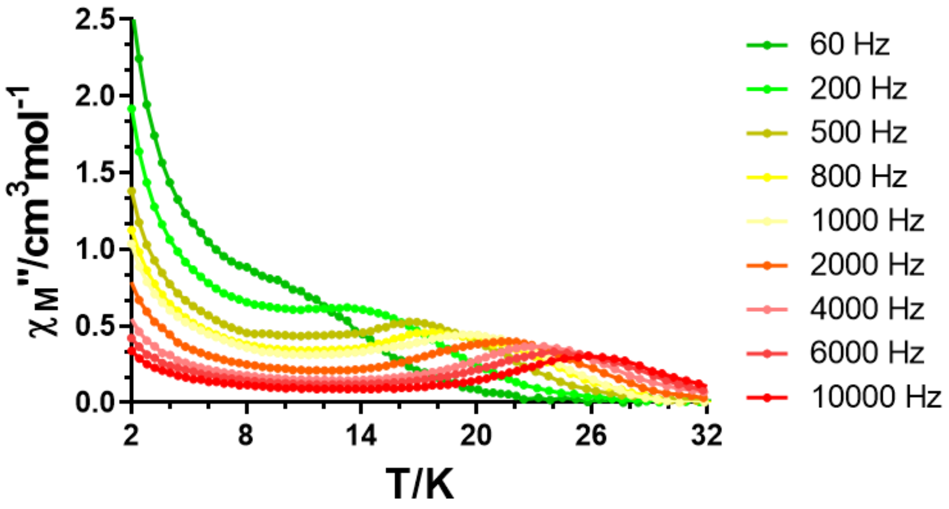

Figure S32.- Temperature dependence of the out-of-phase components of the *ac* susceptibility in a zero applied field for **3**.

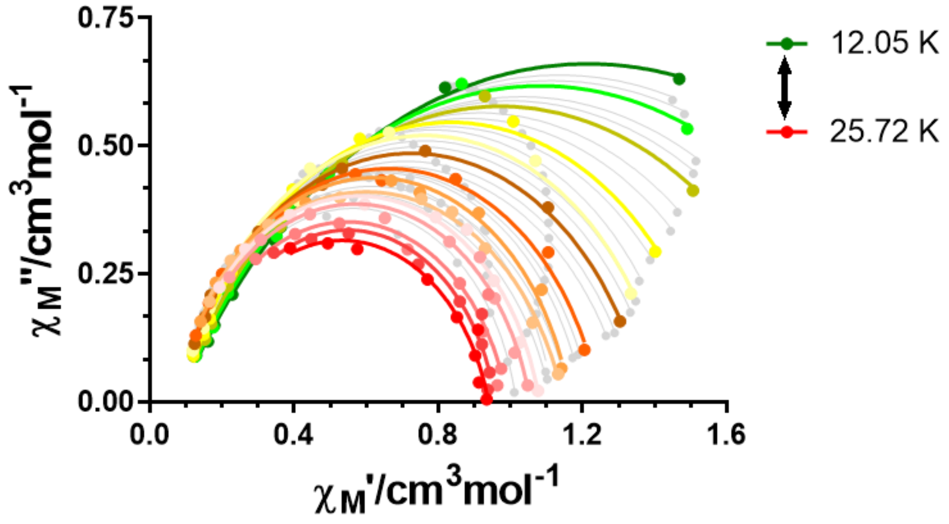

Figure S33.- Cole-Cole plots under zero field for **3**. Solid lines represent the best fit to the generalized Debye model.

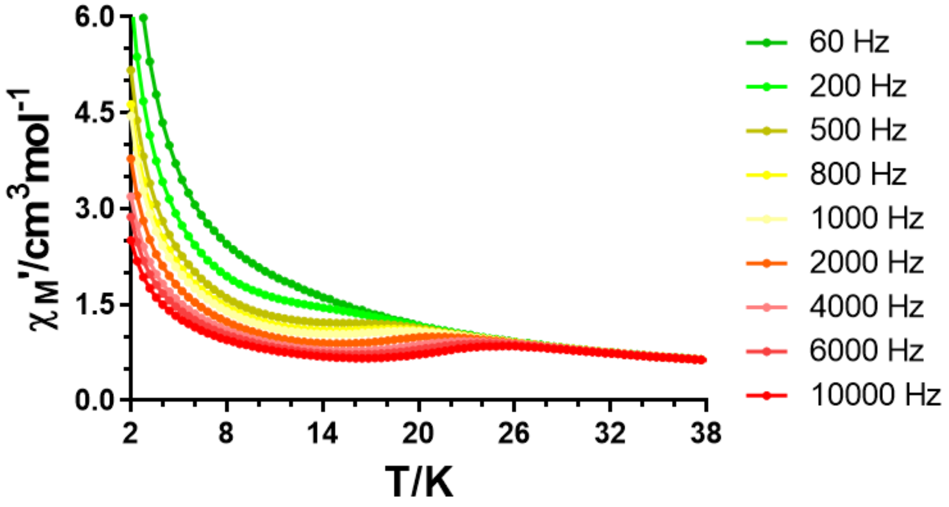

Figure S34.- Temperature dependence of the in phase components of the *ac* susceptibility in a zero applied field for **4**.

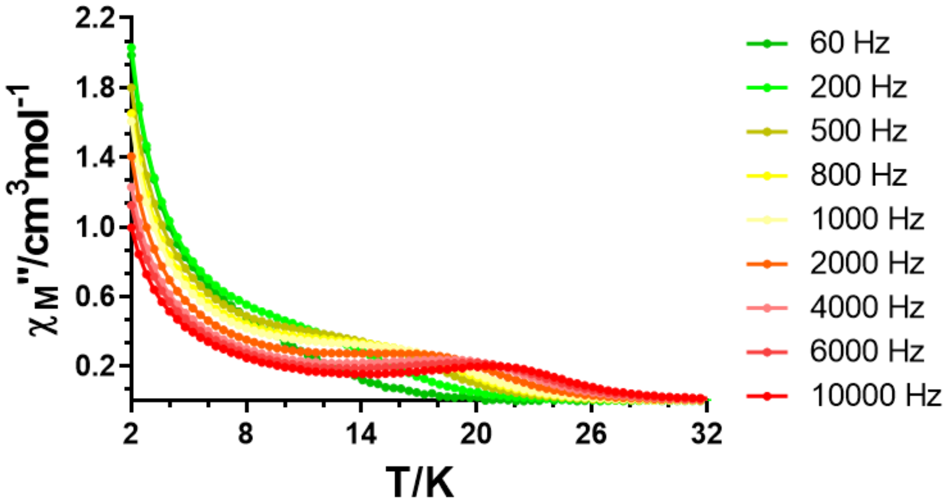

Figure S35.- Temperature dependence of the out-of-phase components of the *ac* susceptibility in a zero applied field for **4**.

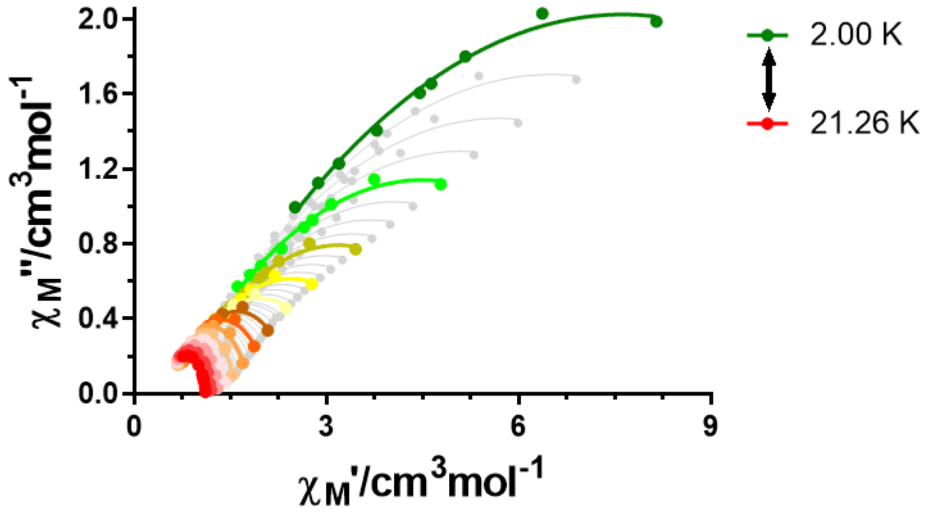

Figure S36.- Cole-Cole plots under zero field for **4**. Solid lines represent the best fit to the generalized Debye model.

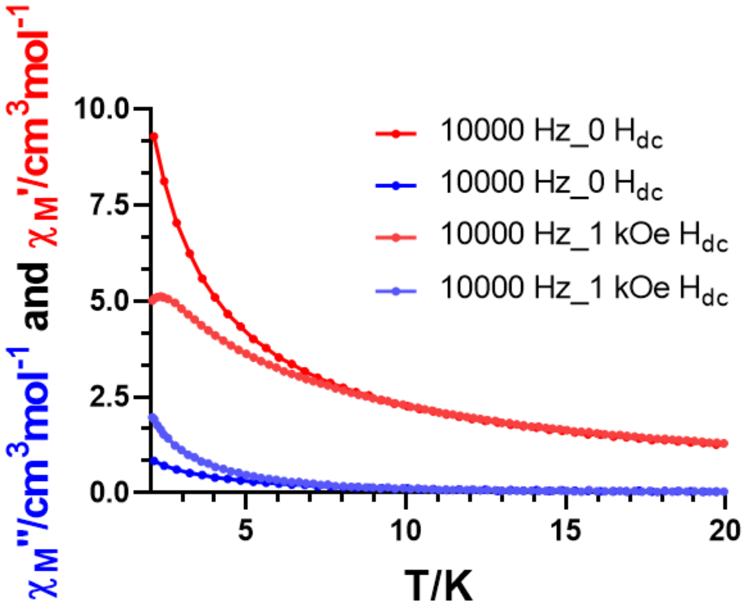

Figure S37.- Temperature dependence of the in phase χ_M' (red) and out-of-phase (blue) susceptibility signals for complex **5** under zero and 1 kOe applied *dc* field.

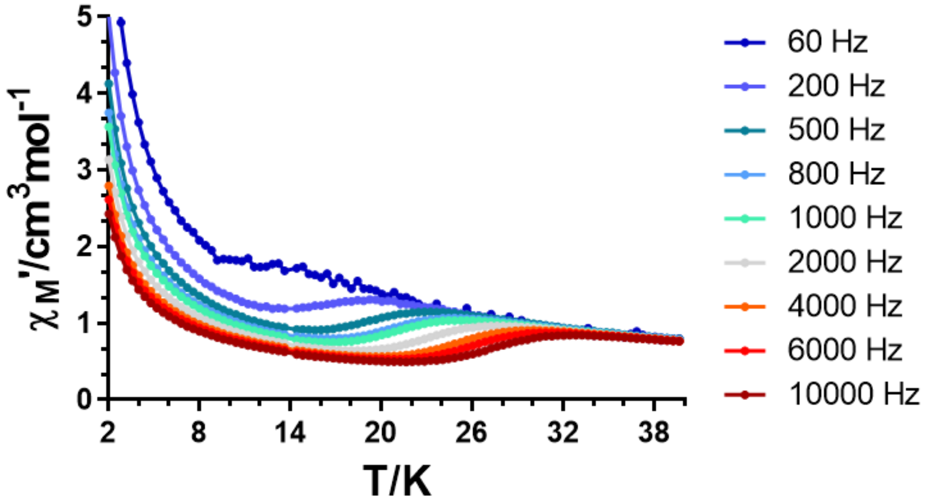

Figure S38.- Temperature dependence of the in phase components of the *ac* susceptibility in a zero applied field for **6**.

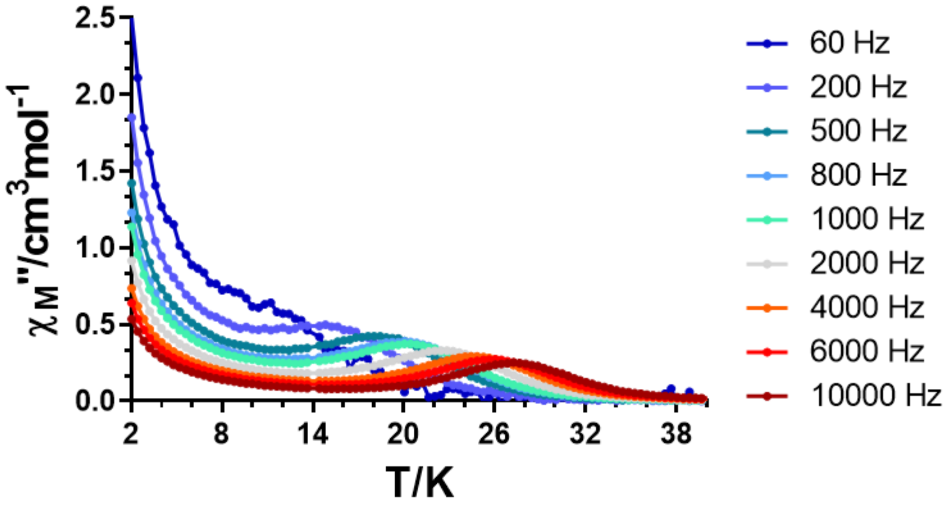

Figure S39.- Temperature dependence of the out-of-phase components of the *ac* susceptibility in a zero applied field for **6**.

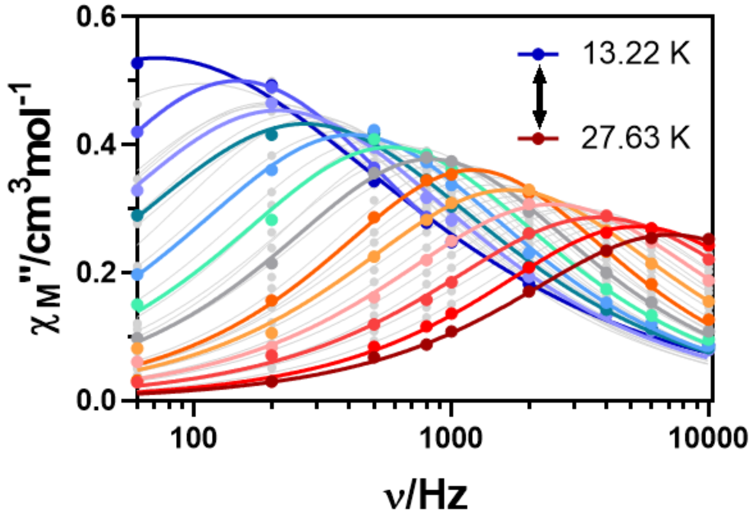

Figure S40.- Variable-temperature frequency dependence of the χ''_M signal at zero applied field for **6**.

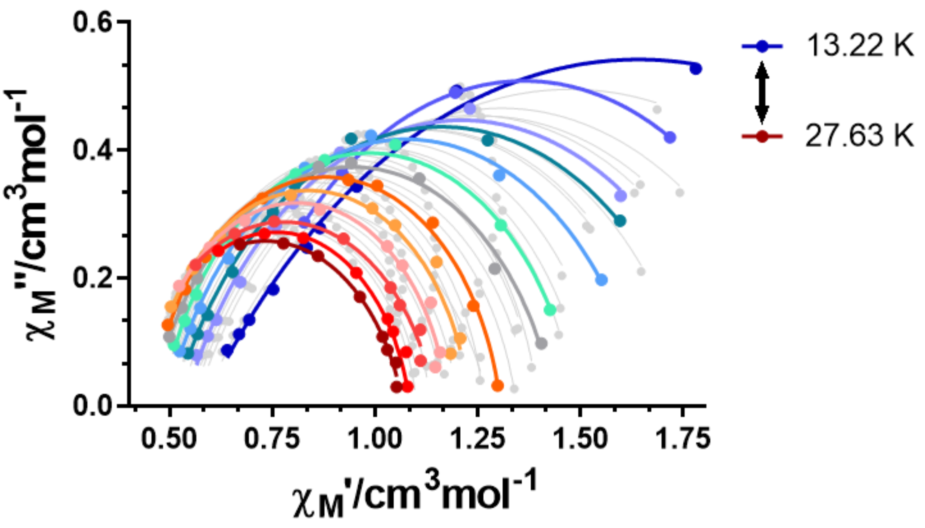

Figure S41.- Cole-Cole plots under zero field for **6**. Solid lines represent the best fit to the generalized Debye model.

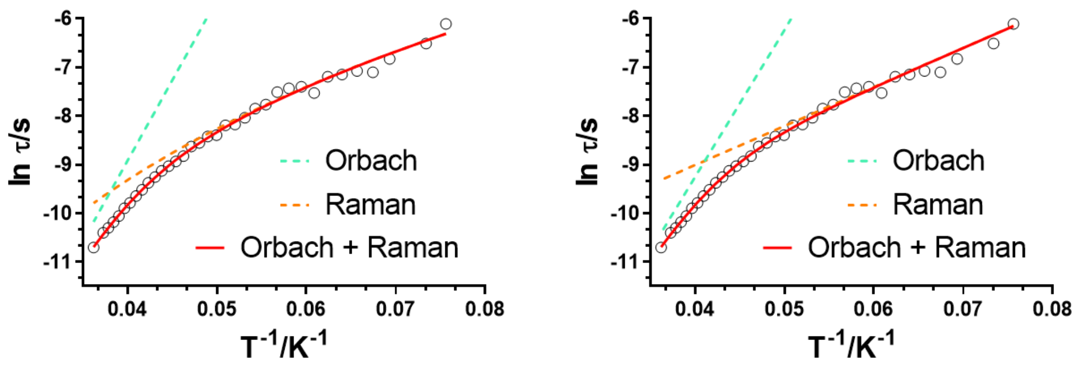

Figure S42.- Arrhenius plot for relaxation times using eq. 1 (left) and eq. 2 (right) for compound **6**. Equations can be found in the manuscript.

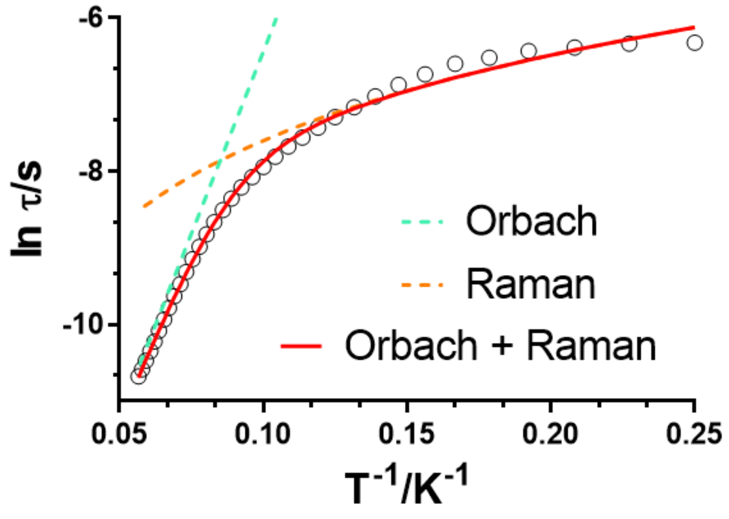

Figure S43.- Arrhenius plot for relaxation times considering the simultaneous presence of Orbach and Raman relaxation pathways: U_{eff} = 95±2 K, τ_0 = (1.17±0.10)·10^{-7} s, B = 49±21 s^{-1}K$^{-1.6}$ and n = 1.6±0.2.

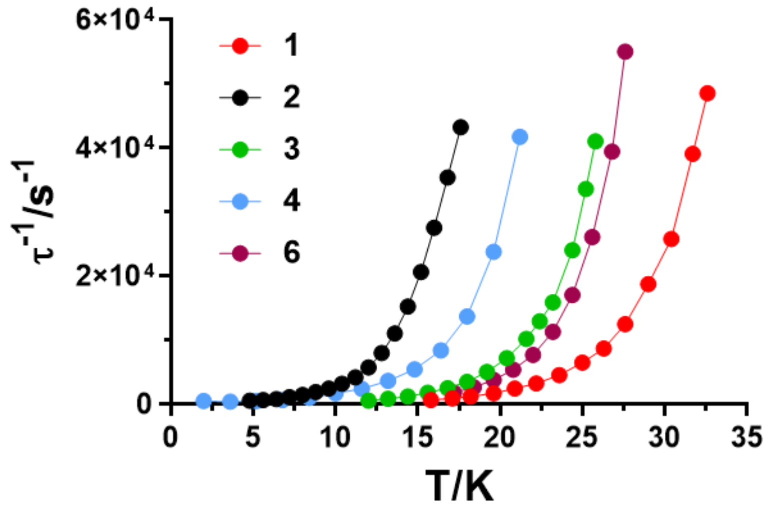

Figure S44.- Temperature dependence of the inverse of the relaxation times for compounds **1-4** and **6**. Solid lines are a guide to the eye.

As mentioned in the manuscript, relaxation times were fitted to eq. 1 and eq. 2 that are shown in the main text. The obtained parameters are summarized in Tables 1 and 2. However, only plots considering eq. 1 are presented in the main text. Thus, plots regarding eq. 2 are shown in the following Figures S45-S46.

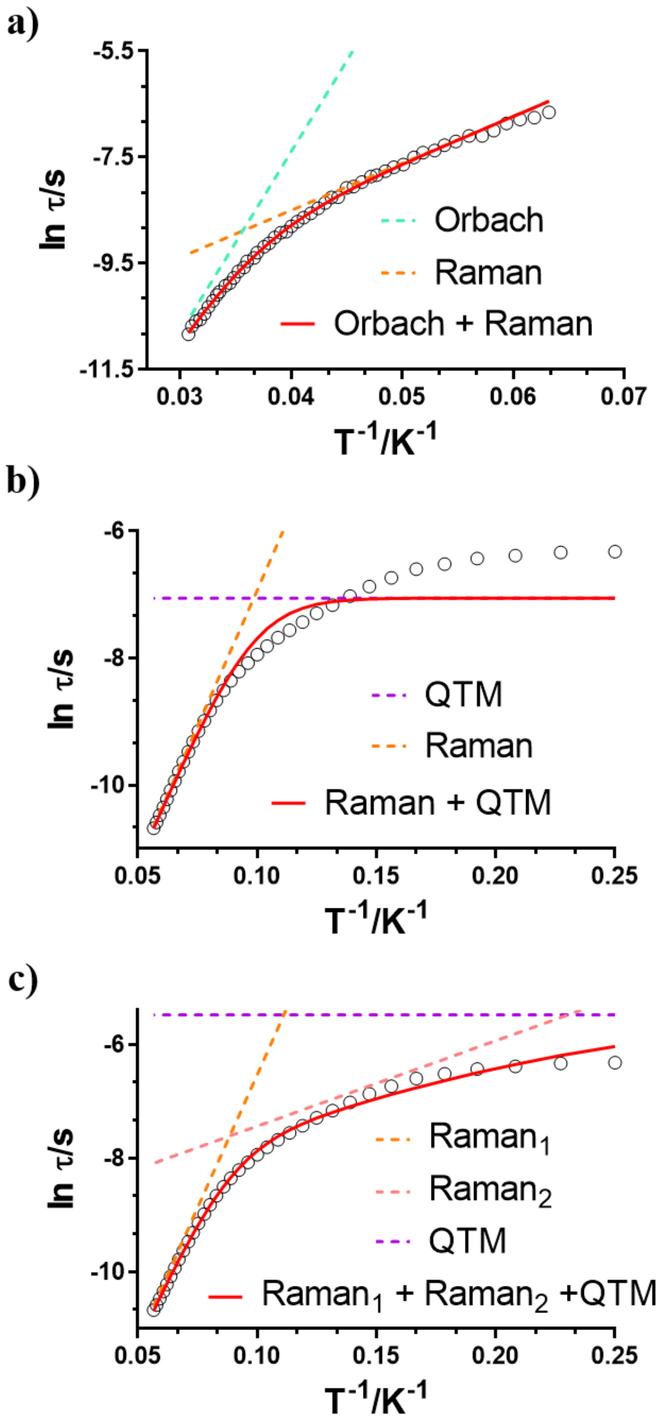

Figure S45.- Arrhenius plot for relaxation times using eq. 2 for compound **1** (a) and **2** (b, c).

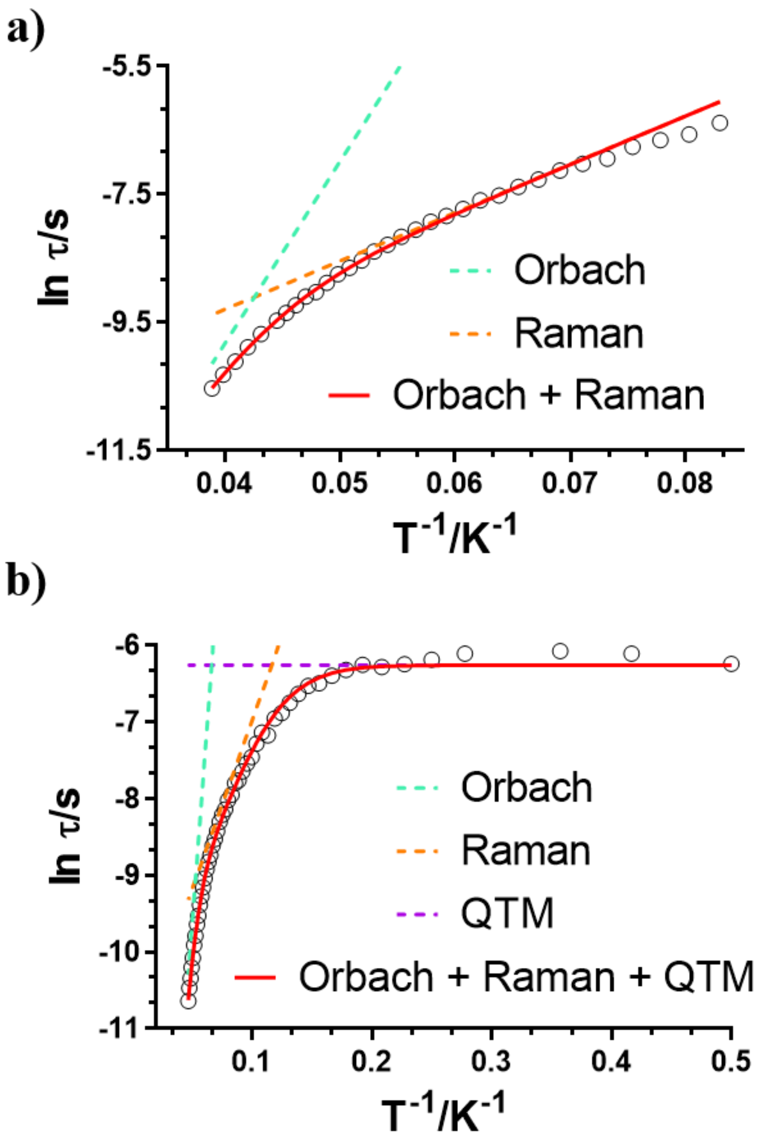

Figure S46.- Arrhenius plot for relaxation times using eq. 2 for compound **3** (a) and **4** (b).

In addition, alternative fittings were performed for all the compounds on a logarithmic scale. The following Figures S47-S57 show these fits and Tables S13-S15 summarize the obtained parameters. Overall, the obtained U_{Orbach} parameters are systematically lower than those obtained using eq.1 and eq. 2 in the main text. However, we believe that the discussion and interpretation of the data do not change when using these results and, therefore, the conclusion of the work remain unchanged.

Compound 1

Fit nº1

$$\log_{10} \tau^{-1} = \log_{10}(\tau_{0,Orbach}^{-1} \exp\left(-\frac{U_{Orbach}}{k_B T}\right) + \tau_{0,Raman}^{-1} \exp\left(-\frac{\omega_{Raman}}{k_B T}\right))$$

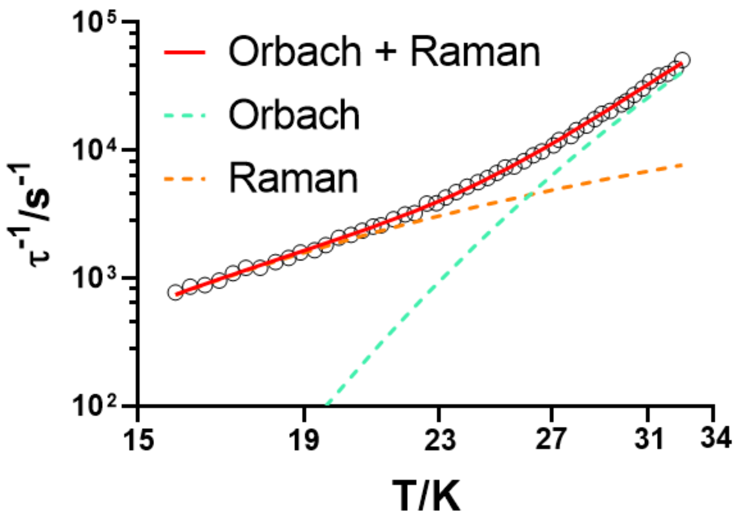

Figure S47.- log-log fit nº1 for compound **1**.

Fit nº2

$$\log_{10}\tau^{-1} = \log_{10}(\tau_{0,Orbach}^{-1}\exp\left(-\frac{U_{Orbach}}{k_BT}\right) + CT^n)$$

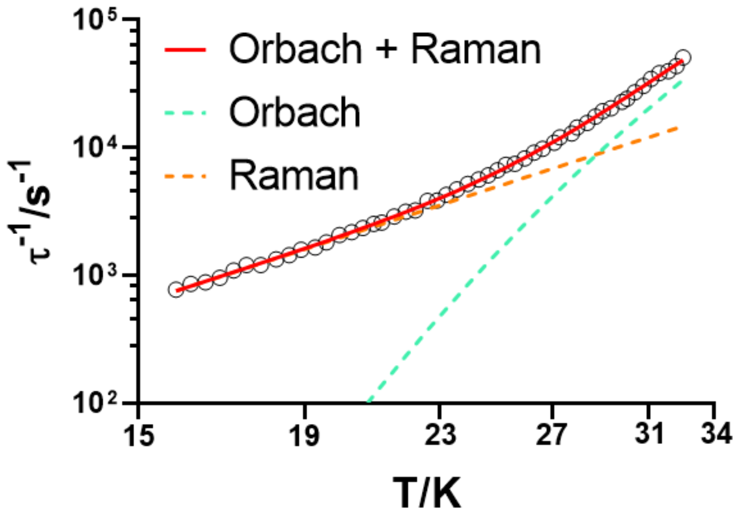

Figure S48.- log-log fit nº2 for compound **1**.

Compound 2

Fit nº1

$$\log_{10}\tau^{-1} = \log_{10}(\tau_{0,Raman}^{-1}\exp\left(-\frac{\omega_{Raman}}{k_BT}\right) + \tau_{QTM}^{-1})$$

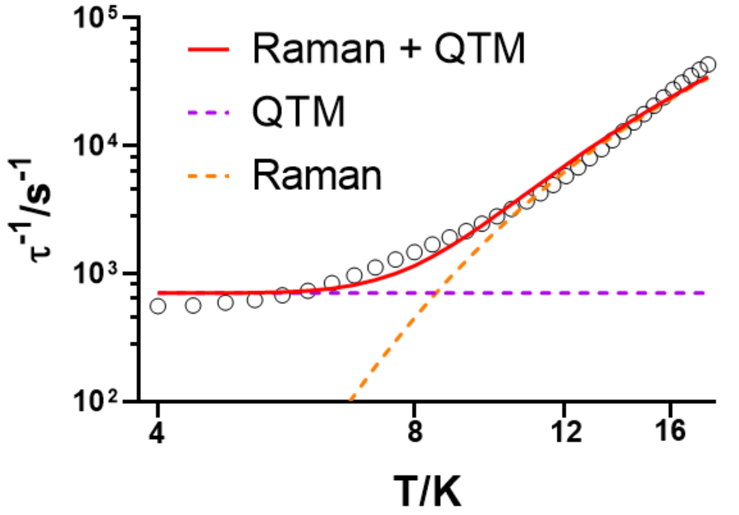

Figure S49.- log-log fit nº1 for compound **2**.

Fit nº2

$$\log_{10}\tau^{-1} = \log_{10}(\tau_{0,Raman,1}^{-1}\exp\left(-\frac{\omega_{Raman,1}}{k_BT}\right) + \tau_{0,Raman,2}^{-1}\exp\left(-\frac{\omega_{Raman,2}}{k_BT}\right) + \tau_{QTM}^{-1})$$

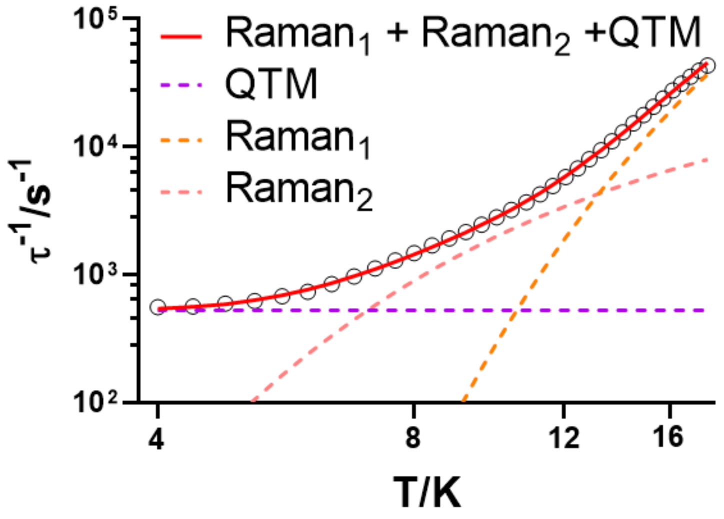

Figure S50.- log-log fit nº2 for compound **2**.

Fit nº3

$$\log_{10}\tau^{-1} = \log_{10}(\tau_{QTM}^{-1} + CT^{n})$$

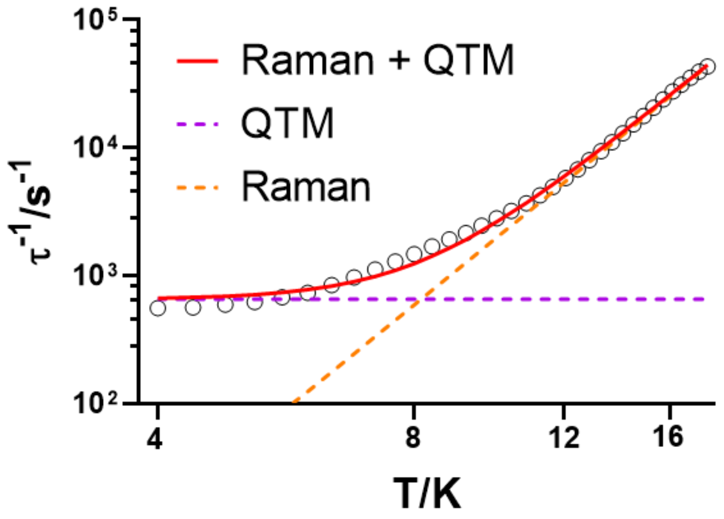

Figure S51.- log-log fit nº2 for compound **2**.

Compound 3

Fit nº1

$$\log_{10}\tau^{-1} = \log_{10}(\tau_{0,Orbach}^{-1}\exp\left(-\frac{U_{Orbach}}{k_B T}\right) + \tau_{0,Raman}^{-1}\exp\left(-\frac{\omega_{Raman}}{k_B T}\right))$$

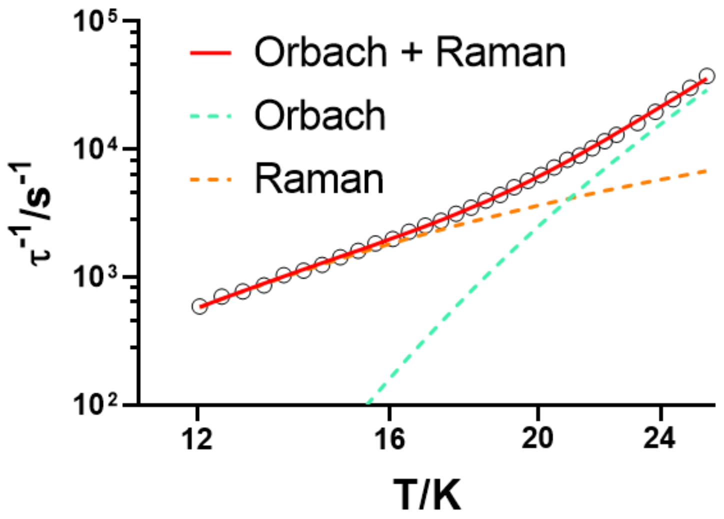

Figure S52.- log-log fit nº1 for compound **3**.

Fit nº2

$$\log_{10}\tau^{-1} = \log_{10}(\tau_{0,Orbach}^{-1}\exp\left(-\frac{U_{Orbach}}{k_B T}\right) + CT^{n})$$

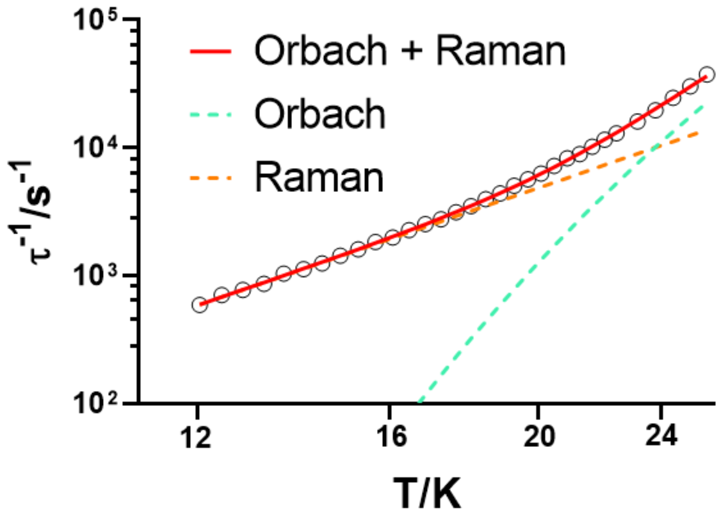

Figure S53.- log-log fit nº2 for compound **3**.

Compound 4

Fit nº1

$$\log_{10}\tau^{-1} = \log_{10}(\tau_{0,Orbach}^{-1}\exp\left(-\frac{U_{Orbach}}{k_BT}\right) + \tau_{0,Raman}^{-1}\exp\left(-\frac{\omega_{Raman}}{k_BT}\right) + \tau_{QTM}^{-1})$$

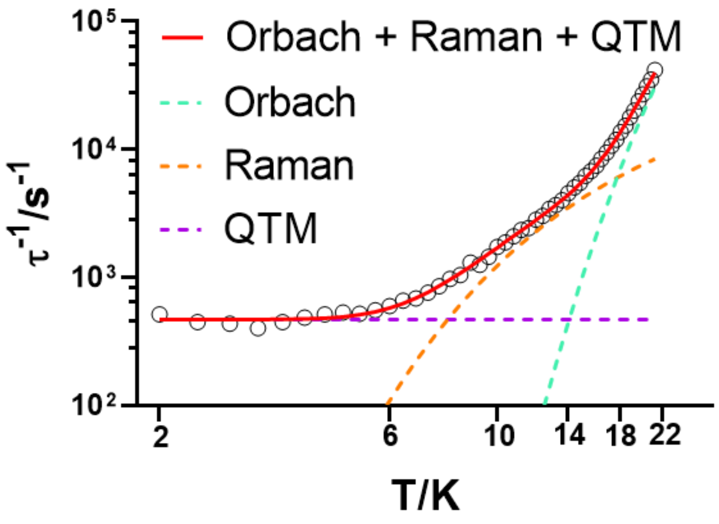

Figure S54.- log-log fit nº1 for compound **4**.

Fit nº2

$$\log_{10}\tau^{-1} = \log_{10}(\tau_{0,Orbach}^{-1}\exp\left(-\frac{U_{Orbach}}{k_BT}\right) + CT^{n} + \tau_{QTM}^{-1})$$

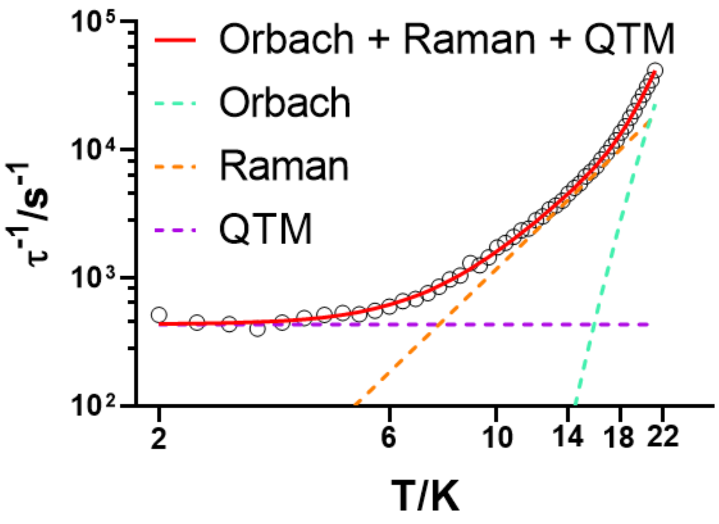

Figure S55.- log-log fit nº2 for compound **4**.

Compound 6

Fit nº1

$$\log_{10}\tau^{-1} = \log_{10}(\tau_{0,Orbach}^{-1}\exp\left(-\frac{U_{Orbach}}{k_B T}\right) + \tau_{0,Raman}^{-1}\exp\left(-\frac{\omega_{Raman}}{k_B T}\right))$$

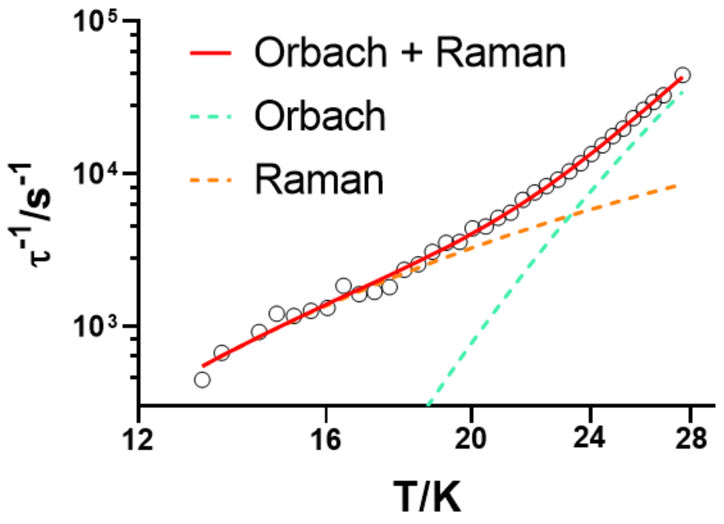

Figure S56.- log-log fit nº1 for compound **6**.

Fit nº2

$$\log_{10}\tau^{-1} = \log_{10}(\tau_{0,Orbach}^{-1}\exp\left(-\frac{U_{Orbach}}{k_B T}\right) + CT^{n})$$

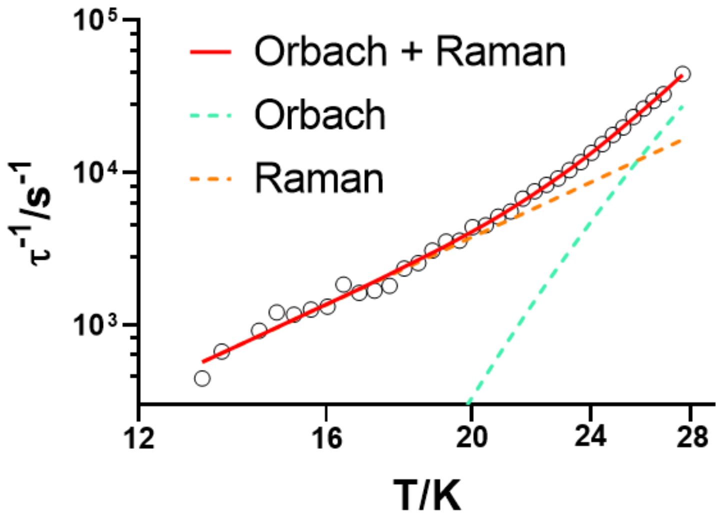

Figure S57.- log-log fit nº2 for compound **6**.

Table S13.- Best fit parameters for equations in Figures S47-S51.

	Compound 1		Compound 2		
	**Fit nº1**	**Fit nº2**	**Fit nº1**	**Fit nº2**	**Fit nº3**
τ_{QTM}, s	-	-	$(1.42\pm0.09)\cdot10^{-3}$	$(1.90\pm0.03)\cdot10^{-3}$	$(1.76\pm0.06)\cdot10^{-3}$
$\tau_{0,Raman}$, s	$(1.42\pm0.16)\cdot10^{-5}$	-	$(8.3\pm1.6)\cdot10^{-7}$	1: $(4.95\pm0.64)\cdot10^{-8}$<br>2: $(2.07\pm0.22)\cdot10^{-5}$	-
ω_{Raman}, K	72±2	-	63±2	1: 111±2<br>2: 32±1	-
C, $s^{-1}K^{-n}$	-	$(8.6\pm2.1)\cdot10^{-3}$	-	-	$(2.1\pm0.4)\cdot10^{-2}$
n	-	4.12±0.08	-	-	5.04±0.08
$\tau_{0,Orbach}$, s	$(2.64\pm0.56)\cdot10^{-9}$	$(9.86\pm2.83)\cdot10^{-10}$	-	-	-
U_{Orbach}, K	298±7	335±10	-	-	-

Table S14.- Best fit parameters for equations in Figures S52-S55.

	Compound 3		Compound 4	
	**Fit nº1**	**Fit nº2**	**Fit nº1**	**Fit nº2**
τ_{QTM}, s	-	-	$(2.13\pm0.03)\cdot10^{-3}$	$(2.31\pm0.04)\cdot10^{-3}$
$\tau_{0,Raman}$, s	$(1.70\pm0.16)\cdot10^{-5}$	-	$(2.14\pm0.20)\cdot10^{-5}$	-
ω_{Raman}, K	56±1	-	36±1	-
C, $s^{-1}K^{-n}$	-	$(1.9\pm0.3)\cdot10^{-2}$	-	0.26±0.05
n	-	4.15±0.06	-	3.65±0.07
$\tau_{0,Orbach}$, s	$(6.6\pm1.3)\cdot10^{-9}$	$(1.89\pm0.54)\cdot10^{-9}$	$(8.07\pm0.28)\cdot10^{-9}$	$(3.4\pm3.0)\cdot10^{-10}$
U_{Orbach}, K	220±5	259±7	176±7	250±18

Table S15.- Best fit parameters for equations in Figures S56-S57.

	Compound 6	
	**Fit nº1**	**Fit nº2**
τ_{QTM}, s	-	-
$\tau_{0,Raman}$, s	(9±3)·10^{-6}	-
ω_{Raman}, K	70±4	-
C, $s^{-1}K^{-n}$	-	(4±3)·10^{-3}
n	-	4.6±0.2
$\tau_{0,Orbach}$, s	(1.4±1.0)·10^{-9}	(3.5±4.8)·10^{-10}
U_{Orbach}, K	274±20	319±38

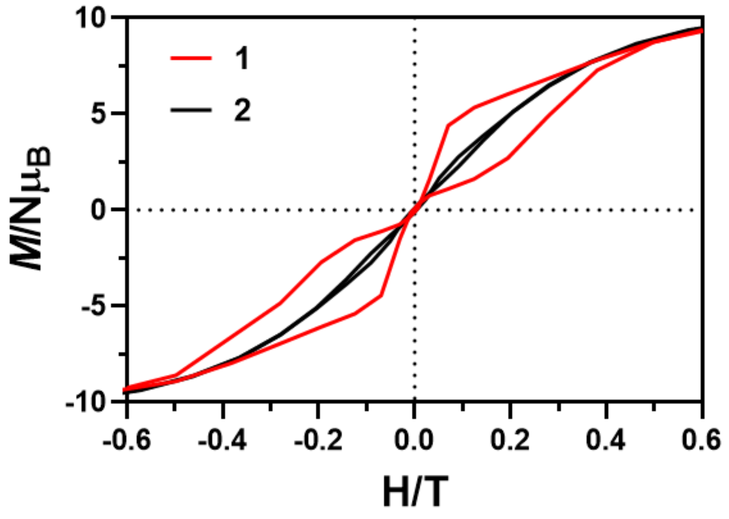

Figure S58.- Magnetic hysteresis loops for **1** and **2** for H values between -0.6 T and 0.6 T at 2 K. For **1**, the average sweep-rate in the 0.63 T to -0.63 T and -0.63 T to 0.63 T range was 61 Oe/s; for **2**, in the 0.57 T to -0.57 T and -0.57 T to 0.57 T range was 52 Oe/s.

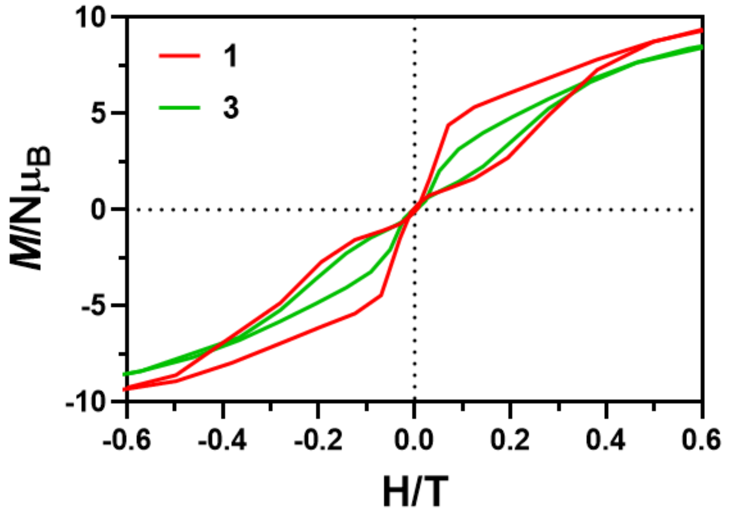

Figure S59.- Magnetic hysteresis loops for **1** and **3** for H values between -0.6 T and 0.6 T at 2 K. For **1**, the average sweep-rate in the 0.63 T to -0.63 T and -0.63 T to 0.63 T range was 61 Oe/s; for **3**, in the 0.57 T to -0.57 T and -0.57 T to 0.69 T range was 52 Oe/s.

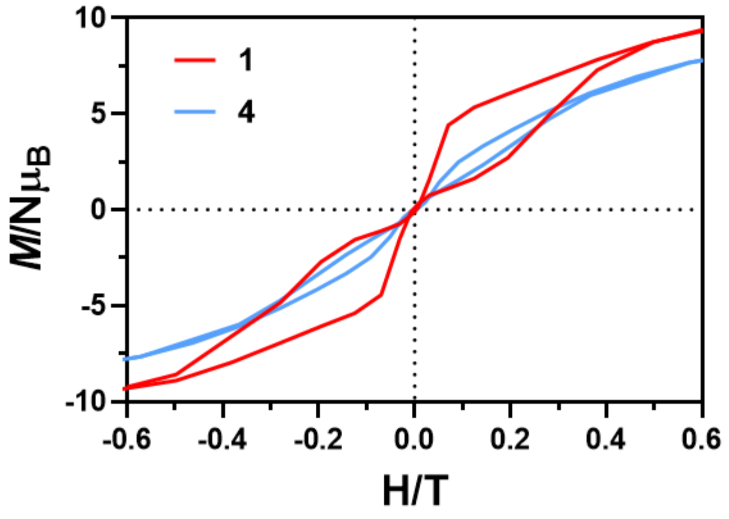

Figure S60.- Magnetic hysteresis loops for **1** and **4** for H values between -0.6 T and 0.6 T at 2 K. For **1**, the average sweep-rate in the 0.63 T to -0.63 T and -0.63 T to 0.63 T range was 61 Oe/s; for **4**, in the 0.57 T to -0.57 T and -0.57 T to 0.57 T range was 51 Oe/s.

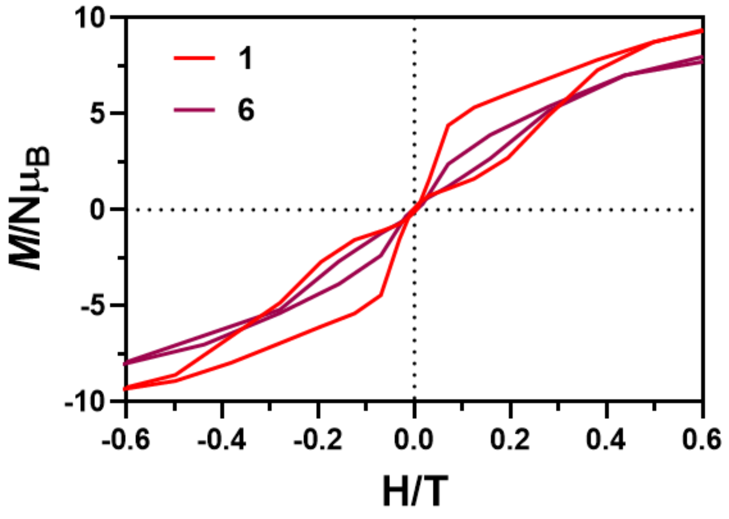

Figure S61.- Magnetic hysteresis loops for **1** and **6** for H values between -0.6 T and 0.6 T at 2 K. For **1**, the average sweep-rate in the 0.63 T to -0.63 T and -0.63 T to 0.63 T range was 61 Oe/s; for **6**, 77 Oe/s over the 0.44 to −0.63 T range and 88 Oe/s over the −0.44 to 0.63 T range.

S6. *Ab initio* calculations.

a) Results of SA-CASSCF(9,7)/SO-RASSI/SINGLE_ANISP calculations

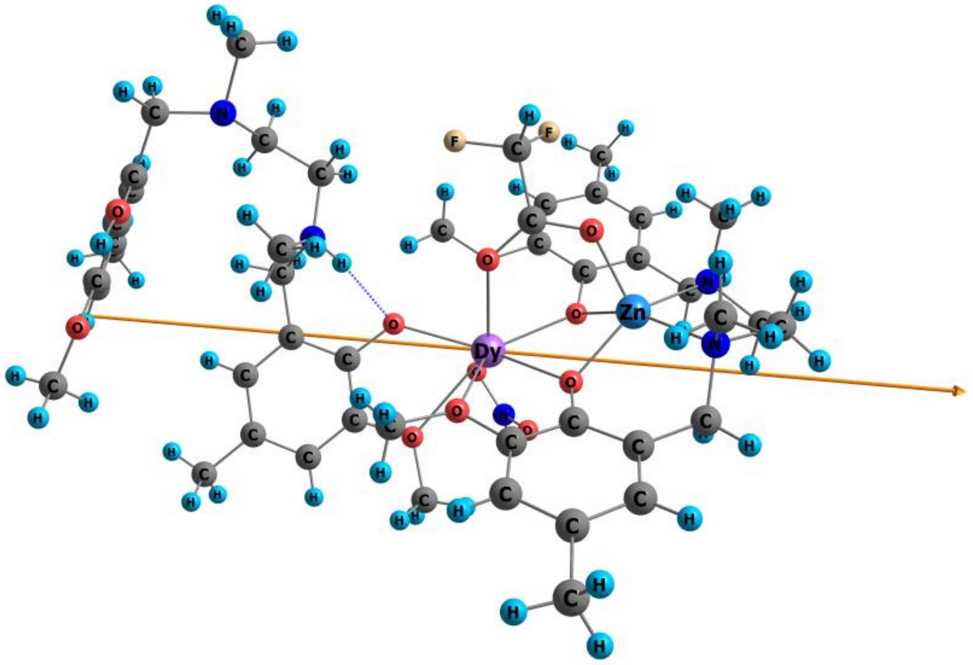

Figure S62.- Molecular structure of complex **1'** and the easy axis orientation (orange arrow line).

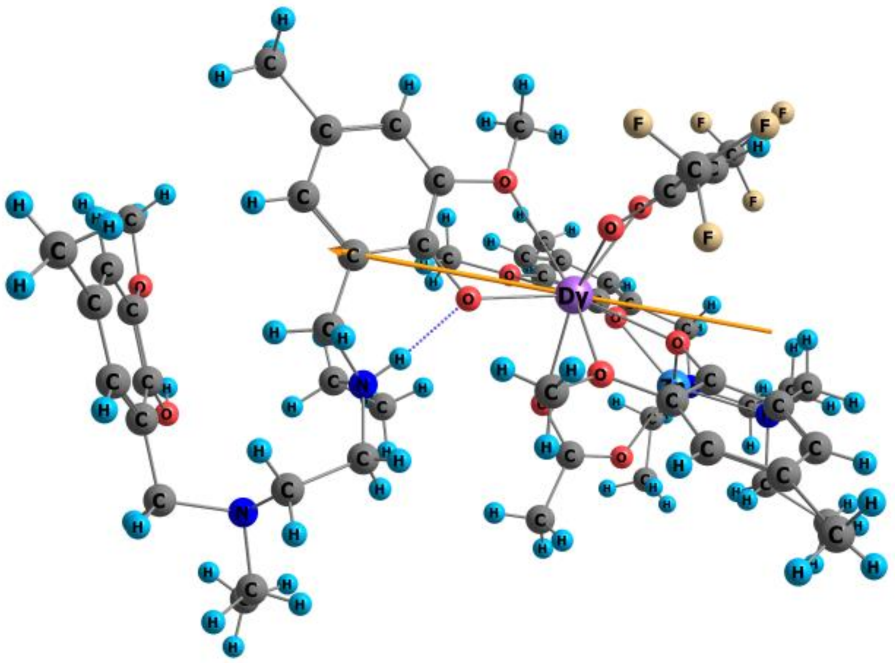

Figure S63.- Molecular structure of complex **2'** and the easy axis orientation (orange arrow line).

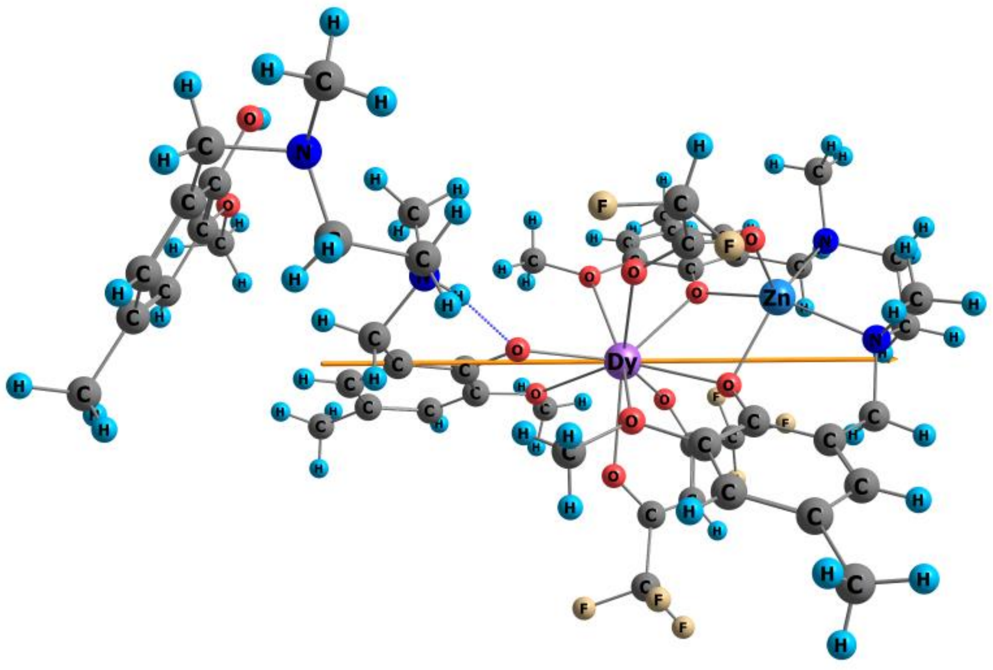

Figure S64.- Molecular structure of complex **3’** and the easy axis orientation (orange arrow line).

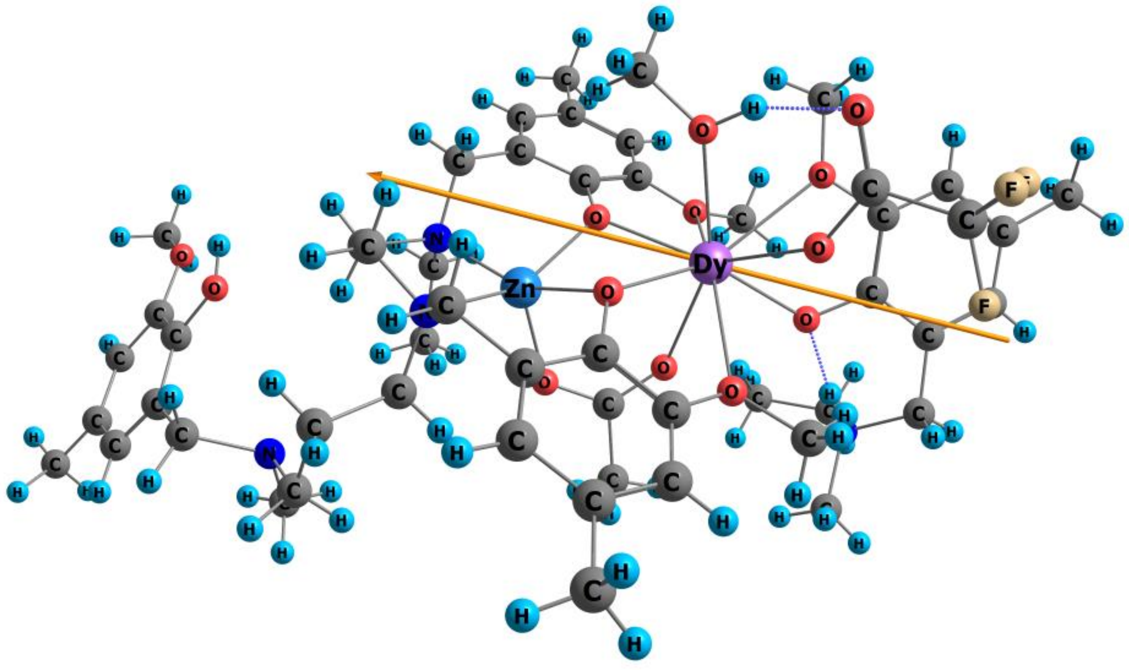

Figure S65.- Molecular structure of complex **4’** and the easy axis orientation (orange arrow line).

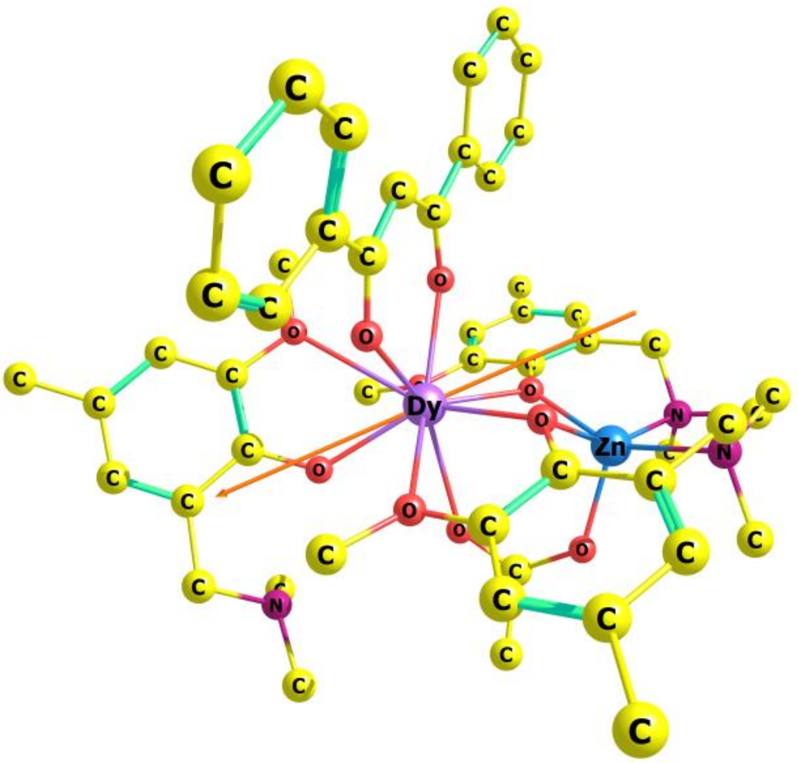

Figure S66.- Molecular structure of complex **5’** and the easy axis orientation (orange arrow line).

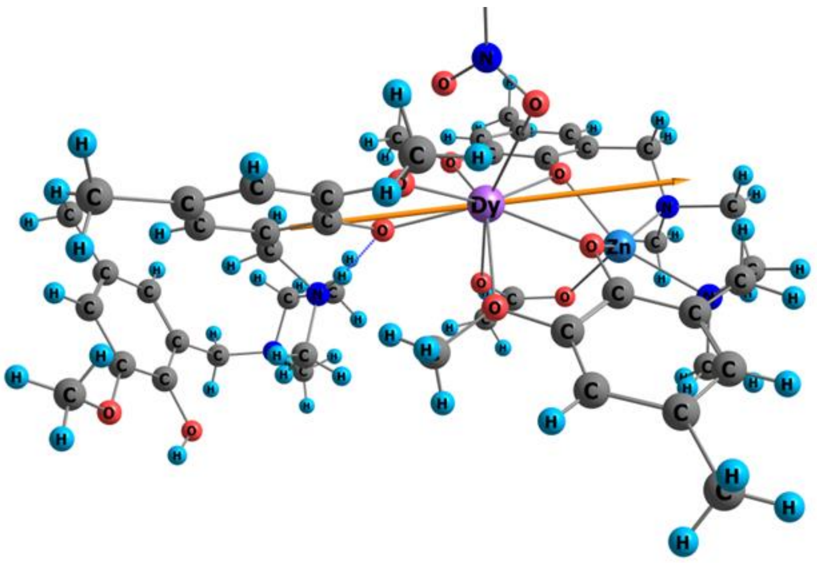

Figure S67.- Molecular structure of complex **6’** and the easy axis orientation (orange arrow line).

Table S16.- For **1'**, energy splitting of the ground $^6H_{15}$ multiplet**,** principal values of the *g* tensor for each Kramers' doublet and decomposition of the wave functions of each Kramers' doublet to the wave functions with definite J_z (z is the easy exes for the ground Kramers doublet).

Complex	KD	E, cm^{-1} (in K)	g_x	g_y	g_z	$\|J_z\|$ (contribution in %)
**1'**	1	0	0.0004	0.001	19.9	15/2 (99)
	2	210 (302)	0.054	0.059	17.16	13/2 (94.4)
	3	345 (496)	0.37	0.9	13.6	11/2 (71.8), 9/2 (14.5)
	4	410 (590)	3.91	4.15	12.61	3/2 (25.7), 7/2 (20.4), 9/2 (18.7), 1/2 (17.4), 5/2 (10.7)
	5	478 (688)	1.1	3.91	9.73	9/2 (38.4), 1/2 (19), 5/2 (15.4), 7/2 (12.9), 11/2 (11.1)
	6	572 (823)	1.48	2.36	13.52	7/2 (40.4), 9/2 (23.8), 5/2 (12.5)
	7	640 (921)	0.46	0.93	15.83	5/2 (41.6), 3/2 (30.9), 7/2 (18.2), 11/2 (12.3)
	8	813 (1170)	0.02	0.06	19.4	1/2 (47), 3/2 (31.3), 5/2 (14.3)

Table S17.- For **2’**, energy splitting of the ground $^6H_{15}$ multiplet**,** principal values of the *g* tensor for each Kramers’ doublet and decomposition of the wave functions of each Kramers’ doublet to the wave functions with definite J_z (z is the easy exes for the ground Kramers doublet).

Complex	KD	E, cm^{-1} (in K)	g_x	g_y	g_z	$\|J_z\|$ (contribution in %)
**2’**	1	0	0.008	0.012	19.85	15/2 (99.1)
	2	172 (247)	0.28	0.4	16.94	13/2 (92.7)
	3	247 (355)	2.67	5.6	13.38	11/2 (30.4), 5/2 (20), 9/2 (15.2), 7/2 (10)
	4	288 (414)	7.64	5.04	1.0	11/2 (35.4), 3/2 (20.8), 9/2 (15.1), 1/2 (12.7), 7/2 (12.1)
	5	370 (532)	1.53	2.88	10.94	9/2 (27.3), 1/2 (19.1), 11/2 (18.2), 7/2 (18), 5/2 (15.4)
	6	465 (669)	0.78	1.15	14.05	9/2 (33.1), 7/2 (22.4), 3/2 (16.5), 5/2 (10.5), 11/2 (11.2)
	7	533 (767)	0.1	0.27	16.1	5/2 (35.7), 7/2 (29.7), 3/2 (19.7)
	8	722 (1039)	0.016	0.03	19.41	1/2 (42.8), 3/2 (31.5), 5/2 (17)

Table S18.- For **3'**, energy splitting of the ground $^6H_{15}$ multiplet**,** principal values of the *g* tensor for each Kramers' doublet and decomposition of the wave functions of each Kramers' doublet to the wave functions with definite J_z (z is the easy exes for the ground Kramers doublet).

**Complex**	**KD**	**E, cm^{-1} (in K)**	**g_x**	**g_y**	**g_z**	**$\|J_z\|$ (contribution in %)**
**3'**	1	0	0.005	0.007	19.8	15/2 (99.2)
	2	198 (285)	0.14	0.16	17.02	13/2 (96)
	3	318 (458)	1.32	2.76	13.44	11/2 (69.7), 9/2 (12)
	4	360 (518)	8.58	6.74	3.59	9/2 (25.2), 3/2 (24.4), 1/2 (13.9), 11/2 (10.9)
	5	431 (620)	0.64	2.71	10.8	9/2 (28), 1/2 (24.5), 5/2 (18.8), 7/2 (16.6), 11/2 (10.1)
	6	519 (747)	0.51	1.41	12.33	9/2 (28.9), 3/2 (26.2), 7/2 (24.2)
	7	564 (811)	0.73	1.86	15.63	5/2 (39.3), 7/2 (30.8), 3/2 (13.7)
	8	726 (1044)	0.013	0.03	19.49	1/2 (42.1), 3/2 (32.7), 5/2 (17.9)

Table S19.- For **4'**, energy splitting of the ground $^6H_{15}$ multiplet**,** principal values of the *g* tensor for each Kramers' doublet and decomposition of the wave functions of each Kramers' doublet to the wave functions with definite J_z (z is the easy exes for the ground Kramers doublet).

Complex	KD	E, cm^{-1} (in K)	g_x	g_y	g_z	$\|J_z\|$ (contribution in %)
**4'**	1	0	0.002	0.003	19.75	15/2 (98.7)
	2	138 (198)	0.06	0.13	17.1	13/2 (85.3), 11/2 (10.6)
	3	209 (301)	1.62	2.74	13.52	11/2 (38.6), 9/2 (26.4), 5/2 (13.5)
	4	266 (383)	3.97	5.52	8.58	3/2 (24.2), 11/2 (22.2), 7/2 (18.7), 1/2 (15.2), 9/2 (10.4)
	5	345 (496)	1.24	2.58	10.74	9/2 (25.1), 1/2 (23.2), 5/2 (17.5), 7/2 (15.3), 11/2 (14.9)
	6	447 (643)	0.85	1.1	13.91	9/2 (29), 7/2 (23.9), 3/2 (17.3), 11/2 (10.8), 5/2 (10.7)
	7	520 (748)	0.24	0.37	16.13	5/2 (37.4), 7/2 (27), 3/2 (20.8)
	8	680 (978)	0.024	0.056	19.26	1/2 (46.6), 3/2 (32.5), 5/2 (14.9)

Table S20.- For **5'**, energy splitting of the ground $^6H_{15}$ multiplet**,** principal values of the *g* tensor for each Kramers' doublet and decomposition of the wave functions of each Kramers' doublet to the wave functions with definite J_z (z is the easy exes for the ground Kramers doublet).

**Complex**	**KD**	**E, cm^{-1} (in K)**	**g_x**	**g_y**	**g_z**	**$\|J_z\|$ (contribution in %)**
**5'**	1	0	0.24	0.61	19.19	15/2 (91.5)
	2	53 (76)	0.44	0.87	17.46	1/2 (24.6), 3/2 (20.6), 5/2 (15.9), 7/2 (11.3)
	3	116 (167)	0.79	3.45	12.80	13/2 (64.8)
	4	165 (237)	3.79	6.10	9.37	11/2 (23.4), 9/2 (21.0), 13/2 (14.3), 3/2 (13.4), 5/2 (12.6), 7/2 (10.6)
	5	239 (344)	1.84	2.47	11.47	11/2 (31.8), 1/2 (20.6), 7/2 (16.1), 9/2 (12.8)
	6	338 (486)	0.001	0.54	14.43	9/2 (24.6), 11/2 (20.8), 3/2 (20.7), 5/2 (18.2)
	7	406 (584)	0.25	0.40	17.60	7/2 (37.1), 9/2 (25.5), 5/2 (20.9)
	8	547 (787)	0.01	0.02	19.56	1/2 (35.8), 3/2 (30.3), 5/2 (20.4), 7/2 (9.4)

Table S21.- For **6'**, energy splitting of the ground $^6H_{15}$ multiplet**,** principal values of the *g* tensor for each Kramers' doublet and decomposition of the wave functions of each Kramers' doublet to the wave functions with definite J_z (z is the easy exes for the ground Kramers doublet).

Complex	KD	E, cm^{-1} (in K)	g_x	g_y	g_z	$\|J_z\|$ (contribution in %)
6'	1	0	0.0046	0.0054	19.8	15/2 (99.6)
	2	199 (286)	0.081	0.132	17.2	13/2 (88.3), 11/2 (9.5)
	3	326 (469)	1.15	2.02	13.2	11/2 (46.7), 9/2 (23.5)
	4	373 (537)	0.35	3.5	12.26	1/2 (26), 11/2 (20.2), 7/2 (18.9), 3/2 (17.6), 5/2 (10.2)
	5	453 (652)	7.19	6.6	5.12	9/2 (34.7), 7/2 (20.6), 5/2 (17.1), 11/2 (14.3)
	6	542 (780)	2.01	2.52	14.35	7/2 (32.2), 9/2 (31.2), 5/2 (17.6)
	7	629 (905)	0.43	0.72	15.88	5/2 (37.7), 3/2 (28.9), 7/2 (23.5)
	8	750 (1079)	0.07	0.2	18.72	1/2 (52.1), 3/2 (31.1), 5/2 (11.2)

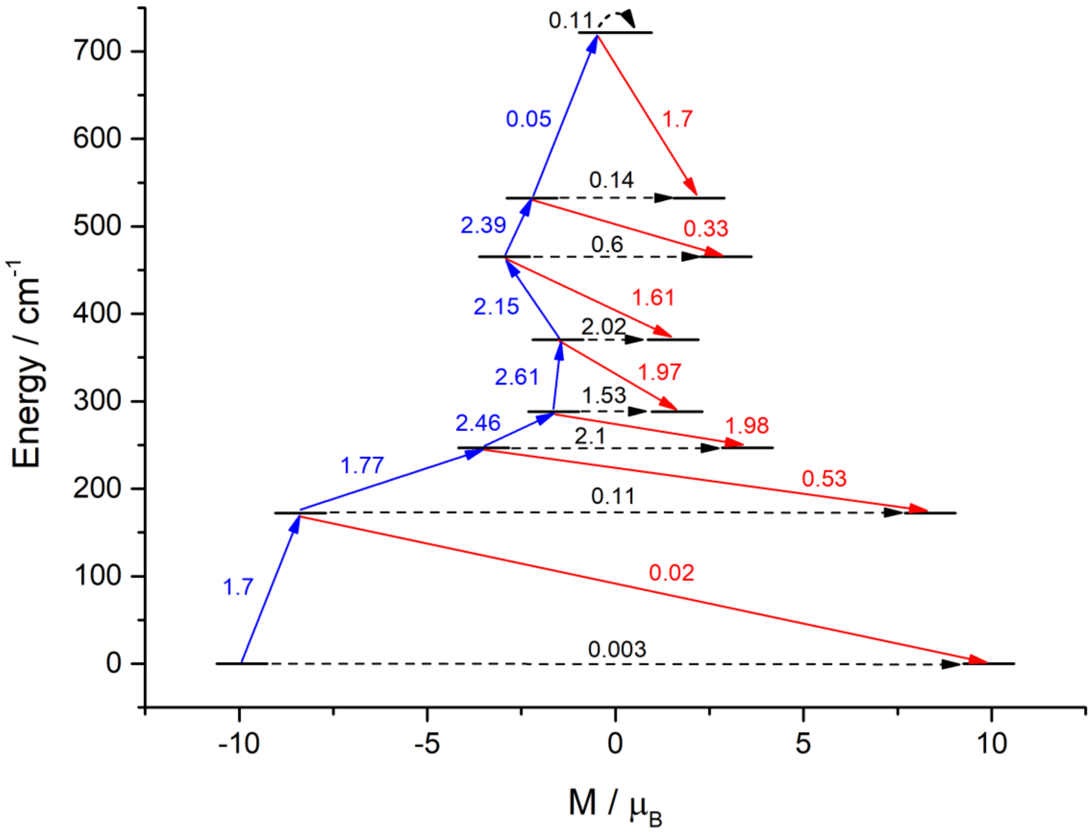

Figure S68.- Magnetization blocking barrier for **2’**. The exchange states are arranged according to the values of their magnetic moments. The arrows show the connected exchange states, and the numbers at each of them stand for the corresponding matrix element of the transition magnetic moment.

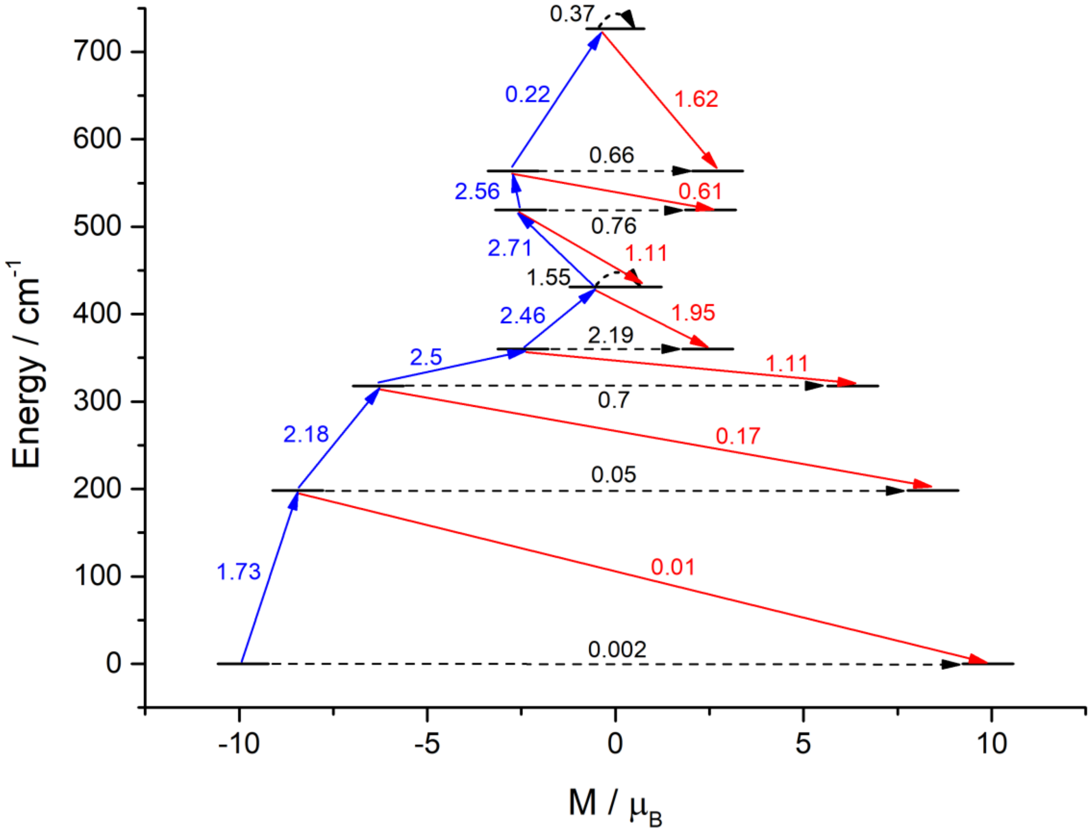

Figure S69.- Magnetization blocking barrier for **3’**. The exchange states are arranged according to the values of their magnetic moments. The arrows show the connected exchange states, and the numbers at each of them stand for the corresponding matrix element of the transition magnetic moment.

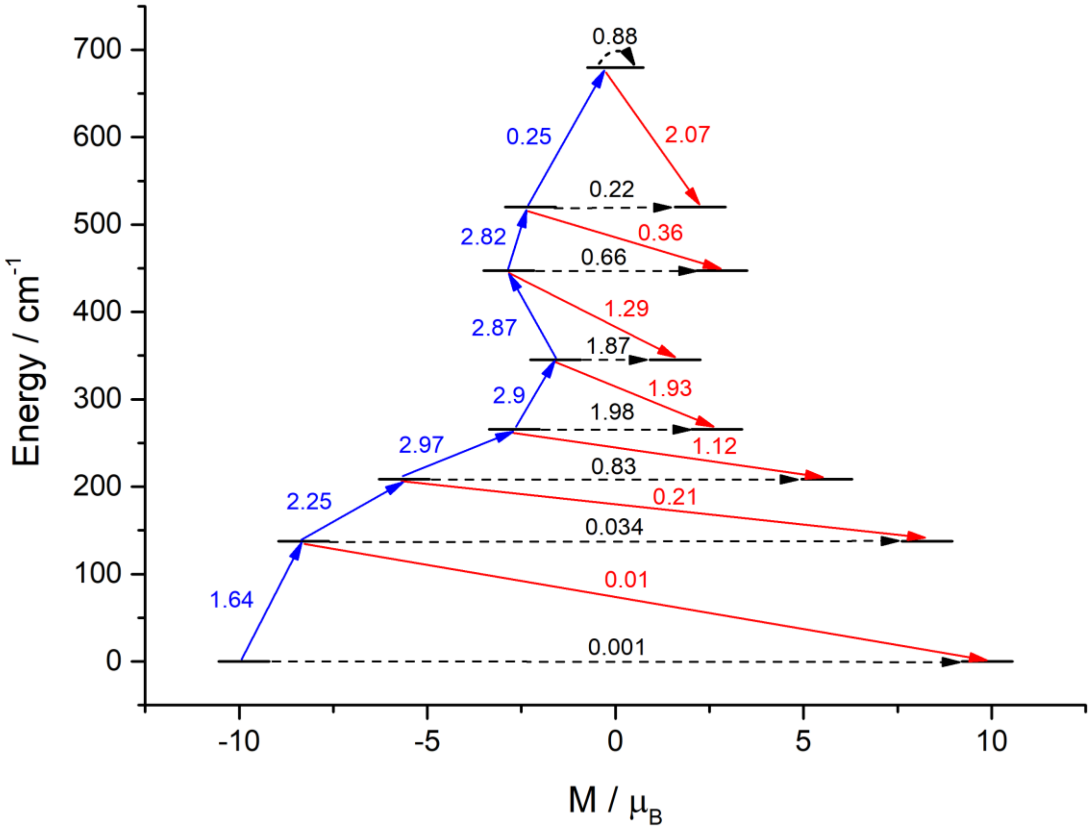

Figure S70.- Magnetization blocking barrier for **4'**. The exchange states are arranged according to the values of their magnetic moments. The arrows show the connected exchange states, and the numbers at each of them stand for the corresponding matrix element of the transition magnetic moment.

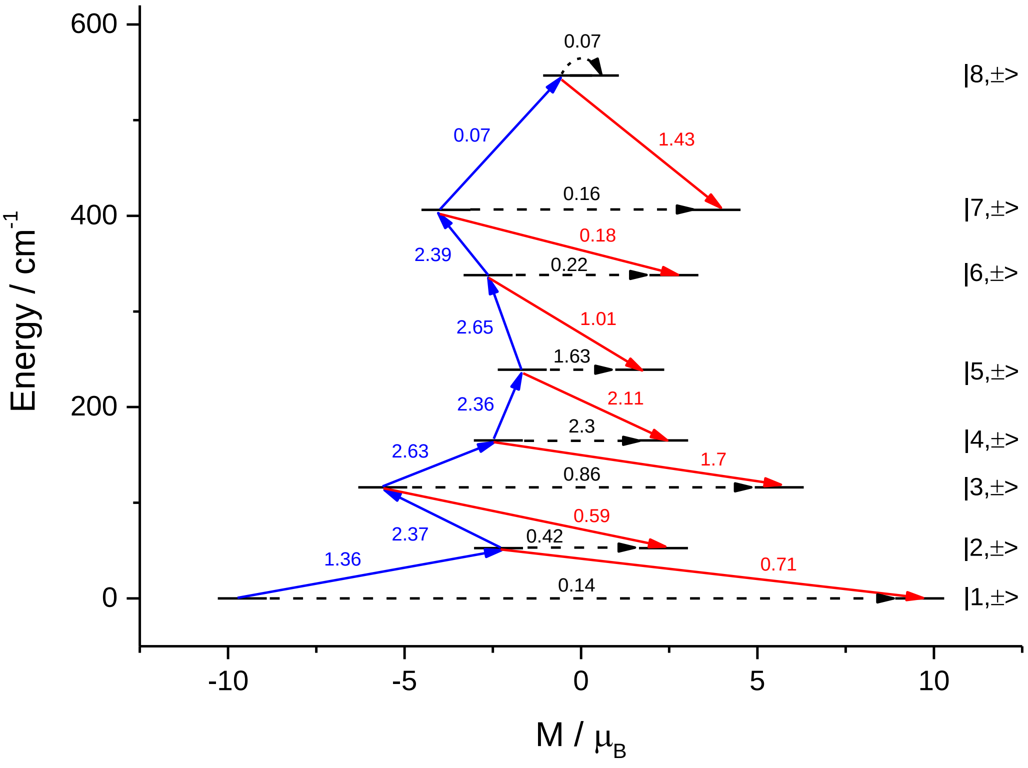

Figure S71.- Magnetization blocking barrier for **5'**. The exchange states are arranged according to the values of their magnetic moments. The arrows show the connected exchange states, and the numbers at each of them stand for the corresponding matrix element of the transition magnetic moment.

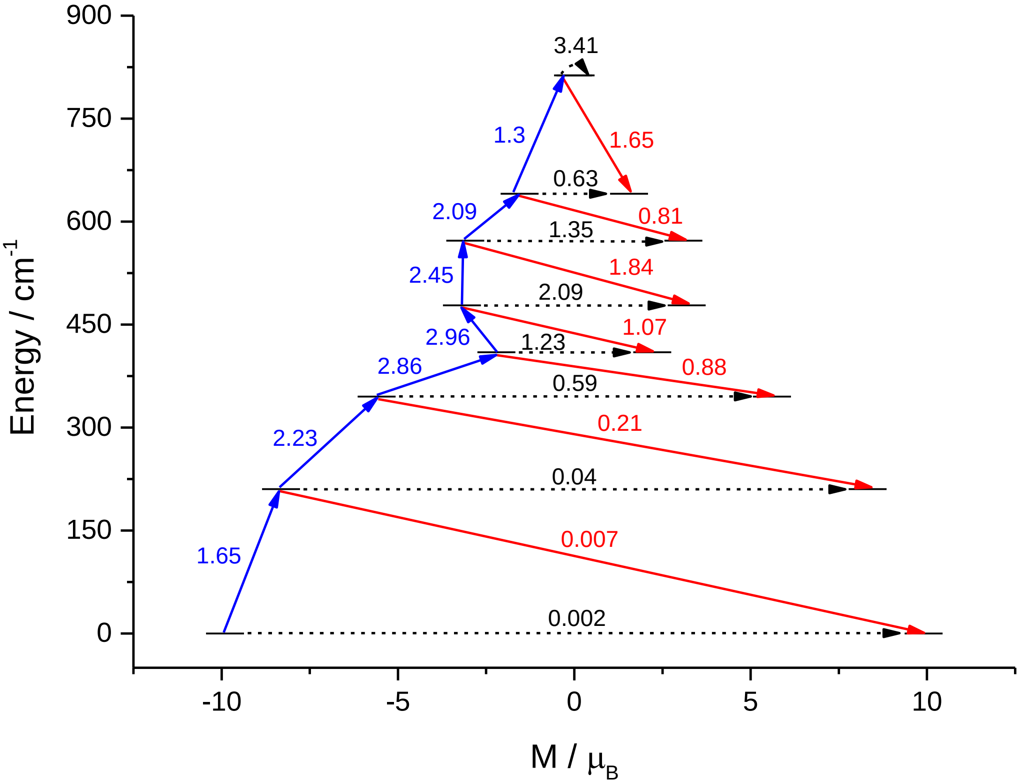

Figure S72.- Magnetization blocking barrier for **6'**. The exchange states are arranged according to the values of their magnetic moments. The arrows show the connected exchange states, and the numbers at each of them stand for the corresponding matrix element of the transition magnetic moment.

b) Calculations of tunnelling relaxation times and effective demagnetization barriers in complexes 1 – 6 using the model proposed by D. Aravena.

D. Aravena proposed a simple model of the Orbach relaxation consisting of thermally assisted tunneling in which a fast thermal excitation step followed by slower excited state tunneling ($\tau_{QT,i}^{calc} = \mathbf{1/k_{QT,i}}$), which controls the demagnetization rate.[2]. Under this regime, each Kramers' doublet has a particular demagnetization rate following:[3]

$$k_i(T) \propto \frac{\exp(-\frac{E_i}{k_B T})}{Z} k_{QT,i},$$

The effective demagnetization barrier is defined as

$$U_{eff} = \sum_{i=1}^{M} \frac{k_i(T)}{N_k} E_{i,}$$

where M is the number of Kramers' doublets (8 for Dy^{III}) and $N_k = \sum_{i=1}^{M} k_i(T)$.

All calculations were performed using UandTau program[4] for each unique center in the unit cells of compounds **1** – **6**, namely, 1 centre for **6**, 2 centres for **1**, **3**, **4** and **5**, and 6 centres for **2**. The model realized in the UandTau program allows to take into account the influence of crystal packing on the U_{eff} and τ_{QT} through the dipolar interactions between the metal centers. The g-vectors were reoriented for each center to match the crystal geometry of every magnetic center. The Tables S22-S23 present the calculated tunnelling relaxation times for compounds **1** – **6**. The relaxation contributions of each Kramers doublet are presented in the Figures S73-S78. The calculated demagnetization barriers are shown in Table S24 with the experimental values taken from the Table 1 and 2 (main text).

Table S22. Tunnelling relaxation times, $\tau_{QT,i}^{calc}$, for each of the eight lower Kramers doublets (KD1 – KD8) for each unique Dy^{III} centre in the unit cell of compounds **1** and **2** calculated by the UandTau program using the values of KDs energies, g-tensor components, and g-vectors obtained from SA-CASSCF(9,7)/SO-RASSI/SINGLE-ANISO calculations. The states giving the main contributions to demagnetization are marked in bold.

**KD**	$\mathbf{Log}(\tau_{QT,i}^{calc}, \boldsymbol{s})$							
i	**1′**		**2′**					
	a	b	a	b	c	d	e	f
			$\mathbf{Log}(\tau_{QT,1}^{exp}) = \mathbf{-2.77 \pm 0.13}$					
**1**	-0.975	-2.494	-5.827	-5.044	-4.920	-3.640	-4.646	-2.971
**2**	-4.515	-5.930	-8.812	-7.941	-8.066	-6.510	-7.451	-6.006
**3**	**-6.804**	**-8.147**	**-9.650**	**-8.736**	**-9.548**	**-8.394**	**-9.160**	**-7.711**
**4**	**-6.226**	**-8.288**	**-11.647**	**-10.469**	**-10.462**	**-9.621**	**-10.376**	**-8.413**
**5**	**-6.212**	**-8.891**	-10.464	-8.950	-8.239	-8.982	-10.136	-6.140
**6**	**-6.818**	**-9.027**	-9.996	-8.850	-8.361	-8.119	-9.162	-6.217
**7**	-5.361	-7.758	-8.449	-7.836	-6.699	-6.920	-7.910	-4.717
**8**	-1.958	-5.152	-6.362	-5.696	-2.742	-5.033	-5.803	-1.682

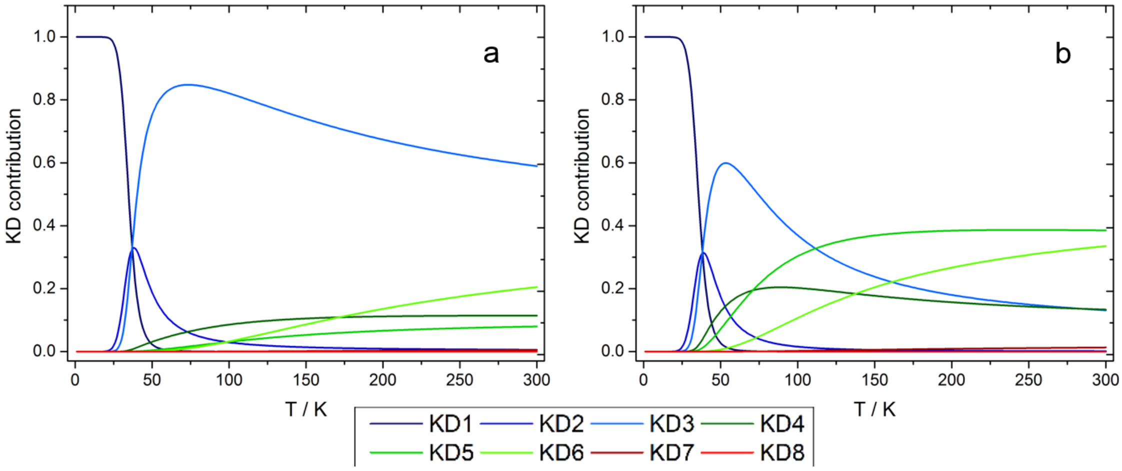

Figure S73. Relaxation contributions from each lower Kramers doublets (coloured lines, left *y*-axis) as a function of temperature for two centres in the unit cell of compound **1**.

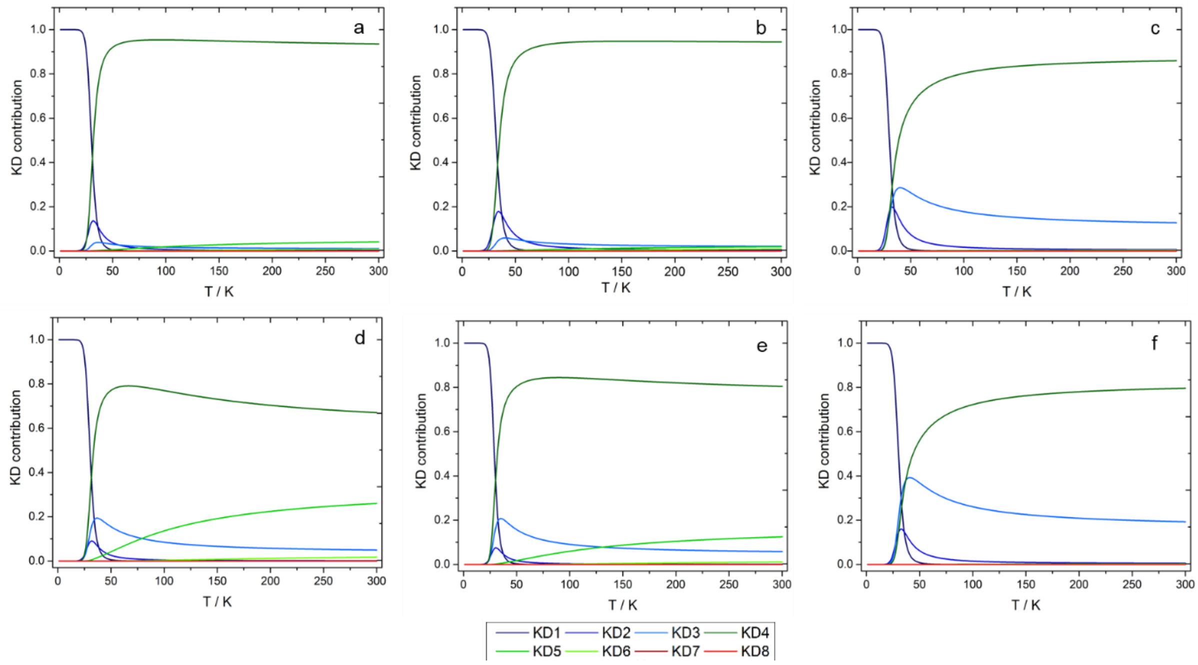

Figure S74.- Relaxation contributions from lower Kramers doublets (coloured lines, left *y*-axis) and the predicted effective barrier (dashed line, right *y*-axis) as a function of temperature for six centres in the unit cell of compound **2**.

Table S23. Tunnelling relaxation times, $\tau_{QT,i}^{calc}$, for every of lower Kramers doublets (KD1 – KD8) for each unique Dy^{III} centre in the unit cell of compounds **3 – 6** calculated by the UandTau program using the values of KDs energies, g-tensor components, and g-vectors obtained from RASSCF(9,7)/SO-RASSI/SINGLE-ANISO calculations. The states gjving the main contributions to demagnetization are marked in bold.

**KD**	**Log($\tau_{QT,i}^{calc}$, s)**						
i	**3**		**4**		**5**		**6**
	a	b	a	b	a	b	A
**1**			**Log($\tau_{QT,1}^{exp}$) = −2.62 ± 0.1**				
	-1.754	-2.452	-2.406	-2.369	-6.255	-6.741	-4.207
**2**	-4.971	-5.197	-5.702	-5.657	-5.620	-6.392	-8.097
**3**	**-7.357**	**-7.724**	**-8.955**	**-7.931**	**-7.905**	**-8.003**	**-9.994**
**4**	**-7.894**	**-8.752**	**-8.559**	**-8.182**	**-7.110**	**-8.203**	**-8.666**
**5**	**-7.447**	-5.979	**-8.524**	-6.204	-6.897	**-8.342**	**-9.609**
**6**	-5.308	-6.303	-7.826	-6.094	-5.774	-7.102	-11.901
**7**	-5.849	-6.610	-7.045	-4.750	-5.316	-6.608	-10.855
**8**	-3.989	-1.611	-5.617	-0.289	-2.116	-3.800	-9.075

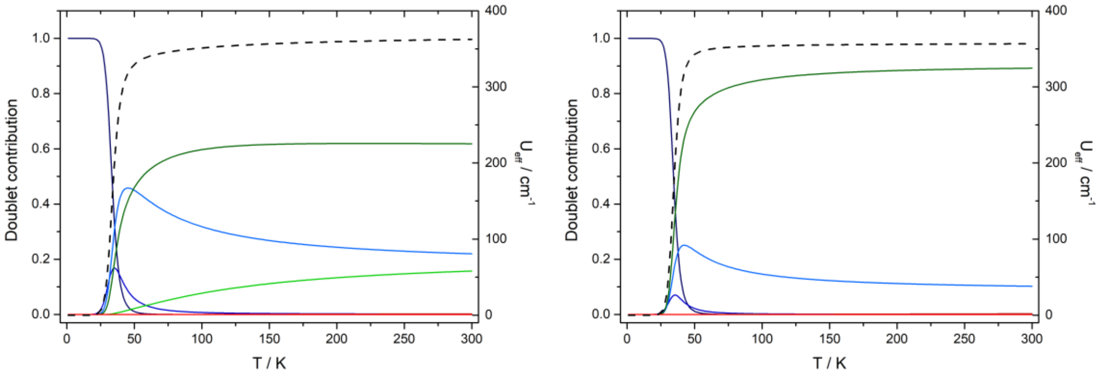

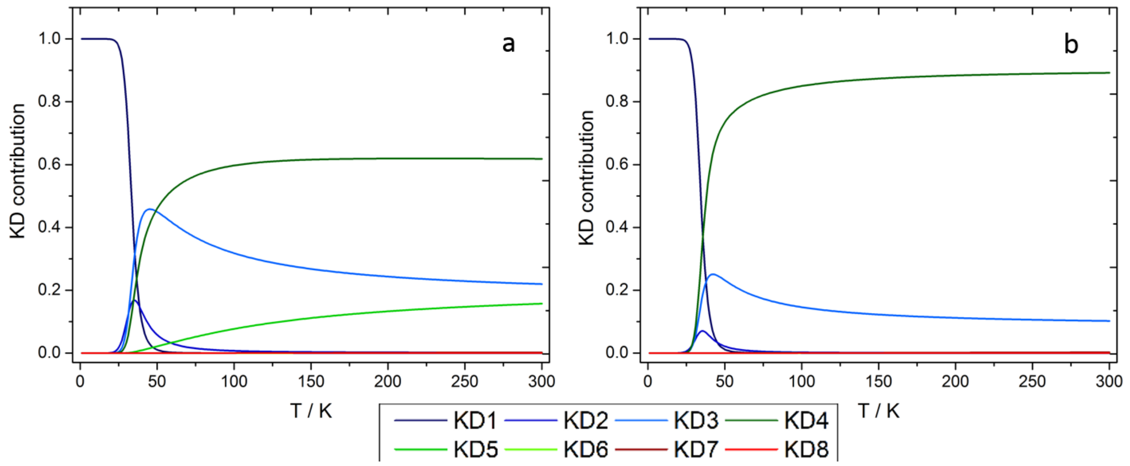

Figure S75. Relaxation contributions from eight Kramers doublets (coloured lines, left *y*-axis) as a function of temperature for two types of Dy^{III} centres in the unit cell of compound **3**.

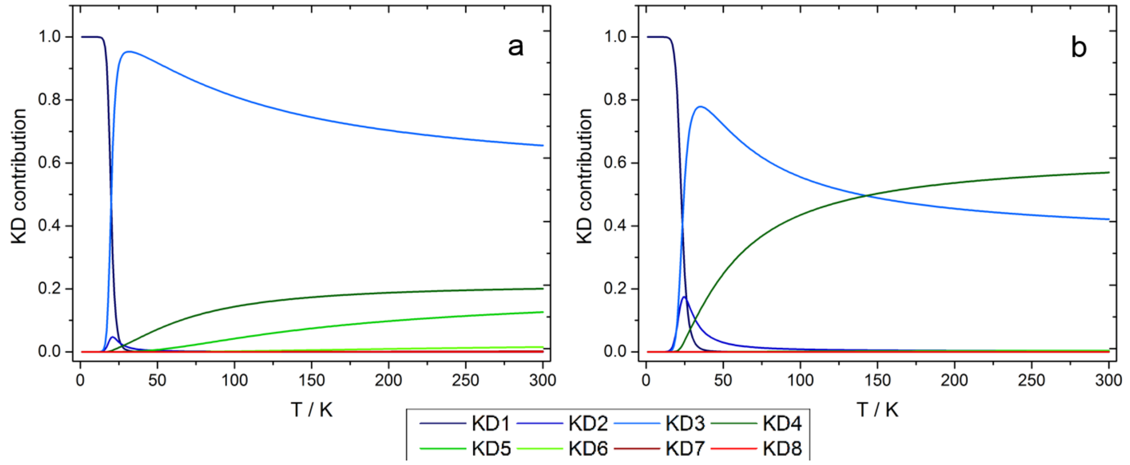

Figure S76. Relaxation contributions from eight Kramers doublets (coloured lines, left *y*-axis) and the predicted effective barrier (dashed line, right *y*-axis) as a function of temperature for two centres in the unit cell of compound **4**.

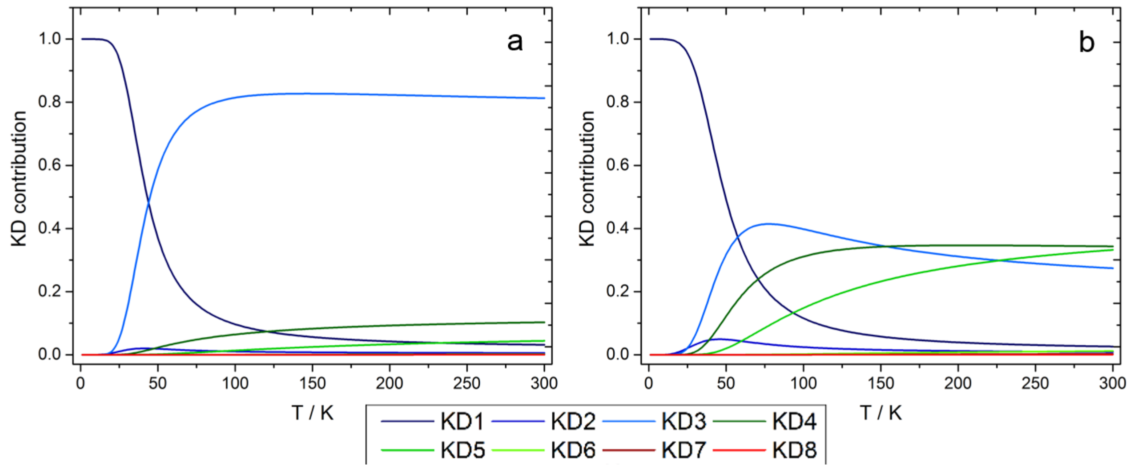

Figure S77.- Relaxation contributions from eight Kramers doublets (coloured lines, left *y*-axis) as a function of temperature for two centres in the unit cell of compound **5**.

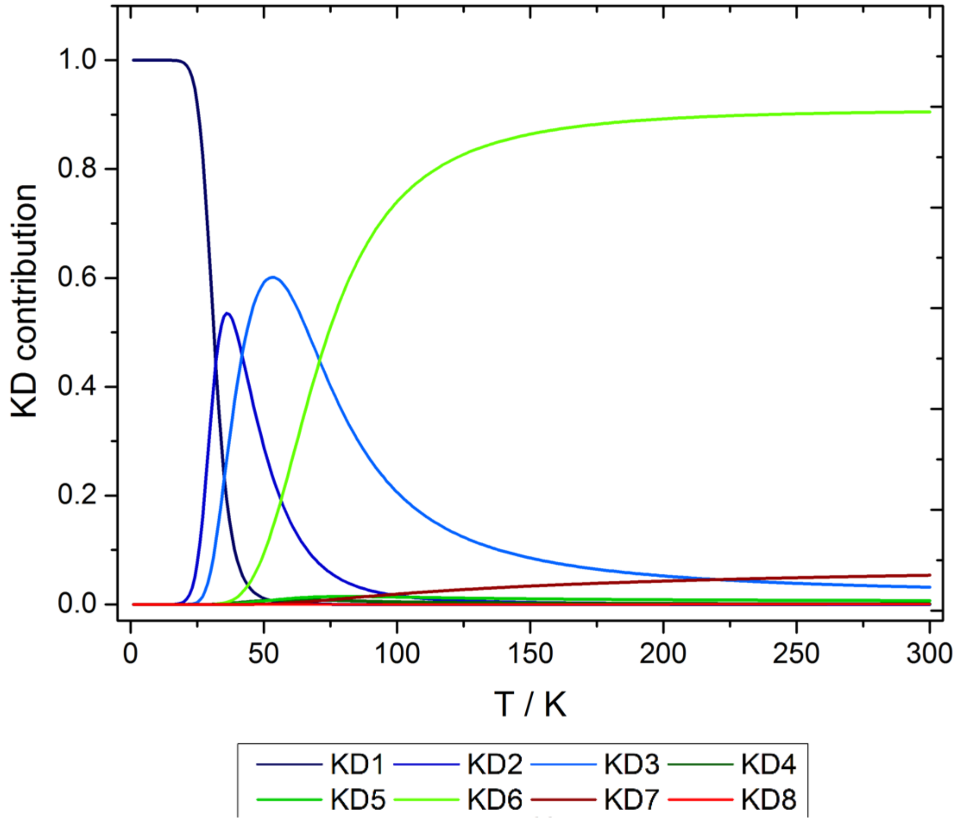

Figure S78. Relaxation contributions from the ground and excited Kramers doublets (coloured lines, left *y*-axis) as a function of temperature for a single centre in the unit cell of compound **6**.

Table S24. The effective magnetization relaxation barriers, U_{eff}^{calc} (cm^{-1}), for each centre in the unit cells of compounds **1-6,** calculated assuming a simple mechanism consisting of thermally activated tunneling in which a fast thermal excitation step is followed by a slower excited state tunneling, as well as the calculated energies of the lower excited Kramers doublets (E_2, E_3) and the experimental effective magnetization relaxation barriers (U_{Orbach}^{exp}/k_B) and values of τ_{QTM}^{exp}. The calculations were performed using the UandTau program and the results of SA-CASSCF(9,7)/SO-RASSI/SINGLE-ANISO calculations.

Compound		U_{eff}^{calc}, cm^{-1}	$\langle U_{eff}^{calc} \rangle$, cm^{-1}	$\langle U_{eff}^{calc} \rangle / k_B$, K	$\frac{E_2}{k_B}$, K	$\frac{E_3}{k_B}$, K	$\tau_{QT,1}^{calc}$, s	$(T_i - T_f)_{exp}$	U_{Orbach}^{exp}/k_B, K	τ_{QTM}^{exp}, s
**1′**	a	410	447 ± 37	**643 ± 53** (~E_4,E_5/k_B)	302	496	3.2E-3, 1.1E-1	15.8 – 32.5	**370±30**	–
	b	485								
**2′**	a	293	293 ± 15	**421 ± 22** (~E_3/k_B)	247	414	1.5E-6 ÷ **1.1E-3**	4.0 – 17.7	–	**1.5±0.2 E-3**
	b	290								
	c	283								
	d	298								
	e	311								
	f	280								
**3′**	a	362	360 ± 3	**517 ± 4** (~E_4/k_B)	**285**	**458**	3.6E-3, 1.8E-2	12.0 – 25.7	**329±11**	–
	b	357								
**4′**	a	242	242	**348** (~E_3,E_4/k_B)	198	301	**4.1E-3**	2.0 – 21.3	**232±7**	**2.4±0.3 E-3**
	b	242								
**5′**	a	123	148 ± 25	**213 ± 36** (~E_3,E_4/k_B)	76	167	1.8E-7, 5.5E-7	–	–	–
	b	174								
**6′**	a	539	539	776 (~E_6/k_B)	286	469	6.2E-5	13.2 – 27.6	**330±19**	–

The τ_{QTM} values were evaluated experimentally only for compounds **2** and **4**. For **4**, the experimental τ_{QTM} value was found to be $(2.4\pm0.3)\cdot10^{-3}$ s. Fortunately, in the case of **4**, the two types of Dy^{III} complexes present in the unit cell have very close theoretically calculated values of $\tau_{QT,1}^{calc} \cong 4.1\cdot10^{-3}$ s, which in turn is very close to the value, obtained from the analysis of the magnetization relaxation kinetics. This agreement confirms the theoretical model of quantum tunneling based on the spin-spin dipole-dipole interaction.[2,4] Note that the interactions of the selected Dy cation with pseudo-spin ½ with a large number of surrounding Dy ions were taken into account when calculating the relaxation parameters.

In the case of compound **2**, the situation is much more complicated: the unit cell contains 6 types of Dy complexes with significantly different $\tau_{QT,1}^{calc}$ values, lying in the range of $1.5\cdot10^{-6}$ – $1.1\cdot10^{-3}$ s (Table S22). It turned out that the experimentally estimated value τ_{QTM}, equal to $(1.5\pm0.2)\cdot10^{-3}$ s, is close to the maximum calculated value of $\tau_{QT,1}^{calc}$ equal to $1.1\cdot10^{-3}$ s. Most likely, in case of compound **2**, the majority of Dy complexes (possibly 5 of 6), which have very short tunneling times in the ground state, do not exhibit SMM properties. In this case, magnetization relaxation is experimentally detected only for complexes with the longest τ_{QTM}, and the theoretical estimate again agrees well with the value obtained from the analysis of the magnetization relaxation.

c) Calculated crystal field parameters for compounds 1 – 6.

The crystal field (CF) parameters for the ground atomic multiplet (J = 15/2) were computed employing the crystal field Hamiltonian

$$\hat{H}_{CF} = \sum_{k} \sum_{q=-k}^{k} B_q^k \tilde{O}_q^k,$$

where $\tilde{O}_q^k$ are the Stevens operators and B_q^k are the CF parameters. All parameters are calculated in the coordinate system with the main magnetic axes of the ground pseudo-doublet state.

The ***higher values*** of axial B_q^k parameters with q = 0 (f.e. B_0^2 and B_0^4) lead to ***stronger energy level splitting*** for the metal ion that results in better SMM properties. Otherwise, the significant values of non-axial parameters (q ≠ 0) result in the split states having admixture of the states with different M_S that leads to noticeable QTM.

Table S25. Calculated crystal field parameters B_q^k (k = 2 and 4; -k ≤ q ≤ k) and their weights in the crystal field splitting for compounds **1'– 3'**.

k	q	**1'**		**2'**		**3'**	
		B_q^k	Weight,%	B_q^k	Weight,%	B_q^k	Weight,%
2	2	*1.63*	*10.9*	*2.45*	*17.4*	*1.66*	*12.3*
2	1	0.55	3.7	0.72	5.1	-0.69	5.15
2	0	**-3.66**	**24.5**	**-2.9**	**20.5**	**-3.21**	**23.9**
2	-1	-0.16	1.1	0.83e-01	0.58	-0.61e-01	0.45
2	-2	*-1.18*	*7.92*	-0.74e-01	0.53	0.27	2.04
4	4	0.26e-02	3.13	0.36e-03	0.46	0.13e-02	1.8
4	3	-0.34e-02	4.14	-0.53e-02	6.77	-0.48e-02	6.43
4	2	-0.23e-02	2.85	-0.52e-02	6.66	-0.46e-02	6.27
4	1	0.38e-03	0.46	-0.15e-02	1.91	0.15e-02	2.04
4	0	-0.27e-02	3.34	-0.24e-02	3.06	-0.3e-02	3.98
4	-1	0.16e-02	2.0	-0.24e-02	3.11	0.18e-02	2.43
4	-2	0.53e-02	6.46	-0.22e-02	2.84	-0.19e-02	2.5
4	-3	0.36e-02	4.33	0.97e-03	1.24	0.18e-02	2.49
4	-4	0.47e-03	0.57	-0.27e-02	3.44	-0.62e-03	0.84

Table S26. Calculated crystal field parameters B_q^k (k = 2,4; -k ≤ q ≤ k) and their weights in the crystal field splitting for compounds **4'-6'**.

		**4'**		**5'**		**6'**	
k	q	B_q^k	Weight,%	B_q^k	Weight,%	B_q^k	Weight,%
2	2	0.46	3.17	*2.71*	*18.8*	*1.75*	*11.45*
2	1	0.88	6.09	0.8	5.57	0.91	5.97
2	0	**-2.85**	**19.6**	**-1.7**	**11.8**	**-3.36**	**22.0**
2	-1	-0.49	3.39	-0.39	2.75	-0.25	1.63
2	-2	*-2.25*	*15.5*	-0.82	5.67	0.46	3.03
4	4	-0.13e-02	1.61	-0.14e-03	0.18	-0.2e-02	2.38
4	3	0.17e-02	2.14	-0.58e-02	7.3	-0.39e-02	4.7
4	2	-0.48e-02	5.99	-0.47e-02	5.96	-0.57e-02	6.73
4	1	-0.29e-02	3.62	-0.21e-02	2.64	-0.79e-03	0.95
4	0	-0.99e-03	1.24	-0.28e-02	3.58	-0.31e-02	3.72
4	-1	-0.17e-02	2.1	0.98e-03	1.24	-0.98e-03	1.18
4	-2	0.26e-02	3.26	0.18e-02	2.28	-0.21e-02	2.51
4	-3	0.51e-02	6.38	0.38e-02	4.87	0.18e-02	2.15
4	-4	0.19e-02	2.37	0.18e-03	0.23	-0.44e-02	5.22

Tables S25 and S26 present the crystal field parameters and their weights in the crystal field splitting, calculated for complexes **1′** – **6′** based on the results of SA-CASSCF(9,7)/SO-RASSI calculations. It can be seen that the CF parameters B_q^2 are the most interesting and important, since they account for more than 60% of the splitting of energy levels. Therefore, the most indicative parameters B_0^2 are summarized separately in Table S27. Based on the values of the parameters B_0^2 and their weights, the following axiality series can be obtained **1′** > **3′** ≈ **6′** > **2′** ≈ **4′** >> **5′**. Thus, compounds **1**, **3**, and **6** have higher axiality than others, with complex **1′** having the most axial structure, which is due, in particular, to the shortest average Dy-$O_{phenoxido(1,3,7)}$ distances (Figure 2, Tables S3-S9). Moreover, the total weight of the $B_{q\neq 0}^2$ parameters is lower for **1′**, **3′**, and **6′** compared to the other compounds, which reflects the stronger single-component nature of their lowest three pseudo-spin states (Tables S16, S18, and S21).

Table S27. Calculated crystal field parameters B_0^2 and their weights in the crystal field splitting for compounds **1′** - **6′**.

k	q	**1′**		**2′**		**3′**	
		B_q^k	Weight,%	B_q^k	Weight,%	B_q^k	Weight,%
2	0	**-3.66**	**24.5**	**-2.9**	**20.5**	**-3.21**	**23.9**
		**4′**		**5′**		**6′**	
k	q	B_q^k	Weight,%	B_q^k	Weight,%	B_q^k	Weight,%
2	0	**-2.85**	**19.6**	**-1.7**	**11.8**	**-3.36**	**22.0**

The most illustrative case is complex **5′,** which has the highest weight of the B_2^2 parameter, while the B_0^2 weight is the smallest one. Thus, this complex has the smallest axiality, which correlates with the small energy splitting of the energy levels and the multi-component nature of the wave functions of the ground and excited pseudo-spin states (Table S20). According to the XRD results, the Dy-$O_{phenoxido(1, 3, 7)}$ distances are the longest, and the Dy1-O1C and Dy1-O2C equatorial distances (Table S9) are the shortest among all compounds. Thus, the XRD results and CF analysis confirm the worst axiality of compound **5**.

It is obvious that not only the molecular structure of the Dy complex itself influences the SMM properties of the studied compounds. The crystal structure as a whole has an effect on the relaxation of magnetization. Compound **2** is the most interesting and illustrative

for demonstrating both effects. Compared to **1′**, **3′**, and **6′**, for **2′** the parameter B_0^2 and its weight are somewhat lower, and the weight of the parameter B_2^2 is higher, which indicates a lower axiality of complexes **2′**, manifested in a somewhat lower splitting of the levels. However, the most significant difference between compound **2** and the other studied compounds is the presence in the crystal cell of not two, as for the majority of the studied compounds, but six dysprosium centers. Moreover, the magnetization tunneling times calculated for these centers, particularly for their ground Kramers doublets, differ by almost three orders of magnitude. This difference is due to significant variations in the dipole-dipole interaction between the magnetic moments of the different types of dysprosium centers and the magnetic moments of their surroundings. It should be noted that it is possible to qualitatively predict the effect of substitution of ligands in the coordination sphere on the axiality of the molecular structure, but it is practically impossible to predict the effect of such a substitution on the crystal structure as a whole. The case of compound **2** is a clear example of this.

S7. Photoluminescence properties.

Besides the room-temperature measurements described in the manuscript, further low-temperature analysis of the photoluminescent behaviour was carried out for all compounds. Decreasing the temperature of the samples enhances the ligand fluorescent intensity, which leads to a relative increase of their bands compared to the bands associated with intraionic transitions, although the latter still dominate in the spectra of compounds **2** and **3** (Figures S72-S73). The observed increase in the signals intensity is derived from the lower molecular vibrations of the ligands resulting in a decrease in the non-radiative component and more efficient fluorescence at low temperatures.[5] This effect is particularly relevant for compound **5**, in which at low temperature the ligand-centred band becomes dominant in the excitation spectrum, showing an additional broad band at 340 nm related to excitation of dbm chelating ligands.[6,7] Accordingly, the emission spectra also experience important changes with temperature, as the weak Dy^{III}-centred emission bands at room temperature become much more intense and their structure resolved at low temperatures. The change is especially appreciable for compounds **2** and **5**, because the Dy-centred characteristic signals hardly distinguished from background (the wide band attributed to ligand's fluorescence) at room temperature result in well-defined narrow bands at low temperature (see Figures S72 and S74). Taking advantage of those temperature-dependent significant changes, we studied their detailed thermometric properties. Starting by compound **2**, the Dy^{III}-centred integrated emission at 576 nm describes U-shaped curve with a deep intensity decrease from 25 to 150 K and a subsequent slow recovery up to room temperature aided by the increase of ligand's fluorescence at high temperatures (Figure S89). Taking into account this second signal describes a very similar behaviour with temperature and that its contribution to the Dy^{III}-centred emission cannot be removed, the Dy^{III}-centred emission evolution with

temperature must be considered as the most representative thermometric parameter. The best fitting results of the U-shaped curve are achieved with the following 3rd order polynomial expression:

$$I_{576} = 1.004e^{7} - 93359T + 431.57T^{2} - 0.56963T^{3}$$

On the other hand, the temperature evolution of compound **5** is more interesting because the main Dy^{III}-centred integrated emission (λ_{em} = 576 nm), showing pseudo-exponential decay with increasing temperature, differs from the slow proportional decrease shown by the ligands' integrated signal covering the λ_{em} = 350-465 nm range and maximum at 450 nm, in such a way that a ratiometric I_{576}/I_{450} signal offers a self-referencing reliable parameter. The plot of I_{576}/I_{450} vs T displays an almost linear curve that is best fitted with the following expression (Figure S90):

$$I_{576/450} = 0.30672 + 0.0034195T$$

From the previous plot, the maximum relative sensitivity (S_m) may be estimated to be of 1.6% K^{-1} at 110 K, which is far below the activity of best luminescent thermometers consisting of Eu and Tb-based coordination polymers.[8] Nonetheless, it is worth noting that the number of luminescent thermometers based on Dy^{III} complexes is still scarce and that the herein reported S_m can be considered among the best results published so far.

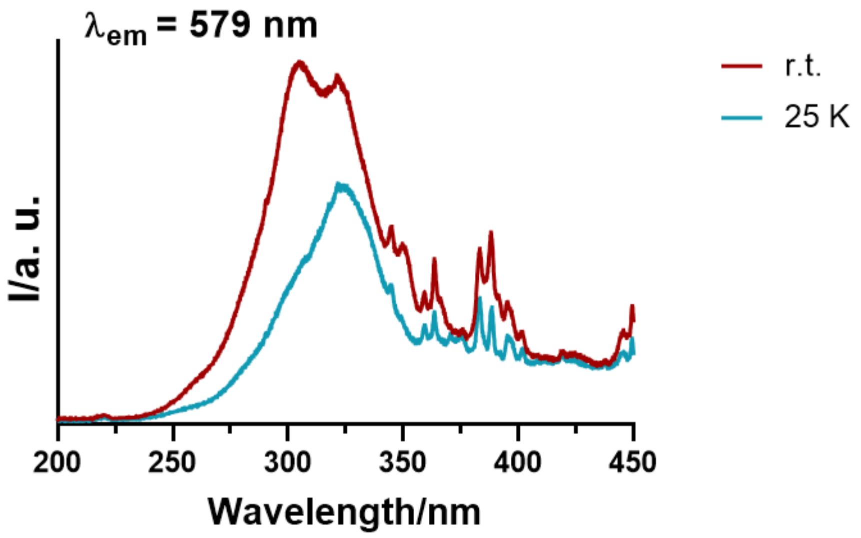

Figure S79.- For compound **1**, solid-state photoluminescence excitation spectra recorded at room temperature (brown) and 25 K (blue) monitored at λ_{em} = 579 nm.

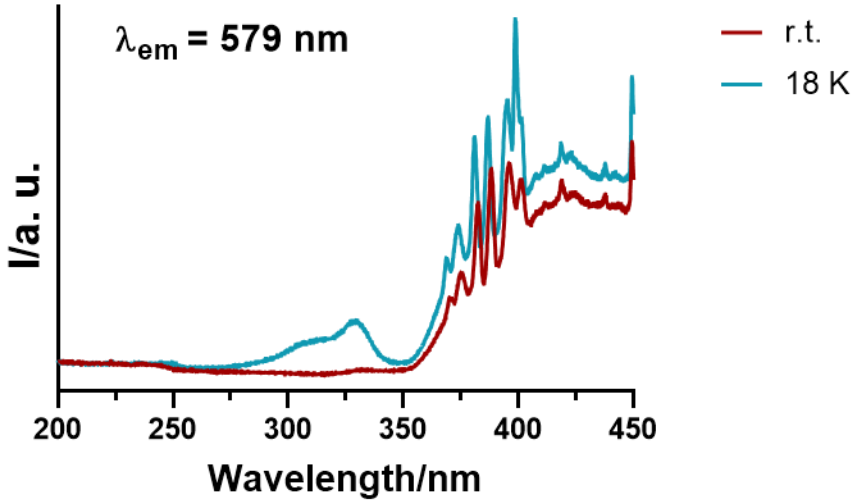

Figure S80.- For compound **2**, solid-state photoluminescence excitation spectra recorded at room temperature (brown) and 18 K (blue) monitored at λ_{em} = 579 nm.

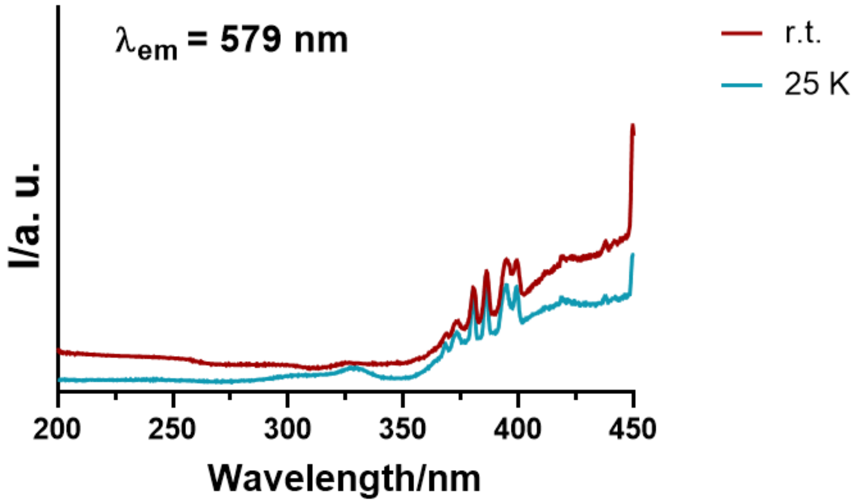

Figure S81.- For compound **3**, solid-state photoluminescence excitation spectra recorded at room temperature (brown) and 25 K (blue) monitored at λ_{em} = 579 nm.

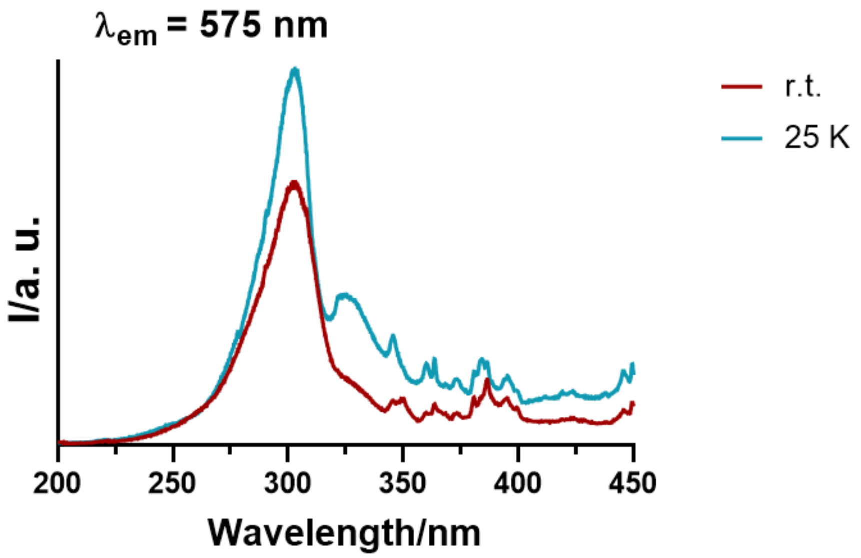

Figure S82.- For compound **4**, solid-state photoluminescence excitation spectra recorded at room temperature (brown) and 25 K (blue) monitored at λ_{em} = 575 nm.

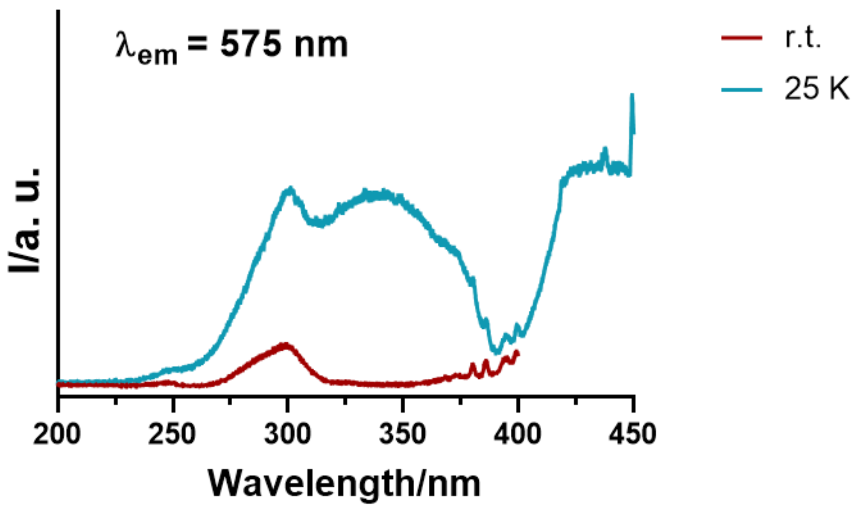

Figure S83.- For compound **5**, solid-state photoluminescence excitation spectra recorded at room temperature (brown) and 25 K (blue).

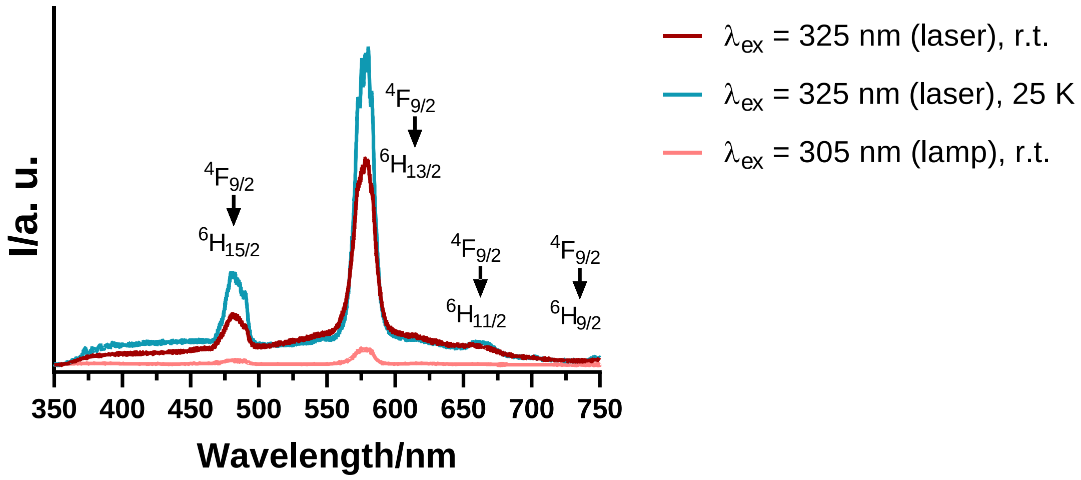

Figure S84.- For compound **1**, solid-state photoluminescence emission spectra recorded at room temperature (brown and pink) and 25 K (blue). Note that the excitation at 325 nm is provided by a laser, whereas at 305 nm is provided by a lamp. Thus, even though a more effective sensitization is expected at 305 nm according to the excitation spectrum, the power of the laser compensates using a less appropriate wavelength.

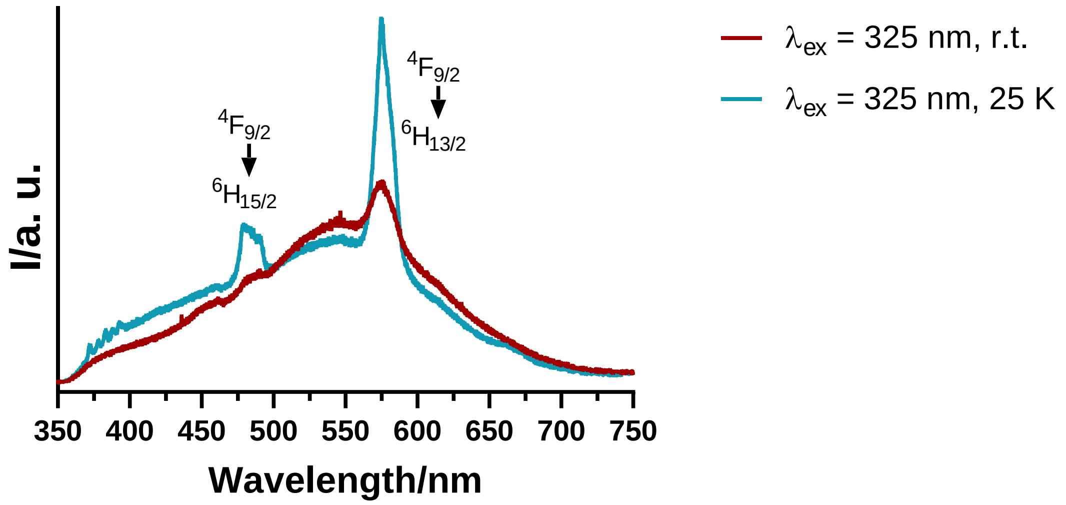

Figure S85.- For compound **2**, solid-state photoluminescence emission spectra recorded at room temperature (brown) and 25 K (blue).

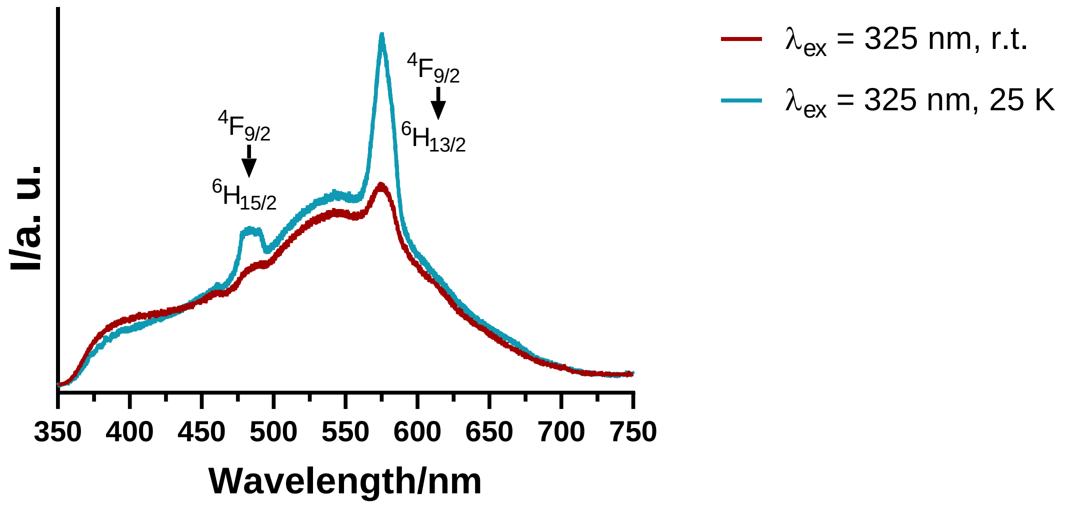

Figure S86.- For compound **3**, solid-state photoluminescence emission spectra recorded at room temperature (brown) and 25 K (blue).

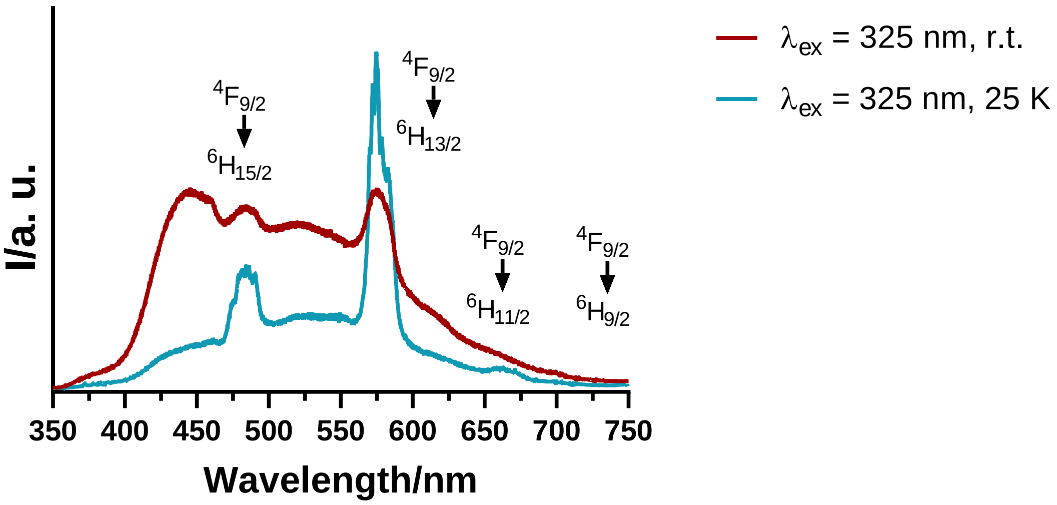

Figure S87.- For compound **5**, solid-state photoluminescence emission spectra recorded at room temperature (brown) and 25 K (blue).

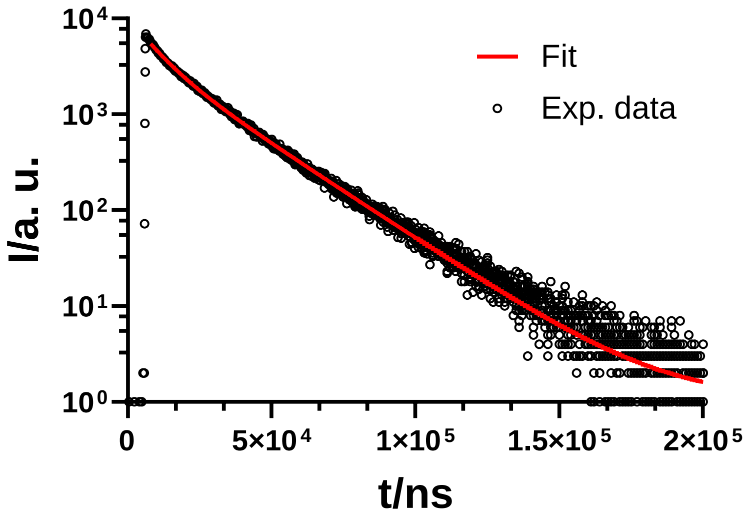

Figure S88.- Dy-based emission decay curve fitted for compound **1** at room temperature.

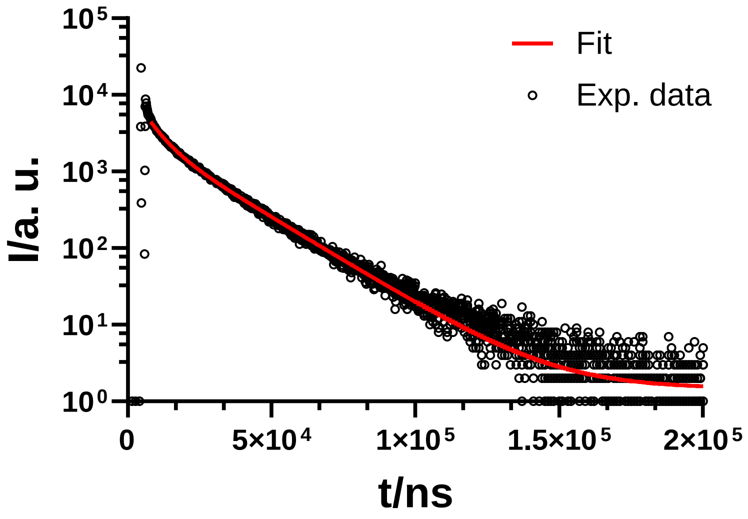

Figure S89.- Dy-based emission decay curve fitted for compound **1** at 25 K.

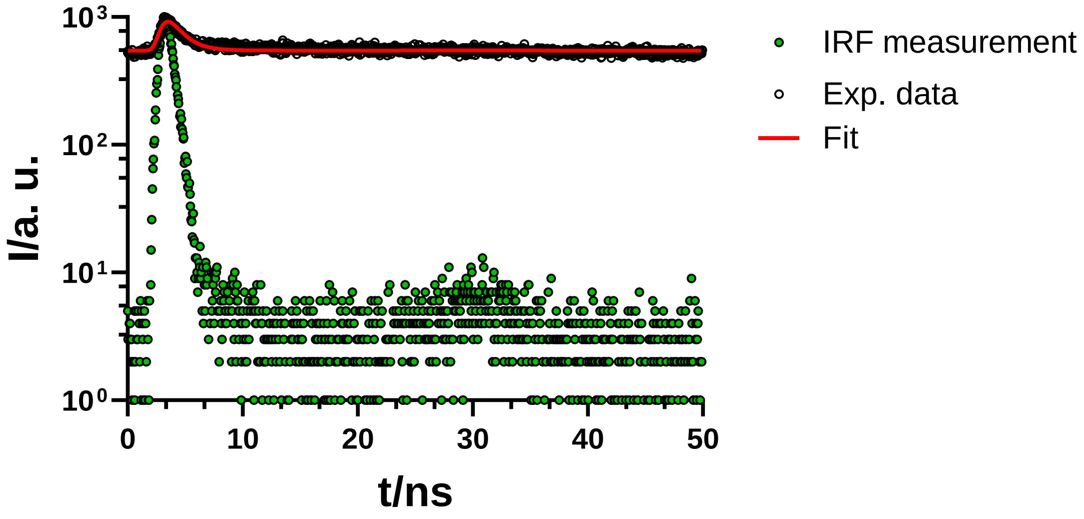

Figure S90.- Ligand-based emission decay curve fitted for compound **2** at 25 K.

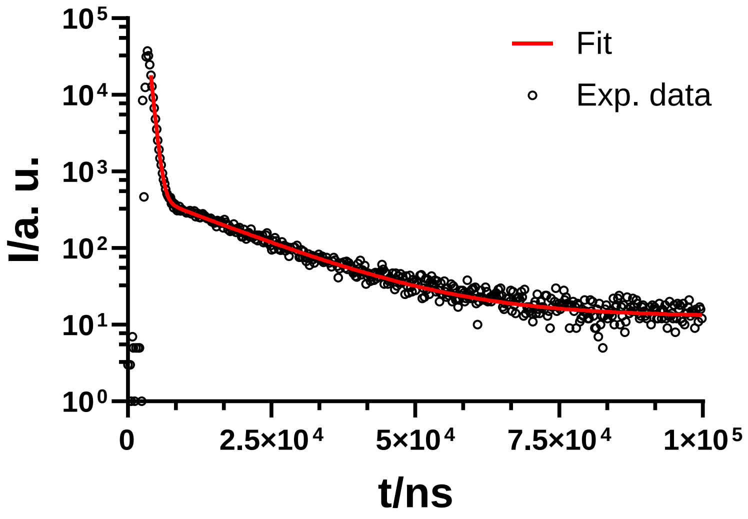

Figure S91.- Dy-based emission decay curve fitted for compound **2** at 14 K.

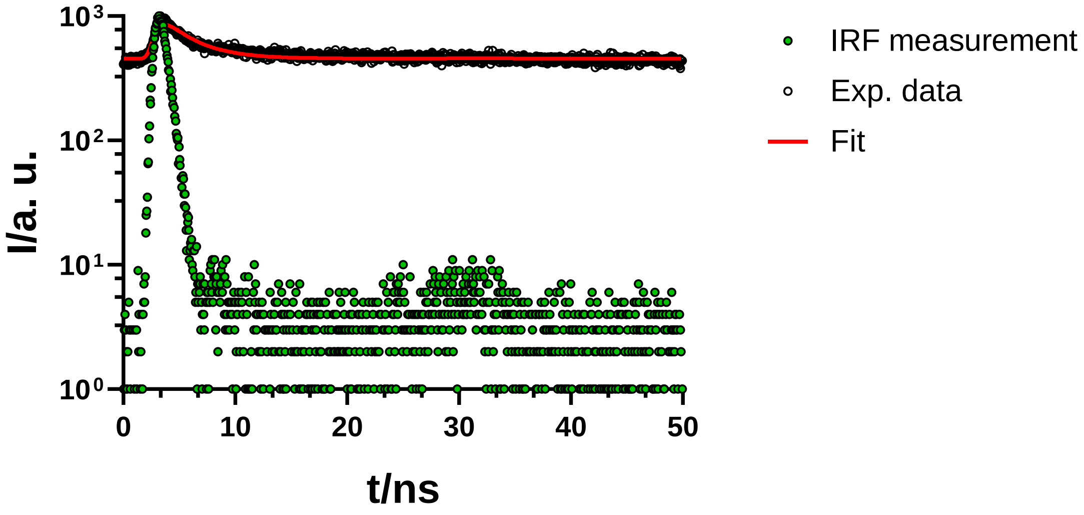

Figure S92.- Ligand-based emission decay curve fitted for compound **3** at 25 K.

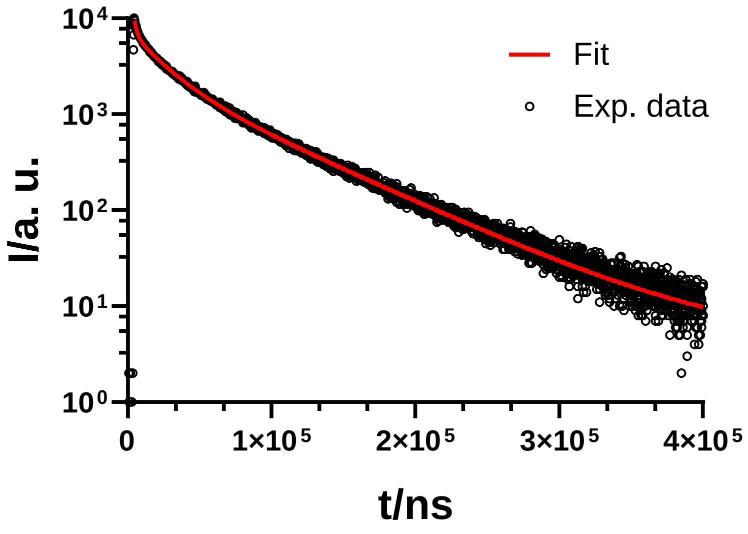

Figure S93.- Dy-based emission decay curve fitted for compound **3** at 25 K (λ_{ex} = 328 nm).

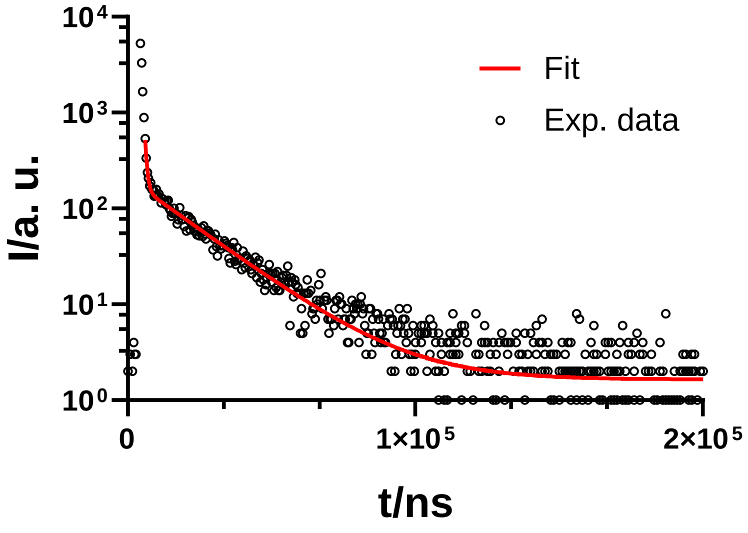

Figure S94.- Dy-based emission decay curve fitted for compound **3** at 25 K (λ_{ex} = 386 nm).

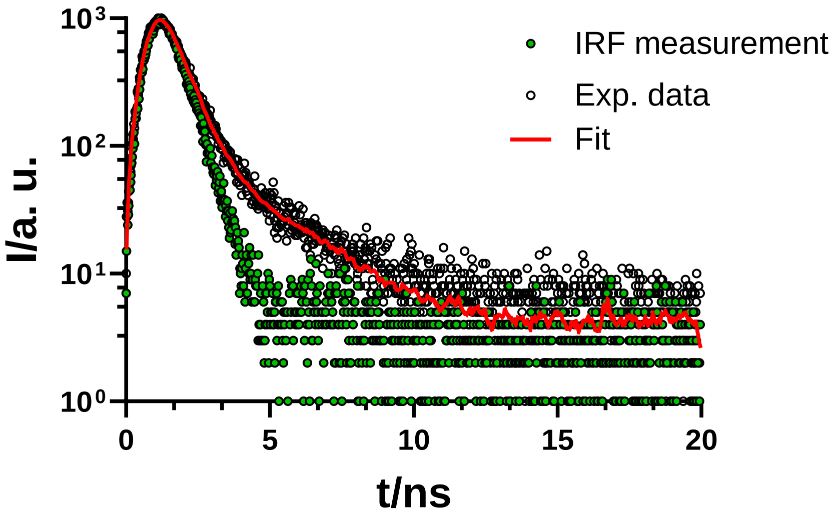

Figure S95.- Ligand-based emission decay curve fitted for compound **4** at 25 K.

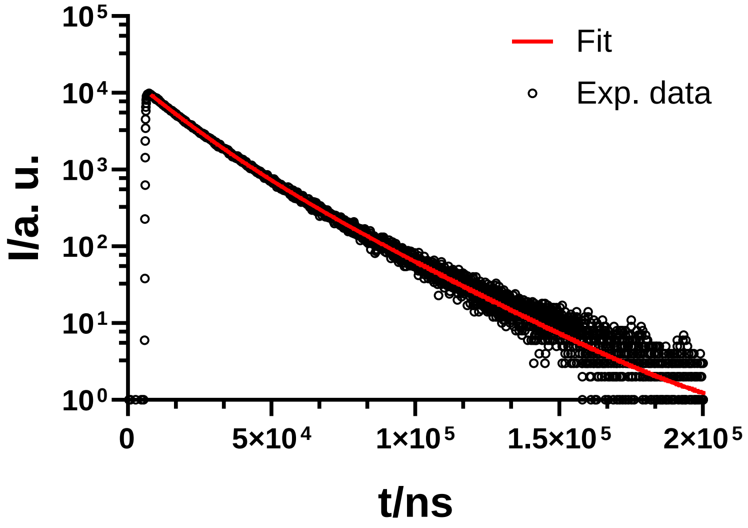

Figure S96.- Dy-based emission decay curve fitted for compound **4** at room temperature.

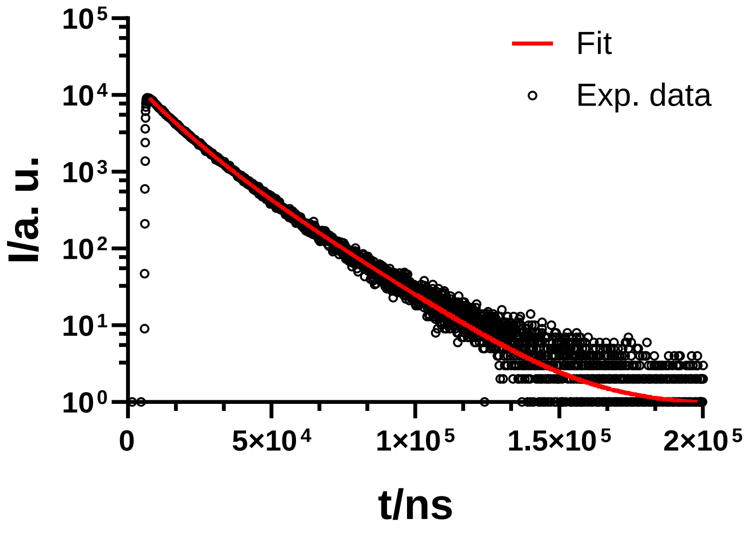

Figure S97.- Dy-based emission decay curve fitted for compound **4** at 25 K.

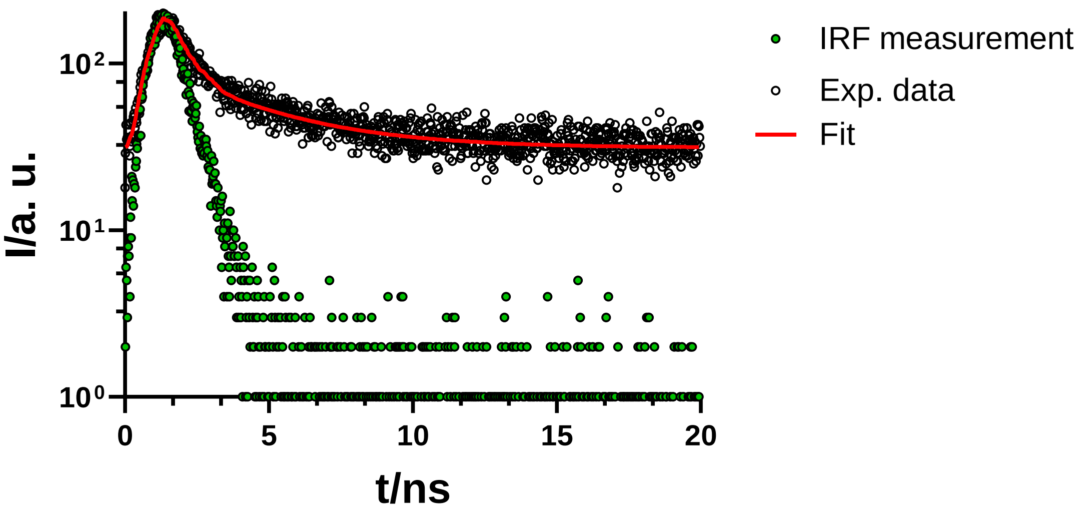

Figure S98.- Ligand-based emission decay curve fitted for compound **5** at room temperature.

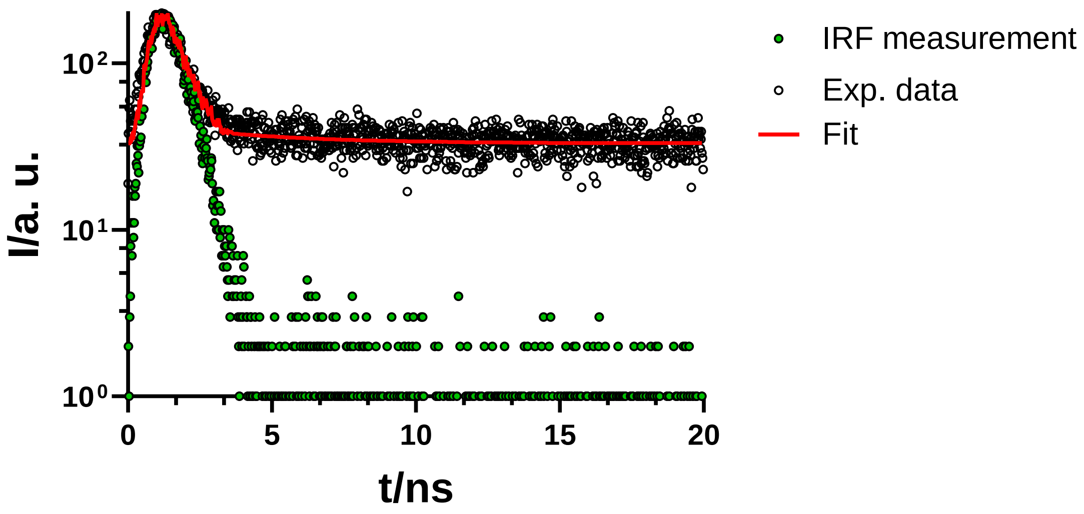

Figure S99.- Ligand-based emission decay curve fitted for compound **5** at 25 K.

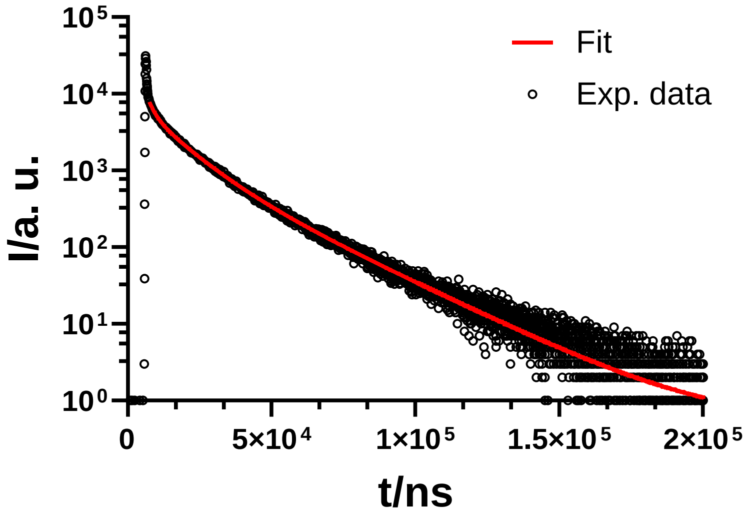

Figure S100.- Dy-based emission decay curve fitted for compound **5** at room temperature.

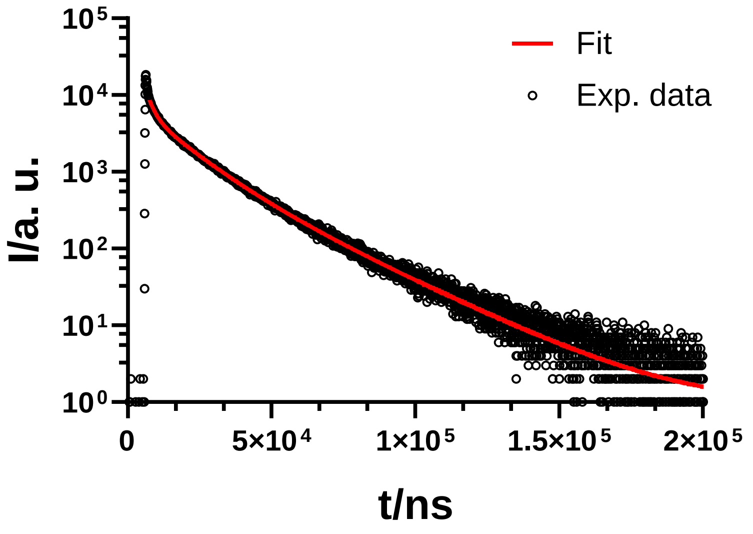

Figure S101.- Dy-based emission decay curve fitted for compound **5** at 25 K.

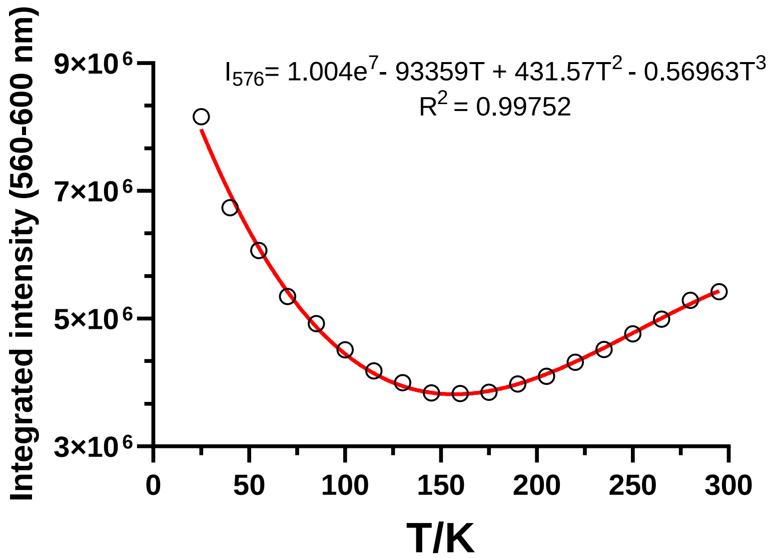

Figure S102.- Variable temperature behaviour of the integrated emission of the main Dy-centred characteristic band of compound **2** with the best fitting.

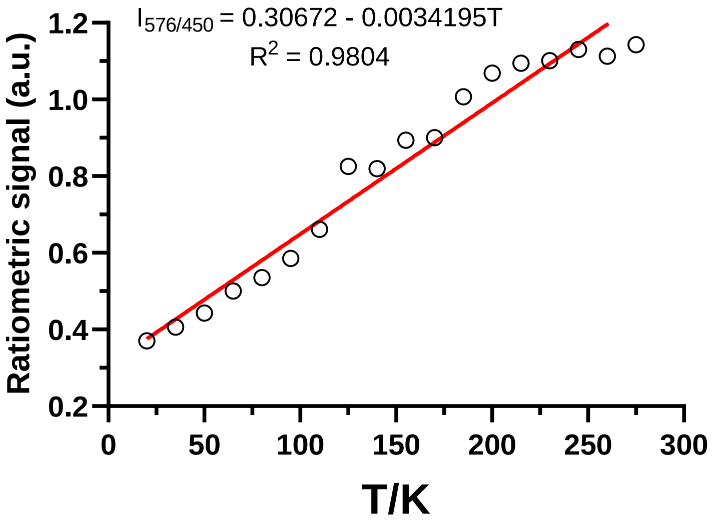

Figure S103.- Thermometric evolution of the ratiometric signal based on the integrated emission of the main Dy-centred (that emitting with maxima at 576 nm) and the ligand-centred (that emitting with maxima at 450 nm) bands of compound **2** showing the best fitting.

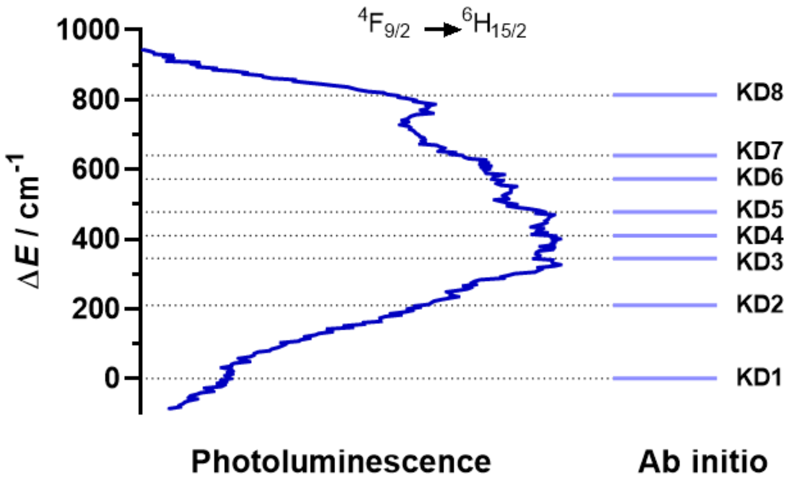

Figure S104.- For compound **1**, the $^4F_{9/2} \rightarrow {}^6H_{15/2}$ emission band recorded at 25 K in the relative energy scale (left) and energies of Kramers doublets according to *ab initio* calculations (right).

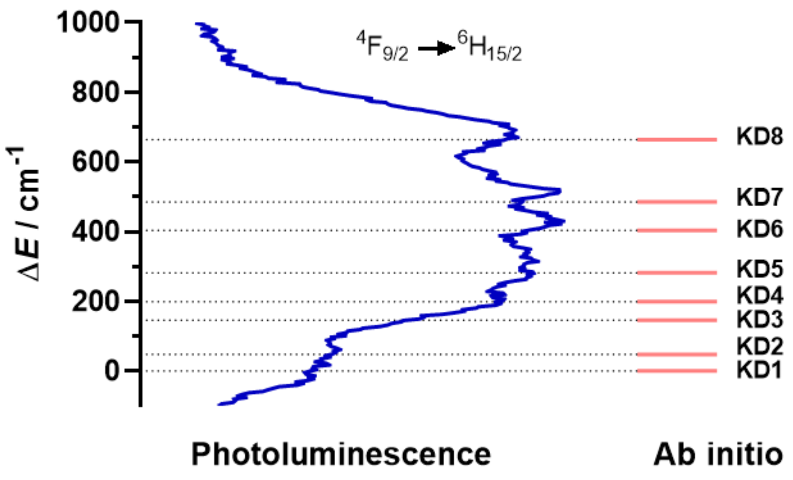

Figure S105.- For compound **5**, the $^4F_{9/2} \rightarrow {}^6H_{15/2}$ emission band recorded at 25 K in the relative energy scale (left) and energies of Kramers doublets according to SA-CASSCF(9,7)/CASPT2/SO-RASSI calculations (right).

S8. References

1 A. Zabala-Lekuona, X. Lopez de Pariza, I. F. Díaz-Ortega, J. Cepeda, H. Nojiri, N. P. Gritsan, A. A. Dmitriev, A. López-Ortega, A. Rodríguez-Diéguez, J. M. Seco and E. Colacio, *Dalt. Trans.*, 2024, **53**, 7971–7984.

2 D. Aravena, *J. Phys. Chem. Lett.*, 2018, **9**, 5327–5333.

3 D. Garanin and E. Chudnovsky, *Phys. Rev. B*, 1997, **56**, 11102.

4 A. Castro-Alvarez, Y. Gil, L. Llanos and D. Aravena, *Inorg. Chem. Front.*, 2020, **7**, 2478–2486.

5 A. Beeby, I. M. Clarkson, R. S. Dickins, S. Faulkner, D. Parker, L. Royle, A. S. de Sousa, J. A. Gareth Williams and M. Woods, *J. Chem. Soc. Perkin Trans. 2*, 1999, 493–504.

6 A. K. Singh, S. K. Singh, H. Mishra, R. Prakash and S. B. Rai, *J. Phys. Chem. B*, 2010, **114**, 13042–13051.

7 L. Song, J. Yu, K. Fang, F. Shi, W. Wan, L. Hao, Z. Zhao, W. Chen and Y. Xia, *ChemPhysChem*, 2022, **23**, e202100888.

8 J. Rocha, C. D. S. Brites and L. D. Carlos, *Chem. - A Eur. J.*, 2016, **22**, 14782–14795.
